# Cellular Validation of a Chemically Improved Inhibitor Identifies Monoubiquitination on OTUB2

**DOI:** 10.1021/acschembio.3c00227

**Published:** 2023-08-29

**Authors:** Jin Gan, Jelle de Vries, Jimmy J. L. L. Akkermans, Yassene Mohammed, Rayman T. N. Tjokrodirijo, Arnoud H. de Ru, Robbert Q. Kim, David A. Vargas, Vito Pol, Rudi Fasan, Peter A. van Veelen, Jacques Neefjes, Hans van Dam, Huib Ovaa, Aysegul Sapmaz, Paul P. Geurink

**Affiliations:** † Department of Cell and Chemical Biology, Division of Chemical Biology and Drug Discovery, Leiden University Medical Center, Einthovenweg 20, 2333 ZC Leiden, The Netherlands; ‡ Department of Cell and Chemical Biology and Oncode Institute, Leiden University Medical Center LUMC, Einthovenweg 20, 2333 ZC Leiden, The Netherlands; § Center for Proteomics and Metabolomics, Leiden University Medical Center, Albinusdreef 2, 2333 ZC Leiden, The Netherlands; ∥ Department of Chemistry, University of Rochester, Hutchison Hall, 120 Trustee Rd, Rochester, New York 14627, United States

## Abstract

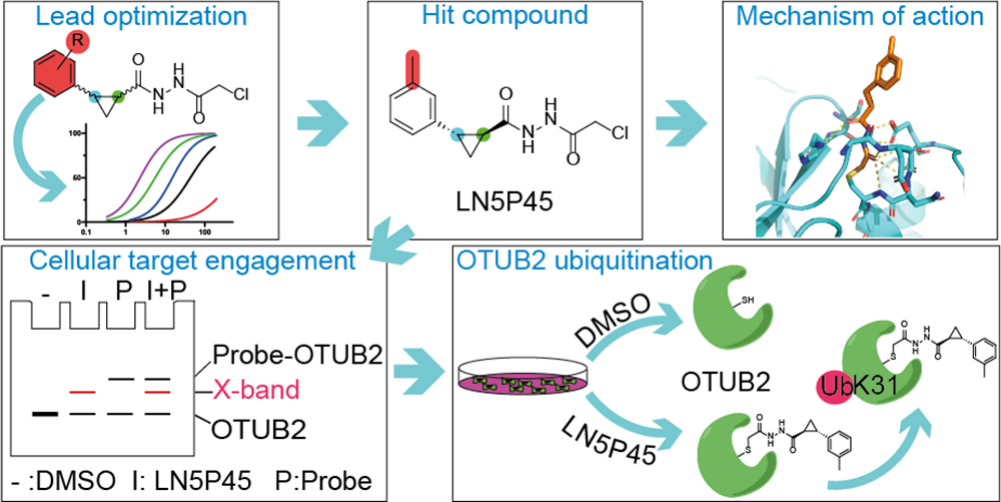

Ubiquitin thioesterase OTUB2, a cysteine protease from the ovarian tumor (OTU) deubiquitinase superfamily, is often overexpressed during tumor progression and metastasis. Development of OTUB2 inhibitors is therefore believed to be therapeutically important, yet potent and selective small-molecule inhibitors targeting OTUB2 are scarce. Here, we describe the development of an improved OTUB2 inhibitor, **LN5P45**, comprising a chloroacethydrazide moiety that covalently reacts to the active-site cysteine residue. **LN5P45** shows outstanding target engagement and proteome-wide selectivity in living cells. Importantly, **LN5P45** as well as other OTUB2 inhibitors strongly induce monoubiquitination of OTUB2 on lysine 31. We present a route to future OTUB2-related therapeutics and have shown that the OTUB2 inhibitor developed in this study can help to uncover new aspects of the related biology and open new questions regarding the understanding of OTUB2 regulation at the post-translational modification level.

## Introduction

Ubiquitination is an essential post-translational protein modification in a plethora of cellular processes. Ubiquitin generally modifies target proteins through lysine residues, and occasionally on cysteine, serine and threonine residues, which is catalyzed by the sequential action of E1, E2, and E3 enzymes.^1−3^ Protein ubiquitination can have an impact on cellular signaling, localization, and function and interaction of target proteins or mark substrates for proteasomal degradation.^4^ Deubiquitinases (DUBs) can reverse this process by removing conjugated ubiquitin from substrate proteins.^5^ A tightly regulated balance between ubiquitination and deubiquitination processes is critical for cellular homeostasis and dynamics. Post-translational modification of DUBs, including phosphorylation, ubiquitination, SUMOylation, and acetylation, can further fine-tune these processes.^6^

Ovarian tumor (OTU) domain-containing ubiquitin aldehyde-binding protein 2 (OTUB2) is an active-site cysteine containing DUB.^7^ Recent research progress revealed that elevated OTUB2 expression correlates with tumorigenesis and metastasis.^8−11^ OTUB2 can promote tumorigenic progression of non-small cell lung cancer cells through deubiquitination of U2AF2, stimulation of the Warburg effect, and activation of AKT/mTOR signaling.^10^ Moreover, OTUB2 has been identified as a cancer stemness and metastasis-promoting factor that deubiquitinates YAP/TAZ proteins in breast cancer cells, whereby poly-SUMOylation on OTUB2 lysine 233 mediates the interaction with YAP/TAZ.^11^ These studies demonstrate OTUB2 to be a relevant therapeutic target, and the development of OTUB2-specific inhibitors can potentially be beneficial for cancer therapies.

Despite numerous publications on potent DUB inhibitors,^12−14^ there are hardly any reports on potent and selective inhibitors of OTUB2. A previous high-throughput electrophilic-fragment screening campaign enabled identification of covalent OTUB2 inhibitor OTUB2-COV1, which showed in-cell activity and good selectivity for OTUB2 within the DUB family.^15^ However, the biological application of this compound is limited due to its moderate potency, and its improvement was restricted due to a lack of density of key regions in the co-crystal structure of the OTUB2-inhibitor complex and unknown absolute stereochemistry of the inhibitor. Here, we report the development of improved OTUB2 inhibitors through optimization of the original OTUB2-COV1 inhibitor with respect to its stereochemistry and aromatic ring substituents. For our best inhibitor, **LN5P45**, we solved the X-ray co-crystal structure in complex with OTUB2, and we conducted a streamlined cysteine activity-based protein profiling (SLC-ABPP), which revealed that **LN5P45** selectively engages endogenous OTUB2 over other ubiquitin machinery components. Interestingly, while utilizing **LN5P45** in cells, we unexpectedly found that this OTUB2 inhibitor also has a unique secondary activity, the monoubiquitination of the inhibited OTUB2 on lysine 31. This suggests that OTUB2 inhibition may have more surprises, which can be further explored with this inhibitor.

## Results and Discussion

### OTUB2 Inhibitor Optimization

Previously, we showed that the chloroacethydrazide-containing molecule COV-1 (Table 1, compound **1**) can DUB-selectively inhibit OTUB2 with moderate potency in living cells, thereby forming an irreversible covalent bond with the OTUB2 active site cysteine (Cys51).^15^ To further optimize the inhibitory properties of this compound, we first turned our attention to elucidating the preferred stereochemistry of the substituents attached to the cyclopropane, as the exact nature of the stereocenters in COV-1 were unknown. COV-1 is synthesized in two or three steps from 2-phenylcyclopropane-1-carboxylate (**11**) or its methyl (**12**) or ethyl ester (**13**) derivatives, as shown in Scheme S1. The stereochemistry in the final product originates from the stereochemical nature of the starting compound and does not change during the course of the synthesis. We acquired three commercially available stereoisomers of 2-phenylcyclopropane-1-carboxylate (**11**): a racemic mixture of the *trans*-only isomers, a racemic mixture of the *cis*-only isomers, and the chirally pure isomer (1*S*,2*R*) from which compounds **2**–**4** were synthesized, respectively (Table 1). The inhibitory potency of these compounds was assessed in a biochemical OTUB2 enzyme activity assay using the fluorogenic substrate ubiquitin-rhodamine-morpholine (UbRhoMP).^16^ Recombinant human OTUB2^17^ was incubated with a serial dilution of each compound for 2.5 h before addition of the substrate, followed by subsequent recording of the fluorescence intensity over time. The percentage inhibition was calculated from the activity curves and normalized to the positive (10 mM *N*-ethylmaleimide, 100% inhibition) and negative (DMSO, 0% inhibition) controls. The inhibition was plotted against the compound concentration from which the IC_50_ values were calculated (Table 1, Figure S1A). Only the compounds with the *trans* configuration inhibited OTUB2. Virtually no inhibition was observed for the *cis* configuration compounds **3** and **4**, whereas the IC_50_ value for compound **2** is nearly identical to that of compound **1**. Hence, we reasoned that compound **1** most likely has the *trans* configuration and that this is required for OTUB2 inhibition.

**Table 1:**
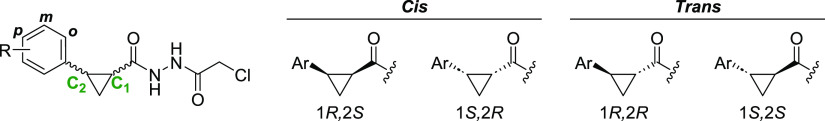
Overview of the Stereochemistry and Substituents of the Compounds along with Their OTUB2 Inhibition Values

compound	R	C_1_	C_2_	IC_50_ (μM)^a^	*k*_inact_/*K*_I_ (M^–1^ s^–1^)
**1** (**COV-1**)	H	unknown		15.4^*b*^	3.7^*b*^
**2**	H	mix of *trans* isomers		14.1 ± 1.8	ND^c^
**3**	H	mix of *cis* isomers		>100	ND
**4**	H	*S*	*R*	>100	ND
**5**	H	*R*	*R*	15.6 ± 0.5	4.8
**6**	H	*S*	*S*	6.8 ± 1.3	14.7
**7**	*o*-Me	*S*	*S*	43.0 ± 5.0	1.5
**8** (**LN5P45**)	*m*-Me	*S*	*S*	2.3 ± 0.4	45.7
**9**	*p*-Me	*S*	*S*	12.4 ± 0.2	6.5
**10**	3,4-diF	*S*	*S*	10.1 ± 0.3	8.5

a Determined after 2.5 h incubation (average and ±s.d. of *n* = 3 independent experiments).

b As reported in ref (15).

c Not determined.

Next, we examined whether one of the *trans* stereoisomers would be favored. The required chirally pure *trans* ethyl 2-phenylcyclopropane-1-carboxylate (**13**) starting compounds were obtained *via* enantioselective cyclopropanation of styrene and α-ethyl diazoacetate (EDA) using engineered myoglobin (Mb) carbene transferases developed by the Fasan lab (Scheme S2).^18−20^ Both *trans* enantiomers were produced enzymatically in enantiopure form and further processed to obtain the corresponding chloroacethydrazide compounds **5** (1*R*,2*R*) and **6** (1*S*,2*S*), which were tested for OTUB2 inhibition. From the obtained IC_50_ values (Table 1, Figure S1A), it became apparent that, although both stereoisomers inhibit OTUB2, compound **6** having the (1*S*,2*S*) configuration is the most potent one with an IC_50_ value of 6.8 μM, which makes it about twice as potent compared to its enantiomer compound **5** and the initial COV-1 compound.

After having determined the (1*S*,2*S*) configuration as the preferred stereoisomer, we turned our attention to the aromatic ring substituents to further improve the compound potency. Applying the same chemo-enzymatic strategy described above, a panel of aryl substituted *trans* (1*S*,2*S*) cyclopropane compounds **7**–**10** were generated having a methyl (**7**–**9**) or fluorine (**10**) substituent at different positions on the aromatic ring. The compounds were tested for their OTUB2 inhibitory potency using the assay as described above (Table 1, Figure S1A). Introduction of a methyl group at the *para* position (compound **9**) or a *meta*, *para*-difluoride (compound **10**) had little effect on IC_50_, but the *ortho*-methyl group had a detrimental effect (compound **7**). Interestingly, introducing a *meta*-methyl group (compound **8**) increased the inhibitory potency, resulting in an IC_50_ of 2.3 μM. Since these compounds are all covalent, irreversible inhibitors,^15^ the IC_50_ depends on the incubation time. To get a better estimate of their respective inhibitory capacity, independent of incubation time, we determined the *k*_inact_/*K*_I_ values of compounds **5**–**10** by pre-mixing the compounds with the substrate before adding OTUB2. The obtained values corroborated well with the IC_50_ values (Table 1, Figure S1B), again confirming compound **8** to be the most potent, which was renamed **LN5P45** for further experiments and to ease future reference. The *in vitro* selectivity of **LN5P45** for OTUB2 was assessed using a panel containing six DUBs from the OTU family, including its closest family members OTUB1, OTUD1, OTUD2, OTUD3, OTUD6B, and Cezanne, and two DUBs from outside this family, UCHL1 and USP16 (Figure S2, Table S1). Most of these DUBs were not inhibited by **LN5P45** at concentrations up to 150 μM, and only marginal inhibition was observed for UCHL1 and USP16 with IC_50_ values above 100 μM. A slightly better potency was found for OTUD3 with an IC_50_ value of 56 μM, which demonstrates **LN5P45** to be about 20-fold more potent toward OTUB2.

**Figure 1:**
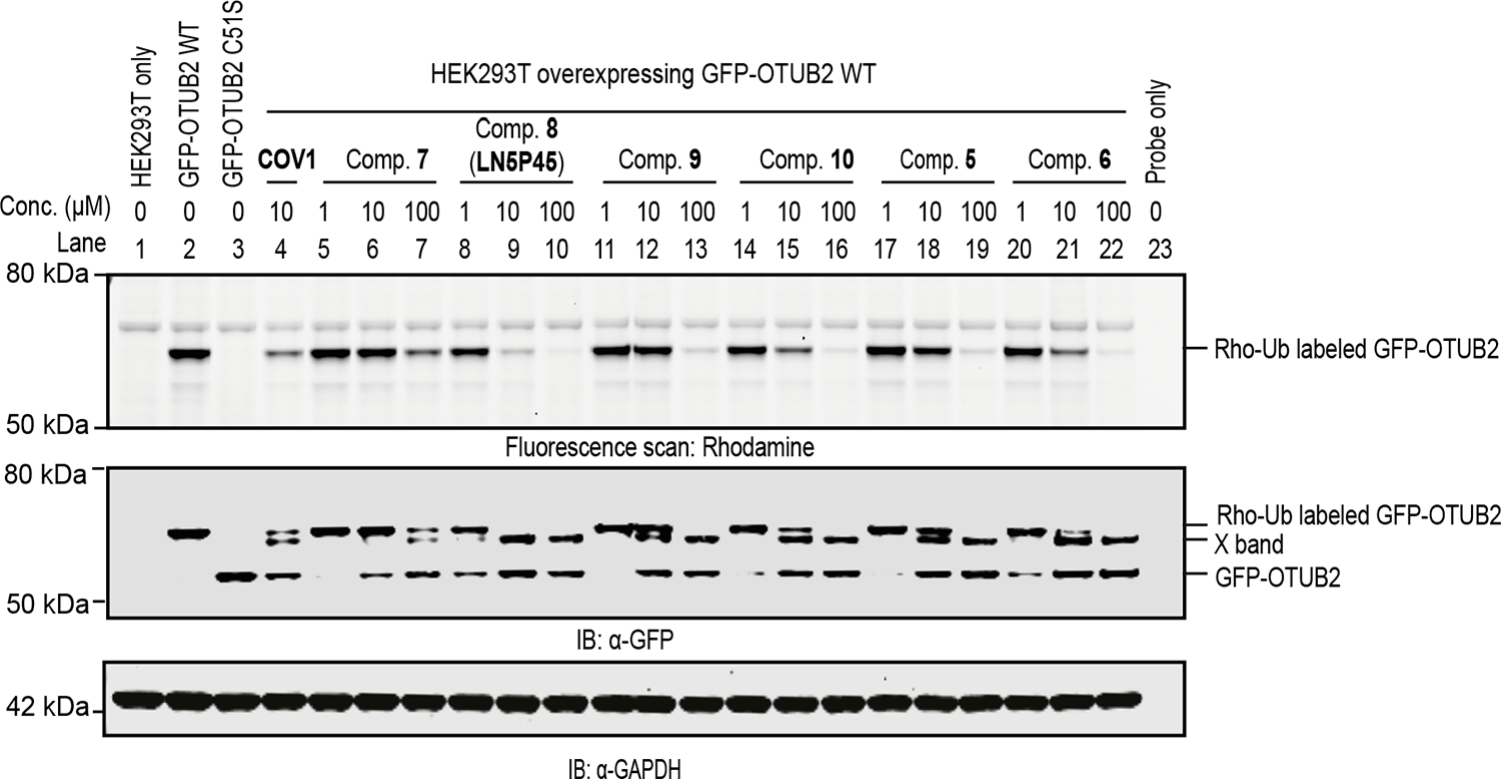
Inhibition of GFP-OTUB2 by the indicated compounds in HEK293T cells. GFP-OTUB2 (WT or catalytic dead mutant C51S) was transiently transfected in HEK293T cells. After 24 h, cells were treated with the indicated inhibitors and concentrations for 4 h. Cell lysates were incubated with the Rho-Ub-PA probe and subjected to SDS-PAGE, gel fluorescence scanning, and immunoblotting. Top: fluorescence scan of the gel region containing Rho-Ub-PA probe-labeled GFP-OTUB2. Middle: anti-GFP immunoblot showing probe-labeled and unlabeled GFP-OTUB2. (An extra band is annotated as “X band”, see results in Figure 4). Bottom: anti-GAPDH immunoblot data validating equal loading of each sample.

Besides the biochemical inhibition potency, we were also interested in determining the ability of the compounds to inhibit OTUB2 in live cells. To assess this, we performed gel-based competitive activity-based protein profiling (ABPP) for compounds **5**–**10** in a similar assay as described previously for COV-1.^15^ HEK293T cells overexpressing GFP-OTUB2 were incubated with DMSO or increasing concentrations of the OTUB2 inhibitors, followed by cell lysis and co-incubation with fluorescently tagged activity-based probe Rho-Ub-PA.^21^ This probe covalently labels the active site cysteine of DUBs that are not already blocked by inhibitors. The labeled and unlabeled GFP-OTUB2 were resolved by SDS-PAGE, imaged by fluorescence scanning, and further analyzed by anti-GFP immunoblot analysis (Figure 1). Compared to the DMSO control sample, all inhibitors were engaged with cellular GFP-OTUB2 in a dose-dependent manner, indicating that they all can efficiently penetrate the cell membrane. Compounds **6**, **10**, and **LN5P45** displayed clear inhibition from 10 μM, which corresponded well to their *in vitro* IC_50_ values (Table 1). More importantly, **LN5P45** outperformed the other inhibitors both *in vitro* and in cells and we therefore decided to continue with this compound.

### Determination of the Co-crystal Structure of OTUB2 in Complex with **LN5P45**

**Figure 2:**
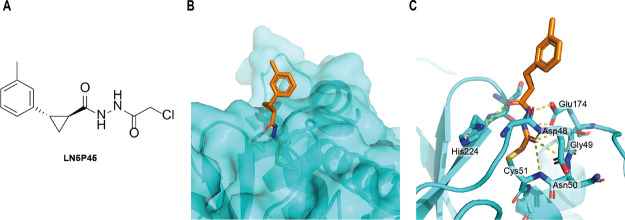
Characterization of the interactions between OTUB2 and inhibitor **LN5P45**. (A) Chemical structure of **LN5P45**. (B) Surface structure of human OTUB2 (azure surface) in complex with **LN5P45** (brown sticks) showing that the *trans* substituted cyclopropane ring of **LN5P45** points the phenyl ring outside the protein. (C) Cartoon structure of the binding pockets of **LN5P45** (brown sticks) in the active site of OTUB2 (azure ribbons). The key residues interacting with **LN5P45** are shown as sticks, and hydrogen bonds are shown in yellow dashed lines (PDB: 8CMS).

We next set out to evaluate the binding mode of **LN5P45** within the OTUB2 active site. LC–MS analysis of OTUB2 before and after reaction with **LN5P45** confirmed the formation of a covalent complex by showing a mass difference of 230 Da, which corresponds to a substitution reaction at the chloride (Figure S3). To further obtain information on the structural aspects of the interactions, we solved the co-crystal structure of OTUB2 in complex with **LN5P45** at 1.77 Å resolution (Figure 2, Figure S4, Table S2, PDB 8CMS). In contrast to the co-crystal structure of the OTUB2-COV-1 complex (PDB 5QIO), a clear density for the cyclopropyl-phenyl moiety in our OTUB2-**LN5P45** co-crystal structure was detected, which is likely due to the fact that **LN5P45** has the defined (1*S*,2*S*) configuration. The phenyl ring moiety points outside the protein toward the solvent and has as such no obvious interactions (Figure 2B); there are no aromatic protein residues or arginine around the active site that could π–π stack with the phenyl moiety of **LN5P45**. This unfortunately also did not yield any clues as to why the *meta*-substituted phenyl is preferred over the *para*-variant (compound **9**). Its orientation, away from the protein surface, however could explain our earlier observation that only the compounds with the *trans* substituted cyclopropane ring (compounds **5**–**10**) and not the *cis*-linked ones (compounds **3** and **4**) inhibited OTUB2. The latter situation would likely result in a conformational clash of the phenyl group with the protein. This could also explain the finding that the *ortho*-methyl-substituted compound **7** showed diminished inhibition. The *trans* orientation on the other hand nicely positions the inhibitor inside the small groove leading to the active site. When comparing the co-crystal structure to the initial compound COV-1 5QIO, both the phenyl moieties point outward of the protein, while for **LN5P45**, we actually found the electron density to validate this position (contoured at 1σ, Figure S4). The binding of the acethydrazide part with the OTUB2 active site compared well with earlier findings from the PDB 5QIO structure (Figure 2C). Active site Cys51 is covalently bound (distance is 1.7 Å) to the acetamide-Cα, and the acetamide carbonyl occupies the oxyanion hole formed by the backbone NH of residues Asp48, Gly49 (note that this is misannotated as Arg49 in the PDB 5QIO structure), Asn50, and Cys51. The carbonyl directly attached to the cyclopropane ring interacts with the side chain of His224 through a hydrogen bond, and the Glu174 side chain carboxylate also forms hydrogen bonds with both of the hydrazide amines.

### **LN5P45** Selectively Engages Endogenous OTUB2 over Other Ubiquitin Machinery Components

**Figure 3:**
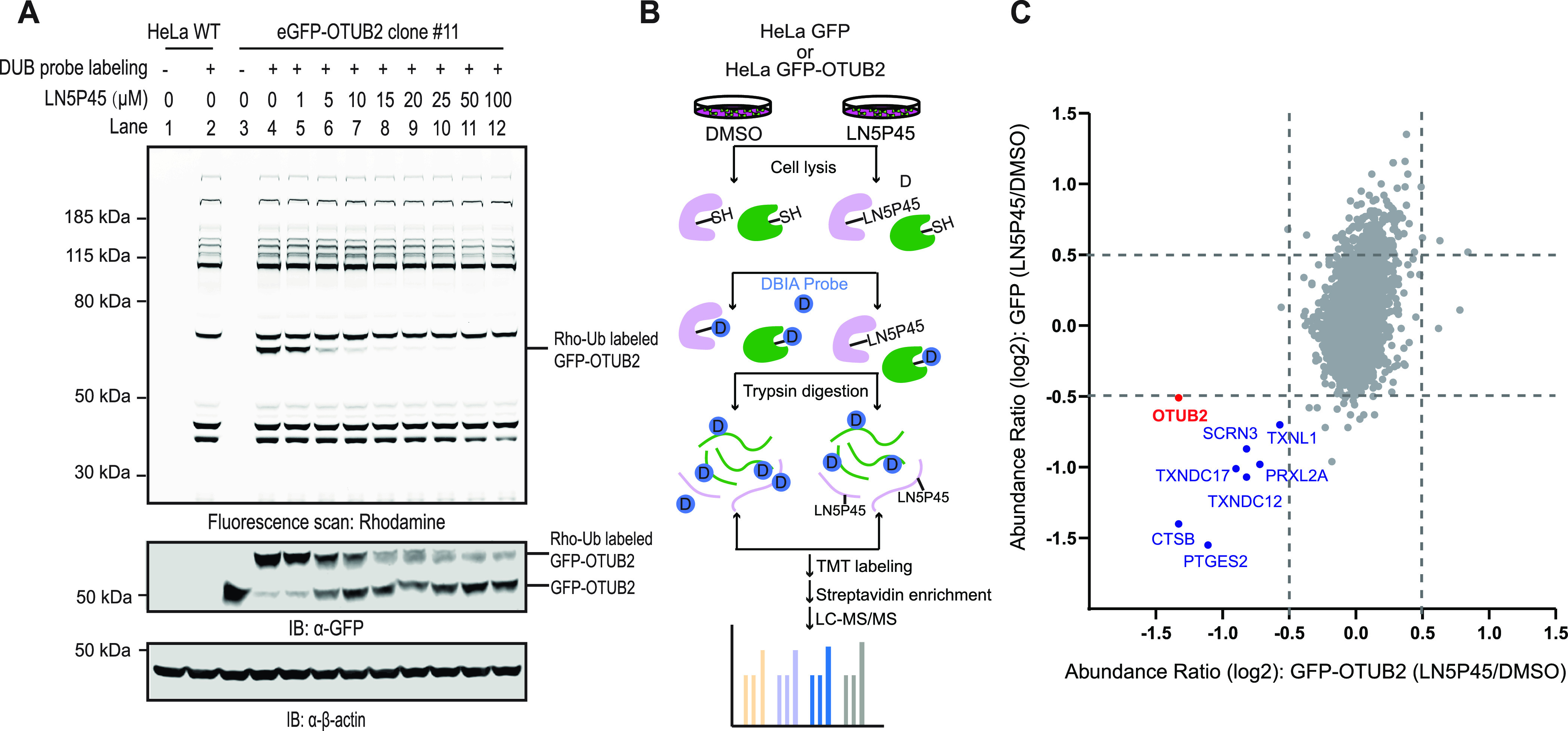
**LN5P45** engages endogenous OTUB2 and is selective for OTUB2 over other ubiquitin machinery components. (A) HeLa cells expressing GFP-tagged endogenous OTUB2 (clone #11) were treated with the indicated concentrations of **LN5P45** for 4 h, followed by cell lysis, incubation with the Rho-Ub-PA DUB probe, SDS-PAGE, gel fluorescence scanning, and immunoblotting. Top: fluorescence scan of Rho-Ub-PA probe-labeled DUBs. Middle: anti-GFP immunoblot data corresponding to probe-labeled and unlabeled GFP-OTUB2. Bottom: anti-β actin immunoblot data validating equal loading of each sample. (B) Schematic representation of the SLC-ABPP method based on TMT labeling. Subsequent steps of the procedure consist of treatment of cells with 10 μM **LN5P45** or DMSO for 4 h, cell lysis, treatment with the 500 μM DBIA probe for 1 h, TMT labeling, combining the samples, neutravidin bead enrichment for biotinylated cysteine-containing peptides, followed by mass spectrometry analysis. (C) Abundance plots of the 10,670 cysteine sites modified by the DBIA probe in **LN5P45**- versus DMSO-treated HeLa cells overexpressing GFP or GFP-OTUB2; each sample was prepared in quadruplicates. The relative abundance between the groups from the TMT labels associated with each measured sample was obtained. The relative abundance was in relation to **LN5P45** versus DMSO and included all confident identifications. The log2 fold change of HeLa overexpression GFP-OTUB2 (*x*-axis) was compared to the log2 fold change of HeLa overexpression GFP (*y*-axis). The peptides with log2 fold change between −0.5 and 0.5 showed similar fold changes in both HeLa overexpression GFP-OTUB2 and GFP conditions. Only the peptides with <−0.5 log2 fold change in both conditions were considered potential targets of **LN5P45** (blue), including OTUB2 (red).

To validate that **LN5P45** also can engage with endogenous OTUB2, we used the bone-metastatic (BM) derivative of breast cancer cell line MDA-MB-231, which was reported to express relatively high levels of OTUB2 compared to other breast cancer lines.^11^ Because of the lack of a good antibody that selectively recognizes endogenous OTUB2, siRNA-mediated depletion of endogenous OTUB2 was performed as a control to confirm OTUB2 labeling with our Rho-Ub-PA probe, and depletion of OTUB2 was confirmed by real time quantitative polymerase chain reaction (RT-qPCR). This enabled the identification of a specific protein band (around 35 kDa) that was substantially reduced in the OTUB2-depleted sample (Figure S5). The observed molecular weight of this labeled protein matched with the expected molecular weight of Rho-Ub-PA-labeled OTUB2 (OTUB2 27.2 kDa + 8 kDa Ub probe). Moreover, upon incubation of living BM MDA-MB-231 cells with 10 or 20 μM **LN5P45** for 4 h, the Rho-Ub-PA-labeled OTUB2 band almost disappeared compared to the DMSO control sample (Figure S5A). Since none of the other Rho-Ub-PA-labeled bands were affected by the OTUB2 inhibitor, this result indicates that **LN5P45** can specifically inhibit endogenous OTUB2. Next, we endogenously tagged OTUB2 with GFP in HeLa cells using CRISPR-Cas9.^22,23^ Three independent GFP-positive clones (#9, #11, and #32) were validated by OTUB2-specific siRNA treatment and subsequent anti-GFP immunoblotting and flow cytometry (Figure S6). As clone #9 cells mainly expressed a smaller non-specific GFP-containing protein, we only used clones #11 and #32 for subsequent studies. Like the transiently transfected HEK293T cells (Figure 1), the eGFP-OTUB2 HeLa clones were treated with increasing concentrations of **LN5P45** for 4 h, prior to analysis by Rho-Ub-PA labeling. Nearly complete inhibition of endogenous GFP-OTUB2 was observed starting from 5 μM (Figure 3A, Figure S6C).

The Rho-Ub-PA profile obtained for the HeLa cells suggested that OTUB2 is the only DUB target of **LN5P45** up to 25 μM in these cells (Figure 3A, Figure S6C). To further investigate the selectivity of **LN5P45** throughout the proteome, we performed SLC-ABPP to determine its cysteine targets.^24^ For this, we incubated HeLa cells that ectopically expressed either GFP or GFP-OTUB2 WT with either DMSO or 10 μM **LN5P45**, followed by cell lysis and incubation with a desthiobiotin iodoacetamide (DBIA) probe that is used to distinguish reactive cysteine sites that are not bound to **LN5P45** (Figure 3B). Further, peptides generated by trypsin from replicates of each sample were labeled using TMT16-plex to perform TMT-based quantitative proteomics profiling. Following the TMT labeling, the peptides conjugated to the DBIA probe were enriched by NeutrAvidin beads and analyzed by LC–MS/MS. A total of 10,670 peptides containing reactive cysteine sites were detected and labeled according to their abundance ratios (**LN5P45** versus DMSO treated) for both the HeLa cells expressing GFP and GFP-OTUB2 (Figure 3C). As expected, binding of the DBIA probe to OTUB2 (C51) was inhibited in the presence of **LN5P45**. In addition, DBIA probe binding to seven proteins not involved in ubiquitination was inhibited. These findings confirm that **LN5P45** engages cellular OTUB2 and is selective for OTUB2 over other DBIA probe-binding ubiquitin machinery components at 10 μM.

### **LN5P45** Treatment Results in Monoubiquitination of OTUB2 on Lysine 31

Interestingly, the Western blot analysis of GFP-OTUB2 in Figure 1 showed an additional GFP-OTUB2 band upon inhibitor treatment (annotated as “X band” in Figure 1). Since this might represent a modified version of OTUB2 induced by its inactivation, we further examined its appearance and its nature. Comparison of the effects of two different OTUB2 inhibitors in living HEK293T cells versus cell lysates showed that (i) X band appearance was independent of Rho-Ub-PA probe labeling, (ii) a weak X band can already be detected in the DMSO-treated GFP-OTUB2 WT expressing cells, and (iii) only in living cells that the X band is increased by inhibitor treatment and also by disruption of the OTUB2 catalytic activity through genetic inactivation (C51S). Moreover, Rho-Ub-PA probe labeling did not affect the abundance of X band in the inhibitor-treated living cells (Figure S7A). Of note, the slower-migrating OTUB2-inhibition-induced X band was independent of the presence of the GFP tag of GFP-OTUB2, as a similar slower migrating (X′) band was detected with a Flag antibody in cells ectopically expressing Flag-HA tagged OTUB2 (Figure S7B). These results support the hypothesis that OTUB2 inactivation in cells induces OTUB2 modification.

**Figure 4:**
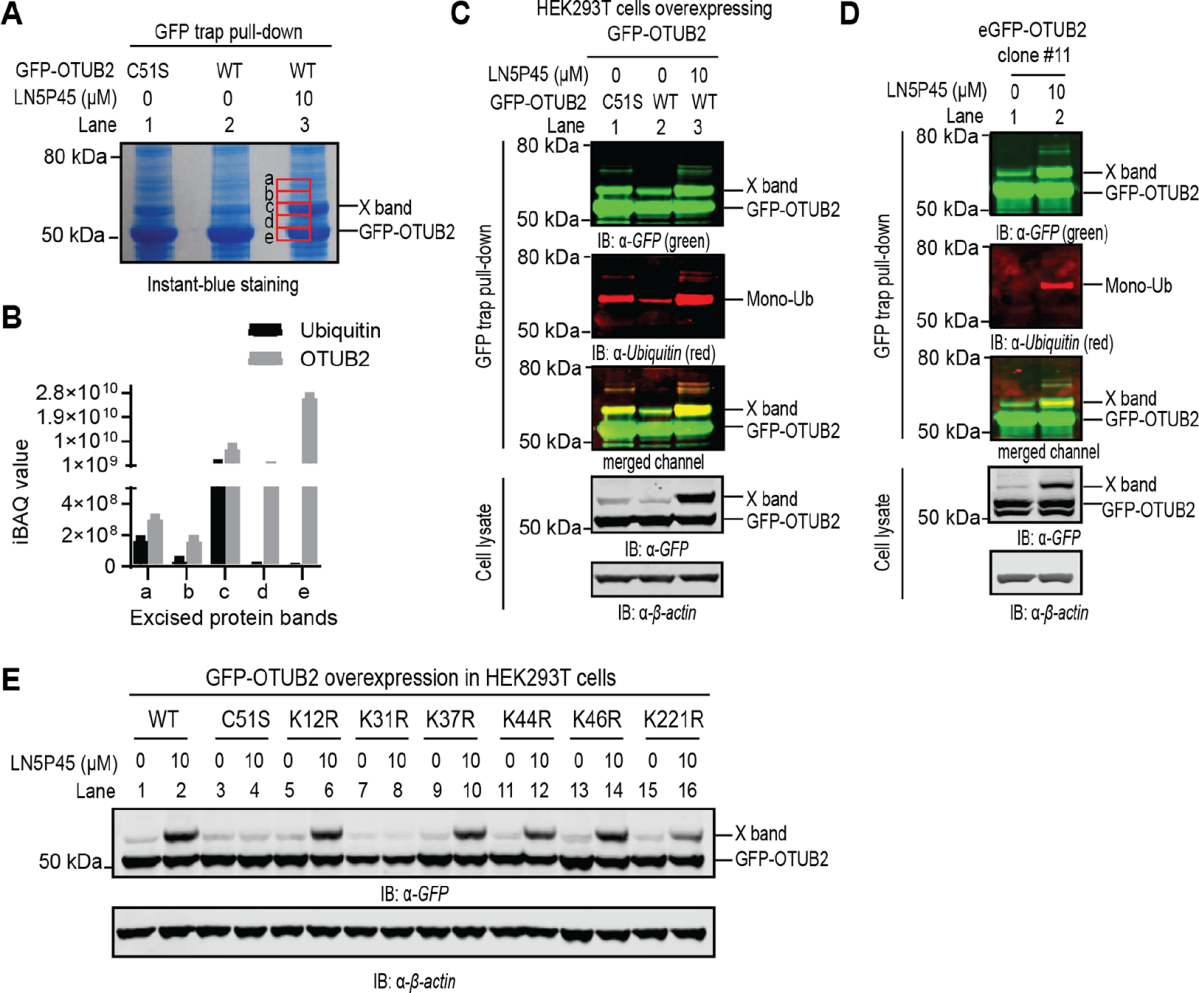
OTUB2 is monoubiquitinated on lysine 31 upon inhibitor treatment. (A) Instant-blue staining of SDS-PAGE of the indicated GFP-trap-pulled-down proteins. GFP-OTUB2 WT or C51S mutant was transfected into HEK293T cells as indicated. 24 h post-transfection, cells were treated with DMSO or 10 μM **LN5P45** for 4 h. GFP-OTUB2 was enriched by GFP trap beads, subjected to SDS-PAGE separation, and bands a–e were excised for mass spectrometry analysis. (B) Label-free quantification of the amount of ubiquitin and OTUB2 in the excised protein bands. (C) Ubiquitination on exogenously expressed GFP-OTUB2 is confirmed by immunoblotting of the GFP-trap enriched proteins with anti-ubiquitin antibody. HEK293T cells were treated as described under (A) and, after SDS-PAGE separation of the enriched proteins, analyzed by immunoblotting. (D) Ubiquitination on endogenously expressed GFP-OTUB2 is confirmed by immunoblotting of the GFP-trap enriched proteins with anti-ubiquitin antibody. Endogenously tagged GFP-OTUB2 HeLa cells (clone #11) were treated with DMSO or 10 μM **LN5P45** for 4 h. eGFP-OTUB2 was enriched by GFP trap beads, subjected to SDS-PAGE separation, and immunoblotting. (E) Lysine to arginine point mutation analysis of OTUB2 identifies lysine 31 as critical for OTUB2 monoubiquitination. The indicated GFP-OTUB2 mutants were exogenously expressed in HEK293T cells, the cells were treated with DMSO or **LN5P45** at 10 μM for 4 h, and cell lysates were immunoblotted with GFP antibody.

To elucidate the nature of the inhibitor-induced modification, we used GFP trap beads and SDS-PAGE to enrich the X band protein from lysates of **LN5P45**-treated HEK293T cells that expressed exogenous GFP-OTUB2 WT. Five protein bands covering the X band area were excised (Figure 4A) and subjected to label-free protein mass spectrometry analysis. The analysis revealed highly abundant ubiquitin peptides at the exact X band location (Figure 4B). This finding was confirmed by anti-ubiquitin immunoblot analysis of the pulled-down GFP-OTUB2 samples (Figure 4C). Based on the molecular weights of the ubiquitin antibody-detected bands, the X band corresponds to monoubiquitinated GFP-OTUB2, while the less abundant high-molecular-weight bands might represent ubiquitinated forms with additional modifications (Figure 4C). Consistent with these data, the X band enriched from lysates of endogenous GFP-OTUB2 expressing HeLa cells was also found to represent monoubiquitinated GFP-OTUB2 (Figure 4D). Interestingly, a low level of monoubiquitination on GFP-OTUB2 was detected in non-treated cells, but OTUB2 inactivation by inhibitor treatment or, to a lesser extent, active site mutation strongly enhanced the monoubiquitination level (Figure 4C, Figure S7A).

We next set out to identify which amino acid(s) is/are ubiquitinated in GFP-OTUB2. Although peptide sequencing by mass spectrometry failed to locate the specific modification site, it helped to exclude several lysines. OTUB2 K12, K31, K37, K44, K46, and K221 were possible sites, and we therefore generated single lysine to arginine mutants of GFP-OTUB2 for each of these sites. Analysis in HEK293T cells showed that only K31R mutation resulted in loss of the monoubiquitination band upon OTUB2 inhibitor treatment and that monoubiquitination of the catalytic inactive mutant C51S was not further increased by the inhibitor either (Figure 4E).

Altogether these data demonstrate that OTUB2 is ubiquitinated *via* lysine 31 and that inactivation of OTUB2 by catalytic site mutation or inhibitor treatment leads to elevated monoubiquitination. This shows how inhibition of OTUB2 stimulates its modification, which may result in secondary effects in cells following OTUB2 inhibition.

## Conclusions

The inhibition of DUB activity is subject to a growing interest in drug development, and several successful examples of potent DUB inhibitors have been published in recent years.^13,14,25^ In this study, we developed the improved covalent OTUB2 inhibitor **LN5P45** through optimization of the original OTUB2-COV1 inhibitor by addressing absolute stereochemistry and aromatic ring substituents. We determined the preferred absolute stereochemistry as (1*S*,2*S*), and the **LN5P45**-OTUB2 co-crystal structure explained the molecular details of the inhibitor binding mode. The co-structure also revealed that the aromatic moiety points outside the protein. For future inhibitor optimizations, it could be worthwhile to introduce more hydrophilic residues at the phenyl ring to accommodate for its solvent-exposed nature. Our substituent analysis revealed that the *meta* position on the aromatic ring is the best site to introduce modifications. This opens the way to install fluorescent/affinity tags or E3 ligase ligands to create OTUB2-specific activity-based probes or PROTACS, respectively. As such, compound **LN5P45** could aid in the development of future therapeutics that target diseases associated with elevated levels and/or increased activity of OTUB2.

Interestingly, OTUB2 is monoubiquitinated upon inhibitor treatment in cells and we were able to pinpoint Lys31 as the ubiquitination site. Ubiquitination of DUBs is a common post-translational modification that can regulate DUB function, localization, or stability, and several examples have been reported.^6^ Monoubiquitination of OTUB1, primarily at lysines 59 and 109, is critical for OTUB1 function to suppress UbcH5 and induce p53,^26^ and the DUB activity of Ataxin-3 and JOSD1 can be enhanced through ubiquitination of lysine 117 on Ataxin-3^27,28^ and lysine 136 on JOSD1.^29^ As opposed to activation, the enzymatic activity of UCHL1 is restricted by monoubiquitination of lysine residues near the active site.^30^ In addition to these activity alterations, the subcellular localization of BAP1 is regulated by multi-monoubiquitination within its NLS region.^31^ USP30 is ubiquitinated by Parkin for proteasome degradation.^32^ USP4 was shown to self-deubiquitinate after being ubiquitinated by E3 ligase Ro52, and it was suggested that USP4 forms a heterodimeric protein complex with Ro52 in which both proteins transregulate each other.^33^ In addition, auto-deubiquitination of USP4 has been shown to stimulate homologous recombination and its interaction with CtIP to recruit CtIP to DNA damage sites, suggesting ubiquitination-dependent regulation of DUB enzyme interaction and function.^34^ However, we observed and report here for the first time the monoubiquitination of a DUB in cells as a result of inhibitor treatment. Whether cells put this mechanism into place to counteract the effect of inhibition and whether it is unique to OTUB2 and/or this particular type of (covalent) inhibitor remain to be investigated but may deliver valuable information for future drug development campaigns. Future research may unveil the potential roles of OTUB2 monoubiquitination in the regulation of its enzymatic activity, localization, degradation, and other functions and could lead to the identification of a similar effect for other DUBs and inhibitors. The selective OTUB2 inhibitor identified and characterized in this study will be a critical lead for such studies.

## Methods

### Chemical and Chemoenzymatic Synthesis of OTUB2 Inhibitors

Synthetic procedures and characterization data for the OTUB2 inhibitors are provided in the Supporting Information.

### Ubiquitin Substrate *In Vitro* Cleavage Assays

An overview of the used recombinant DUBs is provided in Table S1. The assays were performed in “nonbinding surface flat bottom low flange” black 384-well plates (Corning 3820) at room temperature in a buffer containing 50 mM Tris·HCl, 100 mM NaCl, pH 7.6, 2.0 mM cysteine, 1 mg/mL 3-[(3-cholamidopropyl)-dimethylammonio]propanesulfonic acid (CHAPS), and 0.5 mg/mL γ-globulins from bovine blood (BGG). All dispensing steps involving buffered solutions were performed on a Biotek MultiFlowFX dispenser. Each well had a final volume of 20.4 μL. The compounds were dissolved in 10 mM and 1 mM DMSO stock solutions, and appropriate volumes were transferred to the empty plates using a Labcyte Echo acoustic dispenser to obtain a 12-point serial dilution. A DMSO back-fill was performed to obtain equal volumes of DMSO (400 nL) in each well. *N*-Ethylmaleimide (NEM, 10 mM) was used as a positive control (100% inhibition) and DMSO as a negative control (0% inhibition). A 10 μL portion of buffer was added, and the plate was vigorously shaken for 20 s. Next, 7 μL of the DUB was added to reach the following final concentrations: OTUB1, 50 nM; OTUB2, 25 nM; OTUD1, 10 nM; OTUD2, 50 nM; OTUD3, 5 nM; OTUD6B, 10 nM; Cezanne, 5 nM; UCHL1, 1 nM; USP16, 2 nM, followed by incubation for 120 min. A 3 μL portion of the substrate (UbRhoMP, or K48-linked diUb FRET pair^35^ for OTUB1) was added (final concentration 500 nM), and the increase in fluorescence over time was recorded using a BMG Labtech PHERAstar plate reader (excitation 487 nm, emission 535 nm). The initial enzyme velocities were calculated from the slopes, normalized to the positive and negative controls, and plotted against the inhibitor concentrations using the built-in equation “[inhibitor] vs response – variable slope (four parameters), least-squares fit” with constraints “Bottom = 0” and “Top = 100” in GraphPad Prism 8 software to obtain the IC_50_ values.

In the case of the *k*_inact_/*K*_I_ determinations, the order of OTUB2 and substrate addition was reversed and no incubation time was used. All data fitting and calculations were done using GraphPad Prism 8 software. The fluorescence intensities were plotted against time (in seconds) after a baseline correction using the DMSO control for each inhibitor concentration. The data were fitted to the equation FI = (*v*_i_/*k*_obs_)[1 – e^–*k*(obs)*t*^]. The thus obtained *k*_obs_ values were plotted against the concentration inhibitor, fitted to a linear regression, and the *k*_inact_/*K*_I_ value was determined from the slope.

### Covalent Complex Formation Mass Spectrometry Analysis

Samples of 1.4 μM OTUB2 in 70 μL of buffer containing 50 mM Tris·HCl, 100 mM NaCl at pH 7.6, and 2.0 mM cysteine were prepared. These samples were treated with 1 μL of DMSO or 1 μL of a 10 mM **LN5P45** stock solution in DMSO (140 μM final concentration) and incubated for 30 min at room temperature. Samples were then diluted 3-fold with water and analyzed by mass spectrometry by injecting 1 μL into a Waters XEVO-G2 XS Q-TOF mass spectrometer equipped with an electrospray ion source in positive mode (capillary voltage 1.2 kV, desolvation gas flow 900 L/h, *T* = 60 °C) with a resolution of *R* = 26,000. Samples were run using two mobile phases: (A) 0.1% formic acid in water and (B) 0.1% formic acid in CH_3_CN on a Waters Acquity UPLC protein BEH C4 column [300 Å, 1.7 μm (2.1 × 50 mm^2^), flow rate = 0.5 mL/min, run time = 14.00 min, column *T* = 60 °C, and mass detection 200–2500 Da]. Gradient: 2–100% B. Data processing was performed using Waters MassLynx mass spectrometry software 4.1, and ion peaks were deconvoluted using the built-in MaxEnt1 function.

### OTUB2 Crystallization

For crystallization, the covalent **LN5P45**-OTUB2 complex was generated by incubating 40 μM purified OTUB2 with a five-fold excess of the inhibitor for 1 h at 37 °C in GF buffer (50 mM Tris pH 7.5, 100 mM NaCl, 2 mM TCEP). The formation of the covalent complex was confirmed using LCMS (Xevo) before being applied to a Superdex75 16/60 (GE Healthcare) gel filtration column equilibrated in GF buffer. The peak fractions containing the complex were concentrated to 24.5 mg/mL and flash frozen in liquid N_2_, awaiting crystallization.

Sitting drops were set up in a 1:1 ratio with the mother liquor, and crystals appeared after several days in conditions with 0.1 M HEPES pH 8.0, 8–20% (v/v) isopropanol and 10–16% PEG 4000. Single crystals were frozen in mother liquor and 30% ethylene glycol as a cryoprotectant and shipped to Diamond Light Source (DLS, United Kingdom) for diffraction.

### Crystal Data Collection and Structure Determination

Crystals were diffracted at beamline I04-1 at Diamond, and the resulting data sets were processed with DIALS^36^ using the DLS computing grid. The CCP4 program suite^37^ was used: first data reduction with Aimless,^38^ followed by molecular replacement using PHASER^39^ with OTUB2 from PDB entry 5QIO as a search model. Iterative cycles of refinement using REFMAC^40^ and model building with COOT^41^ were performed before placing the **LN5P45** inhibitor in the remaining density. Restraints for the inhibitor were generated using the GRADE webserver (Global Phasing Ltd.) and used in further refinement. Data processing and refinement statistics are reported in Table S2, and the final model is deposited in the Protein Data Bank (PDB ID: 8CMS).

### Cell Culture and Reagents

HEK293T (Cat# ATCC CRL-3216), HeLa (ATCC), and bone metastatic (BM) MDA-MB-231 cells^42^ (laboratory of Peter ten Dijke) were cultured under standard conditions in Dulbecco’s modified Eagle’s medium (DMEM) (Gibco) supplemented with 8% FCS (Biowest) and 1% penicillin/streptomycin at 37 °C and 5% CO_2_. All cell lines were tested for mycoplasma contamination using a MycoAlert Mycoplasma Detection Kit (Lonza, Catalog #: LT07-318) on a monthly basis.

For siRNA transfections, OTUB2 siRNA oligos were purchased from Dharmacon. The siOTUB2 pool consists of four individual siRNA’s, including (siGENOME Cat# MQ-010983-01-0002), siOTUB2_P1 (Cat# D-010983-01), siOTUB2_P2 (Cat# D-010983-02), siOTUB2_P3 (Cat# D-010983-03), and siOTUB2_P4 (Cat# D-010983-04). Silencing was performed in BM MDA-MB-231 cells as follows: for a six-well plate format, 200 μL of siRNA (500 nM stock) was incubated with 4 μL of Dharmafect reagent 1 (Dharmacon) diluted in 196 μL of medium without supplements (total volume of 200 μL of transfection mix) with gentle shaking for 20 min at room temperature (RT). A total of 80,000 cells resuspended in 1.6 mL of growth medium were added to transfection mixes to a total volume of 2 mL per well and cultured for 3 days prior to further analysis.

For DNA transfections, HEK293T cells were seeded to achieve 50–60% confluence the following day and transfected using PEI (polyethylenimine, Polysciences Inc., Cat# 23966) as follows: 200 μL of DMEM medium without supplements was mixed with DNA and PEI (1 mg/mL) with a ratio at 1:3 (e.g., 1 μg DNA:3 μL PEI), incubated at RT for 20 min, and added drop-wise to the cells for culturing for 24 h prior to further analysis. The reaction was scaled using the same component ratios as follows: 12-well plate–1 μg DNA, 6-well plate–3 μg DNA, 6 cm dish–8 μg DNA.

### DNA Constructs

GFP-OTUB2 WT and C51S mutant,^21^ HA-Ub,^43^ Flag-HA-OTUB2 WT (#22552, Addgene),^44^ and pOPINK-OTUB2 (#61421, Addgene)^45^ constructs have been previously described.

For site-directed mutagenesis of OTUB2 K12R, K31R, K37R, K44R, K46R, and K211R, the same protocol was used as before.^46^ In brief, a PCR mixture containing a GFP-OTUB2 WT template, mutation primers, Pfu buffer, dNTPs, Turbo Pfu Polymerase (Agilent), and MilliQ water up to 50 μL reaction volume was subjected to PCR amplification using the following program: 95 °C for 2 min (95 °C for 50 s; 60 °C for 1 min; 68 °C for 1 min/Kb) × 18 cycles; 68 °C for 20 min; 4 °C forever. PCR products were digested with 1 μL DpnI (Thermo Fischer Scientific) for 2 h at 37 °C to remove the methylated DNA template and then transformed into competent DH5α. All mutated constructs were verified by sequencing. For primer sequences, refer to Table S3. All primers were purchased from IDT.

### Antibodies

The following antibodies were used for detection of endogenous and overexpressed proteins by immunoblots: mouse anti-Ubiquitin (P4D1, Santa Cruz Biotechnology, 1:1000 dilution), mouse anti-HA (16B12, Enzo Lifesciences, 1:1000 dilution), rabbit anti-mGFP^47^ (self-made, 1:1000 dilution), mouse anti-GAPDH (1D4, Enzo Lifesciences, 1:1000 dilution), or mouse anti-β-actin (clone AC-15, Sigma-Aldrich, 1:5000 dilution).

### Generation of Endogenously Tagged Cell Lines

Endogenous tagging of OTUB2 was performed as described previously.^23^ In brief, HeLa cells were co-transfected with OTUB2/pX330 and OTUB2/endoGFP plasmids. Transfected cells were single-cell sorted by flow cytometry and validated by OTUB2 siRNA knockdown, DUB probe labeling, and immunoblots.

OTUB2/pX330 plasmid was generated by cloning OTUB2 5′ end-targeting guide sequence (gtcactatggtcagtgatcg) into pX330-U6-Chimeric_BB-CBh-hSpCas9 plasmid. OTUB2/endoGFP plasmid was created by modifying the GFP-C1 vector, where the OTUB2 left homology arm (agggggaggcatcctatcaaatggaccctaacgcataggctgaaggcacgtgaagtcccacccactttctatgacgtcccgcgtcccggcttctgattgcccgcctgagacgtcaatcgcagggcgtgtgtcttgctgggacacagtggaggtctaacctttggtttgcggagcggtcgggtgtattctccgccgcccccacgccctcgaggtccccgccaccgaaccagcggcggagcccgcccgcgcctcccgcggcattcccgcaccggatcgctcctcgctggggcgggacctggcctggcggctctggtcactatg) was inserted using NdeI/NheI sites (replacing the CMV promoter) and the right homology arm (gtcagtgatcgtgggggatcgcgaagggggagcgggcagggggcgcggtgggcggggtcgctgccggagcgggtgcacccgcgggacgggggtcggacgcgaggctcagcccccagctcgcccccgccgctttccgaccccctgaaatacggagtccggacggatactgagggccaagtcgcgcccccctgtaccccgtggatgtgcagctgaggaggtccagctcggccccgagcccccgcccccagcgtgtccgccccaggtggcccggggcgccgcctcgacggcgctgggtgggcgccctcgacggaagcagggacaggga) using EcoRI/BamHI sites.

### Real-Time Quantitative Polymerase Chain Reaction (RT-qPCR)

The mRNA level change of endogenous OTUB2 was assessed by RT-qPCR. Total RNAs were extracted using the NucleoSpin RNA II kit (MACHEREY-NAGEL) following the manufacturer instructions. Total RNA (1 μg) was reversely transcribed using RevertAid First Stand cDNA Synthesis Kits (Thermo Fisher) to synthesize cDNA, and real-time quantitative PCR experiments were performed using SYBR Green (Promega) in a CFX connect Real-Time PCR detection system (Bio-Rad). All the values for target gene expression were normalized to GAPDH. Primers used for RT-qPCR are as follows: OTUB2 forward primer TTCTTCGGGACCATCCTGAAA and reverse primer CCAGGTAGGAATAGCCCAAGG, GAPDH Forward primer TGCACCACCAAC TGCTTAGC and reverse primer CTCATGACCACAGTCCATGC. All the experiments were repeated in *n* = 3 independent experiments.

### Ub-Based Activity-Based Probe (Rhodamine-Ub-PA) Labeling

The in-cell inhibition of OTUB2 was assessed by using a DUB probe labeling assay.^15^ In brief, cell pellets were resuspended in HR buffer (50 mM Tris–HCl, 5 mM MgCl_2_, 250 mM sucrose, 2 mM TCEP, and a Protease inhibitor tablet (Roche), pH 7.4) and lysed by sonication (Bioruptor, Diagenode, high intensity for 10 min with an ON/OFF cycle of 30 s) at 4 °C. Clarified cell lysate (40 μg) was labeled with a Rhodamine-Ubiquitin-propargylamide probe (final concentration at 1 μM) at 37 °C for 30 min. Reactions were stopped by the addition of LDS (lithium dodecyl sulfate) sample buffer (Invitrogen Life Technologies, Carlsbad, CA, USA) containing 2.5% β-mercaptoethanol, followed by boiling for 7 min.

### SDS-PAGE, In-Gel Fluorescence Scan, and Immunoblotting

Samples were resolved on precast Bis-Tris NuPAGE Gels (Invitrogen, including 4–12%, and 10% for different samples) using MOPS buffer (Invitrogen Life Technologies, Carlsbad, CA, USA). For fluorescence scan, labeled enzymes were visualized by in-gel fluorescence using a Typhoon FLA 9500 imaging system (GE Healthcare Life Sciences) (Rhodamine channel for probe, Cy5 channel for protein marker). For immunoblotting, proteins were transferred to a nitrocellulose membrane (Protan BA85, 0.45 μm, GE Healthcare) at 300 mA for 3 h. The membranes were blocked in 5% milk (skim milk powder, LP0031, Oxiod) in 1× PBS (P1379, Sigma-Aldrich), incubated with a primary antibody diluted in 5% milk in 0.1% PBS-Tween 20 (PBST) overnight at cold room, washed three times for 5 min in 0.1% PBST, incubated with the secondary antibody diluted in 5% milk in 0.1% PBST for 1 h, and washed three times again in 0.1% PBST. The signal was visualized using a LICOR Odyssey system. The intensity of bands was quantified using Image Studio software.

### GFP-OTUB2 Pull-Down Assays

To check the PTM on GFP-OTUB2, GFP trap pulldown assays were applied as before.^46^ Harvested cell pellets were lysed in 300 μL of lysis buffer 1 (50 mM Tris–HCl, pH 7.5, 150 mM NaCl, 0.5% Triton X-100, 10 mM *N*-methyl maleimide (general DUB inhibitor diluted in DMSO, freshly added), and protease inhibitors (Roche Diagnostics, EDTA-free, freshly added)). Then, 100 μL of lysis buffer 2 (100 mM Tris–HCl, pH 8.0, 1 mM EDTA, and 2% SDS) was added to the crude lysates; samples were sonicated (Fisher Scientific FB120 Sonic Dismembrator, three pulses, amplitude 40%); and SDS was subsequently diluted by bringing the sample volume to 1 mL with lysis buffer 1. After centrifugation (20 min, 4 °C, 20,817*g*), lysates were incubated with 6 μL of GFP Trap Agarose (Chromotek) overnight at 4 °C. Beads were washed five times with lysis buffer 1 and denatured with sample buffer by heating at 95 °C for 7 min. Samples were subjected to 4–12% SDS-PAGE, and gel samples were either sliced for protein mass spectrometry detection or transferred to Nitrocellulose membranes for immunoblots, as indicated.

### Protein Mass Spectrometry of Gel Slices

For MS analysis, gel slices were subjected to reduction with dithiothreitol, alkylation with iodoacetamide, and in-gel trypsin digestion using a Proteineer DP digestion robot (Bruker). Tryptic peptides were extracted from the gel slices, lyophilized, dissolved in 95/3/0.1 v/v/v water/acetonitrile/formic acid, and subsequently analyzed by on-line C18 nanoHPLC MS/MS with a system consisting of an Easy nLC 1000 gradient HPLC system (Thermo, Bremen, Germany) and a LUMOS mass spectrometer (Thermo). Samples were injected onto a homemade precolumn (100 μm × 15 mm; Reprosil-Pur C18-AQ 3 μm, Dr. Maisch, Ammerbuch, Germany) and eluted *via* a homemade analytical nano-HPLC column (30 cm × 50 μm; Reprosil-Pur C18-AQ 3 um). The gradient was run from 10% to 40% solvent B (20/80/0.1 water/acetonitrile/formic acid (FA) v/v) in 30 min. The nano-HPLC column was drawn to a tip of ∼5 μm and acted as the electrospray needle of the MS source. The LUMOS mass spectrometer was operated in data-dependent MS/MS mode for a cycle time of 3 s, with a HCD collision energy at 32 V, and the MS2 spectrum was recorded in the orbitrap. In the master scan (MS1), the resolution was 120,000 and the scan range was 400–1500 at an AGC target of 400,000 at a maximum fill time of 50 ms. Dynamic exclusion after *n* = 1 with an exclusion duration of 10 s. Charge states 2–5 were included. MS2 precursors were isolated with the quadrupole with an isolation width of 1.2 Da. The first mass was set to 110 Da. The MS2 scan resolution was 30,000 with an AGC target of 50,000 at a maximum fill time of 60 ms.

### Profiling of **LN5P45**-Reactive Cysteines by SLC-ABPP

SLC-ABPP profiling was performed as described before.^24^ In short, HeLa cells, transfected with GFP or GFP-OTUB2 WT with PEI reagent for 24 h, were grown to 80% confluence and incubated with either DMSO or 10 μM **LN5P45** for 4 h in complete medium. Cells were harvested, lysed by sonication in ice-cold PBS, and centrifuged at 15,000 RPM for 2 min to remove cell debris. Protein concentrations were then determined by a BCA Gold protein assay. Proteomes were normalized to 1 mg/mL in 100 μL for each sample.

Each DMSO- and **LN5P45**-treated proteome was labeled with the 500 μM DBIA probe for 1 h in the dark at room temperature (RT). Excess DBIA, along with disulfide bonds, was quenched and reduced using 5 mM dithiothreitol for 30 min in the dark at RT. Reduced disulfide bonds were alkylated using 20 mM iodoacetamide for 30 min in the dark at RT. Proteins were precipitated using chloroform/methanol, re-solubilized in 40 mM HEPES pH 8.4, and digested using TPCK-treated trypsin and endoLysC (1:12.5 enzyme/protein ratio) overnight at 37 °C. Digested peptides were labeled using TMTpro16-plex reagents in a 1:4 ratio by mass (peptides:TMT reagents) for 1 h at RT. Excess TMT reagent was quenched with 5 μL of 6% hydroxylamine for 15 min at RT. Next, samples were mixed 1:1 across all TMT channels, and the pooled sample was dried using a Speedvac.

Dried samples were reconstituted in 1 mL of PBS and enriched by Pierce streptavidin magnetic beads (Catalog number: 88816, Thermo Scientific) by rotating end-over-end overnight at 4 °C. Nonspecific binding peptides were washed using the following procedure: 3 × 1 mL of PBS pH 7.4, 2 × 1 mL of PBS with 0.1% SDS pH 7.4, and 3 × 1 mL of HPLC-grade water. DBIA probe-containing peptides were eluted using 700 μL of 50% acetonitrile with 0.1% TFA, dried using a Speedvac, and desalted using a mini-SPE column (10% sorbent material of a Waters, OASIS 1 cc HLB 30 mg cartridge). The column was washed with 200 μL of 90% acetonitrile and 3× 200 μL of 10 mM NH_4_HCO_3_ pH 8.4. Dried samples were dissolved in 200 μL of 10 mM NH_4_HCO_3_ pH 8.4, loaded onto the column, washed 3× with 200 μL of 10 mM NH_4_HCO_3_ pH 8.4, and eluted into three fractions with 150 μL each of 10, 20, and 50% acetonitrile (in 10 mM NH_4_HCO_3_). Samples were re-dried using a Speedvac.

### Mass Spectrometry of SLC-ABPP

Peptides were lyophilized, dissolved in 0.1% formic acid, and subsequently analyzed by on-line C18 nanoHPLC MS/MS with a system consisting of an Ultimate3000nano gradient HPLC system (Thermo, Bremen, Germany) and an Exploris480 mass spectrometer (Thermo). Fractions were injected onto a cartridge precolumn (300 μm × 5 mm, C18 PepMap, 5 μm, 100 A) and eluted *via* a homemade analytical nano-HPLC column (50 cm × 75 μm; Reprosil-Pur C18-AQ 1.9 μm, 120 A, Dr. Maisch, Ammerbuch, Germany). The gradient was run from 2 to 40% solvent B (20/80/0.1 water/acetonitrile/formic acid (FA) v/v) in 120 min. The nano-HPLC column was drawn to a tip of ∼10 μm and acted as the electrospray needle of the MS source. The mass spectrometer was operated in data-dependent MS/MS mode for a cycle time of 3 s, with a HCD collision energy at 36 V, and the MS2 spectrum was recorded in the orbitrap, with a quadrupole isolation width of 1.2 Da. In the master scan (MS1), the resolution was 120,000 and the scan range was 350–1600 at a standard AGC target at a maximum fill time of 50 ms. A lock mass correction on the background ion *m*/*z* = 445.12 was used. Precursors were dynamically excluded after *n* = 1 with an exclusion duration of 45 s and with a precursor range of 20 ppm. Charge states 2–5 were included. For MS2, the first mass was set to 110 Da, and the MS2 scan resolution was 45,000 at an AGC target of 200% with a maximum fill time set to auto.

### Protein Mass Spectrometry Data Analysis

In a post-analysis process, raw data were first converted to peak lists using Proteome Discoverer version 2.4 (Thermo Electron) and then submitted to the Uniprot *Homo sapiens* minimal database (20,205 entries) using Mascot v. 2.2.04 (www.matrixscience.com) for protein identification. Mascot searches were done with 10 ppm and 0.02 Da deviation for precursor and fragment mass, respectively, and the enzyme trypsin was specified. Up to two missed cleavages were allowed for gel slices and three missed cleavages for SLC-ABPP. For gel slices, methionine oxidation and ubiquitination (GG) on lysine were set as a variable modification; carbamidomethyl on Cys was set as a fixed modification. For SLC-ABPP, methionine oxidation, Acetyl (protein N-term) and DBIA on Cys were set as a variable modification. Carbamidomethyl on Cys and TMTpro on Lys and N-term was set as a fixed modification. Peptides with an FDR < 1% were accepted. Abundance ratios were calculated by dividing the inhibitor-treated channels with the DMSO-treated channels.

## Supporting Information Available

Synthetic procedures, characterization of compounds, supplementary figures (biochemical inhibition data, IC_50_ determination on nine DUBs, LC-MS analysis of OTUB2-**LN5P45** complex, electron density map of **LN5P45**, gel-based competition assay in MDA-MB-231 cells, **LN5P45** engages endogenous OTUB2 in HeLa cells, and OTUB2 X-band mobility shift with **LN5P45** treatment), schemes (general syntheses of OTUB2 inhibitors and enantiopure cyclopropanes), tables (overview of the purified recombinant human DUBs, X-ray data processing and refinement statistics, and primer sequences for site-directed mutagenesis), and NMR and LC-MS spectra of synthesized compounds (PDF)
Compound checklist (XLSX)

## Accession Codes

The mass spectrometry proteomics data have been deposited to the ProteomeXchange Consortium *via* the PRIDE partner repository with the data set identifiers PXD041346.

Coordinates and structure factors have been deposited with the Protein Data Bank under accession number 8CMS.
