# Supplementary material for: Cellular Validation of a Chemically Improved Inhibitor Identifies Monoubiquitination on OTUB2
Source: ACS Chem Biol. 2023 Aug 29;18(9):2003–13. doi: 10.1021/acschembio.3c00227 (PMC10510154; doi:10.1021/acschembio.3c00227)
Supplement: Supplementary file 1 — cb3c00227_si_001.pdf [file cb3c00227_si_001.pdf]

SUPPORTING INFORMATION

Cellular Validation of Chemically Improved Inhibitor Identifies Monoubiquitination on OTUB2

Jin Gan[1], Jelle de Vries[1], Jimmy J. L. L. Akkermans[2], Yassene Mohammed[3], Rayman T. N. Tjokrodirijo[3], Arnoud H. de Ru[3], Robbert Q. Kim[1], David A. Vargas[4,5], Vito Pol[1], Rudi Fasan[6], Peter A. van Veelen[3], Jacques Neefjes[2], Hans van Dam[1], Huib Ovaa[1†], Aysegul Sapmaz[1*], and Paul P. Geurink[1*]

1 Department of Cell and Chemical Biology, Division of Chemical Biology and Drug Discovery, Leiden University Medical Center, Einthovenweg 20, 2333 ZC, Leiden, The Netherlands.

2 Department of Cell and Chemical Biology and Oncode Institute, Leiden University Medical Center LUMC, Einthovenweg 20, 2333 ZC, Leiden, The Netherlands.

3 Center for Proteomics and Metabolomics, Leiden University Medical Center, Albinusdreef 2, 2333 ZC Leiden, The Netherlands.

4 Department of Chemistry, University of Rochester, Hutchison Hall, 120 Trustee Rd, Rochester, NY, 14627, USA

5 Current affiliation: Process Research and Development, Merck & Co., Inc., Rahway, NJ, 07065, USA.

6 Department of Chemistry & Biochemistry, University of Texas at Dallas, 800 W. Campbell Road, Richardson, TX 75080, USA

*Corresponding authors: A.S. (a.sapmaz@lumc.nl); P.P.G.(p.p.geurink@lumc.nl)

† Deceased

Contents

Supplementary Schemes, Figures and Tables	Page
Scheme S1. General synthesis of OTUB2 inhibitors	S3
Scheme S2. General synthesis of enantiopure cyclopropanes	S4
Figure S1. Biochemical inhibition data	S5, S6
Figure S2. IC_{50} determination on nine DUBs	S7
Figure S3. LC-MS analysis of OTUB2-LN5P45 complex	S8
Figure S4. Electron density map of LN5P45	S9
Figure S5. Gel-based competition assay in MDA-MB-231 cells	S10
Figure S6. LN5P45 engages endogenous OTUB2 in HeLa cells	S11
Figure S7. OTUB2 X-band mobility shift with LN5P45 treatment	S12
Table S1. Overview of the purified recombinant human DUBs	S13
Table S2. X-ray data processing and refinement statistics	S14
Table S3. Primers sequences for site-directed mutagenesis	S15

Supporting Materials and Methods

Chemical synthesis of OTUB2 inhibitors

General	S16
General Synthetic Procedures	S17
Compound Characterization Data	S18
**Supplementary References**	S24
**NMR and LC-MS spectra of synthesized compounds**	S25

Supporting Schemes, Figures and Tables

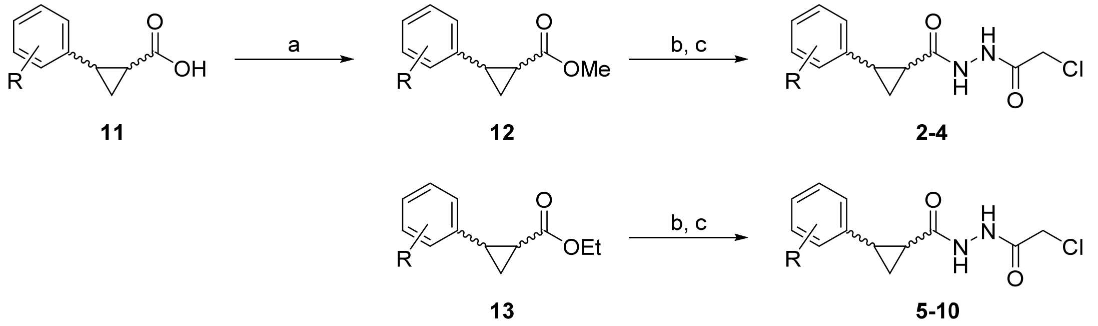

Scheme S1. General synthesis of arylcyclopropyl chloroacethydrazide OTUB2 inhibitors. Stereochemistry and R substituents are defined as shown in Table 1. Reagents and conditions: (a) 2M TMS-diazomethane in Et_2O; (b) hydrazine hydrate, MeOH, 60 °C; (c) chloroacetylchloride, Et_3N, DCM.

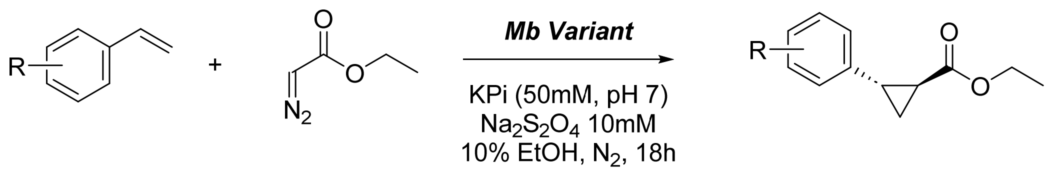

Scheme S2. General synthesis of enantiopure *trans* cyclopropanes via biocatalytic carbene transfer. Reaction conditions: Whole cells expressing Mb variant OD_{600}=20, 10 mM olefin, 30 mM α-ethyl diazoacetate (added slowly over 3 hours), 10 mM $Na_2S_2O_4$, 10% EtOH co-solvent, under N_2, 18 h. All 1*S*,2*S* compounds (**6-10**) were synthesized using Mb(H64V,V68A) as catalyst.[1] Compound **5** with 1*R*,2*R* configuration was synthesized using Mb(RR5) as catalyst.[3]

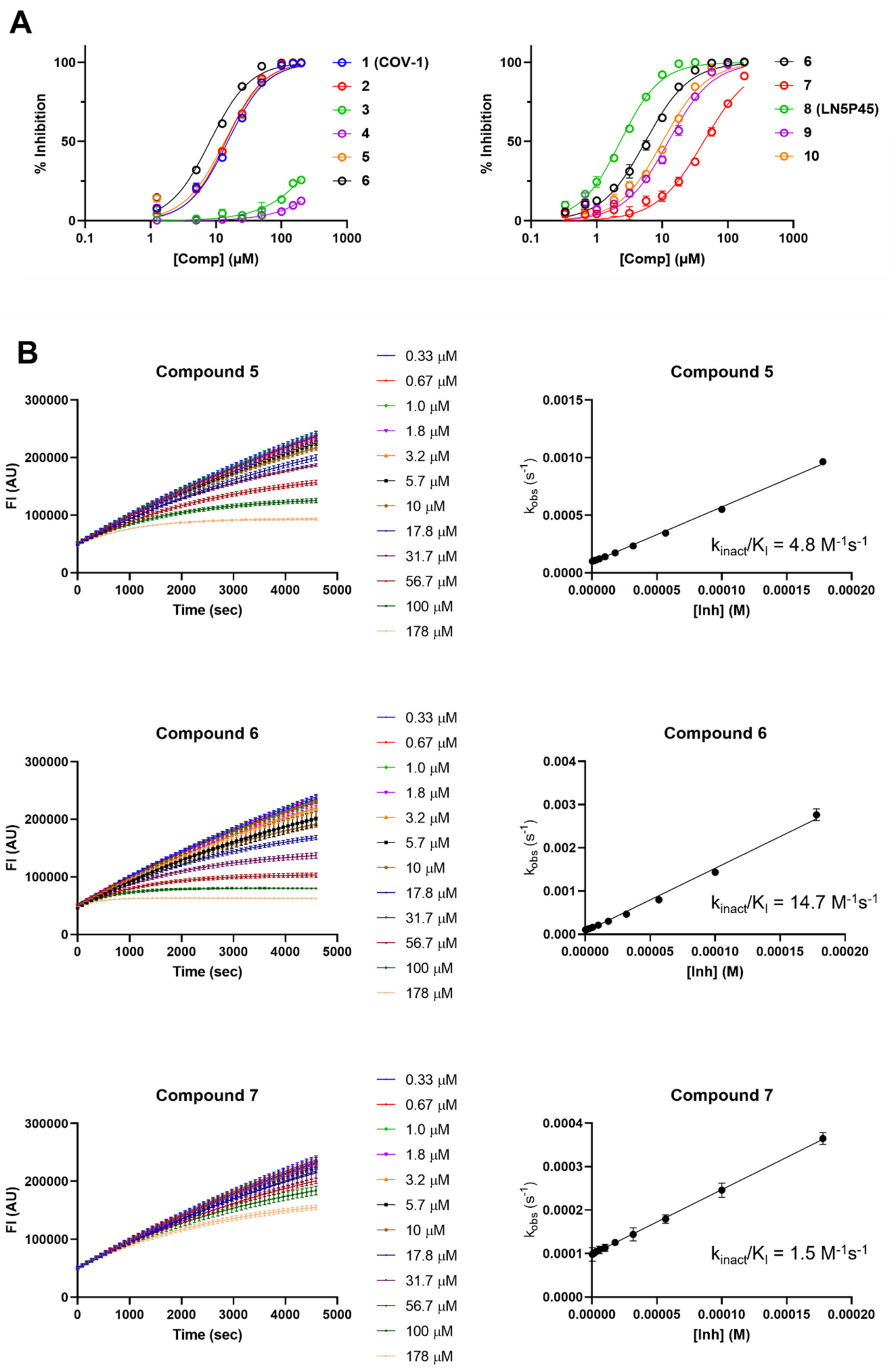
A
% Inhibition
100
50
0
0.1
1
10
100
1000
[Comp] (μM)
1 (COV-1)
2
3
4
5
6
7
8 (LN5P45)
9
10
B
Compound 5
Compound 6
Compound 7
FI (AU)
Time (sec)
0.33 μM
0.67 μM
1.0 μM
1.8 μM
3.2 μM
5.7 μM
10 μM
17.8 μM
31.7 μM
56.7 μM
100 μM
178 μM
kobs (s-1)
[Inh] (M)
kinact/KI = 4.8 M-1s-1
kinact/KI = 14.7 M-1s-1
kinact/KI = 1.5 M-1s-1

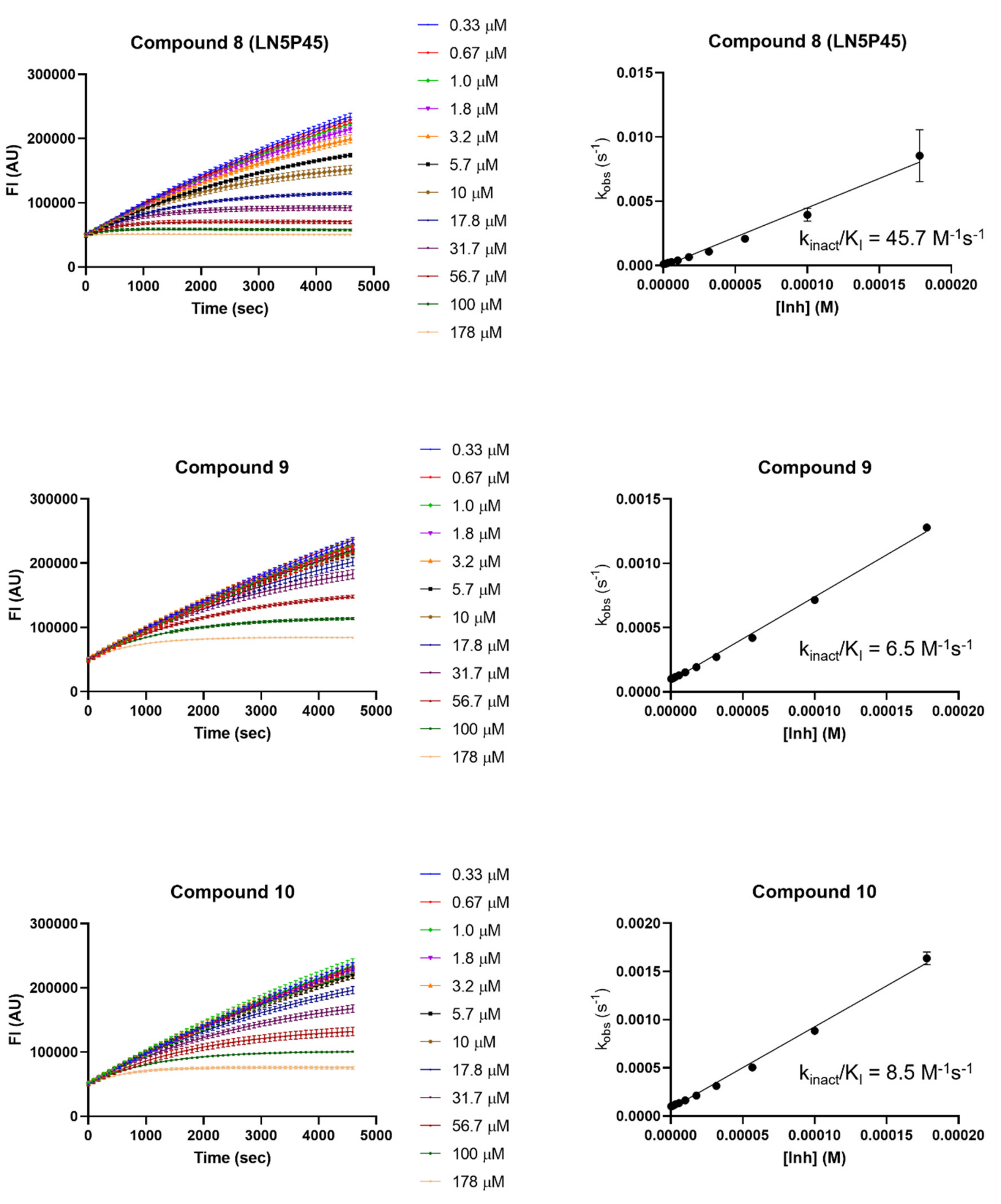

Figure S1. Biochemical inhibition data linked to inhibition values in Table 1. (A) IC_{50} curves for compounds **1-10** after 2h incubation. (B) Determination of the k_{inact}/K_I values for compounds **5-10**. Activity curves on the left and k_{obs} vs inhibitor concentration on the right.

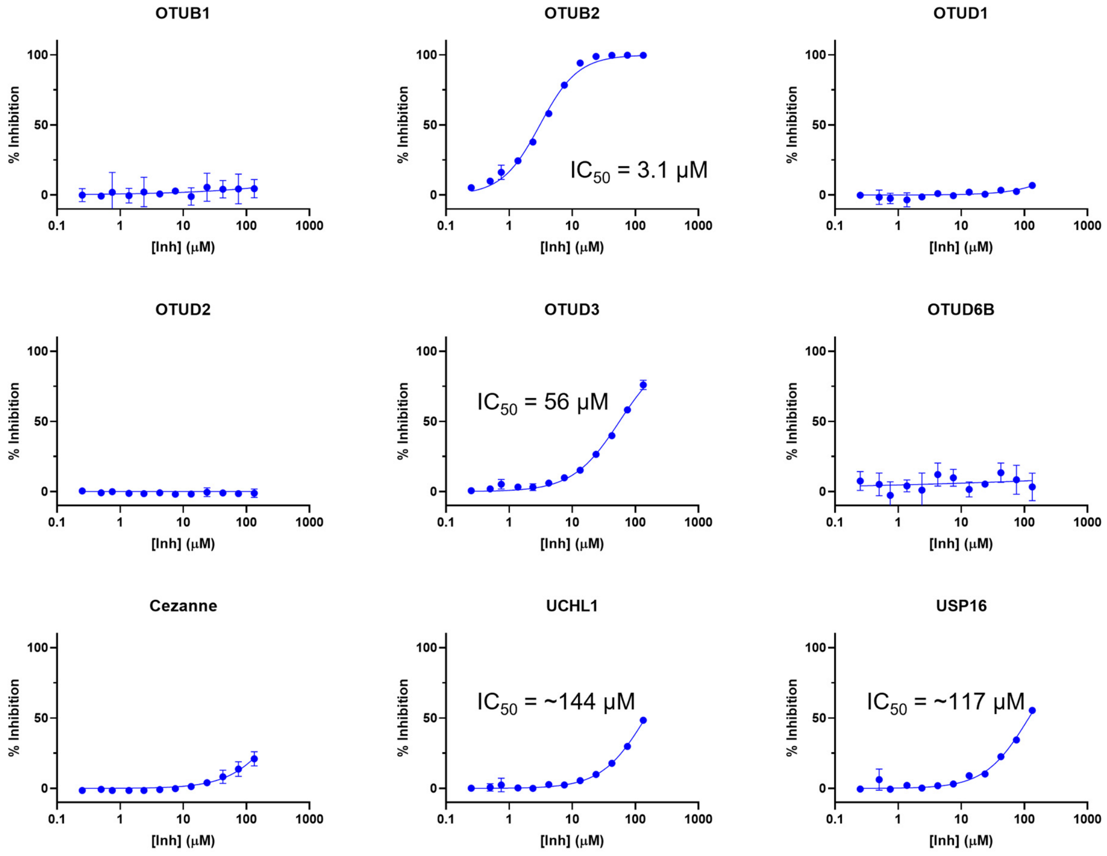

Figure S2. IC_{50} determination of compound **8** (LN5P45) on nine DUBs.

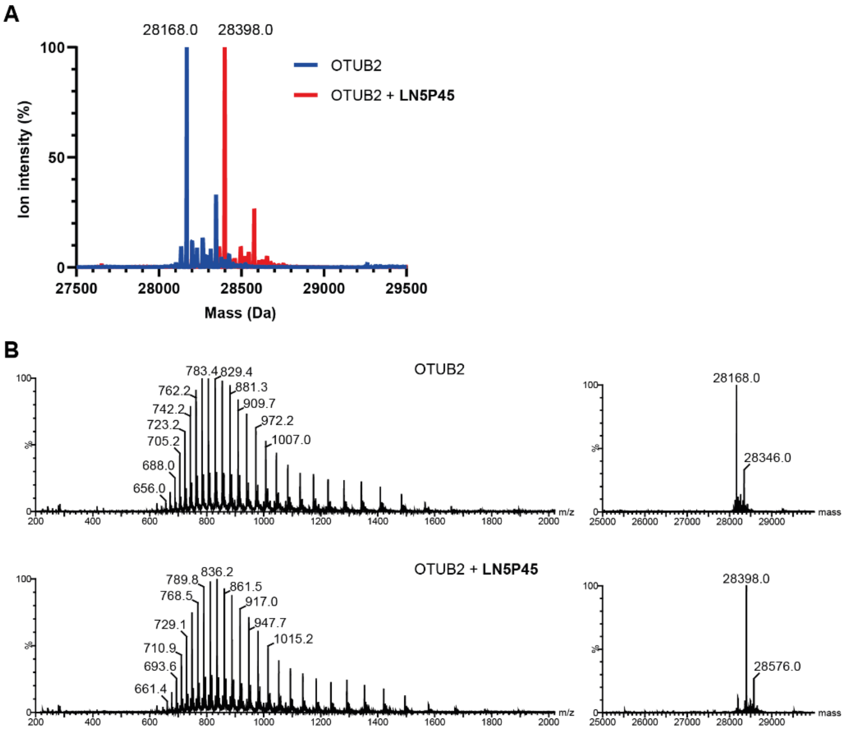

Figure S3. LC-MS analysis confirms formation of a covalent complex of OTUB2 with one molecule LN5P45. (A) Overlay of deconvoluted mass spectra of OTUB2 before (blue) and after (red) reaction with LN5P45. (B) Ion traces (left) and deconvoluted mass (right) of OTUB2 before (top) and after (bottom) reaction with LN5P45. The mass difference of 230 Da corresponds to a substitution reaction at the chloride.

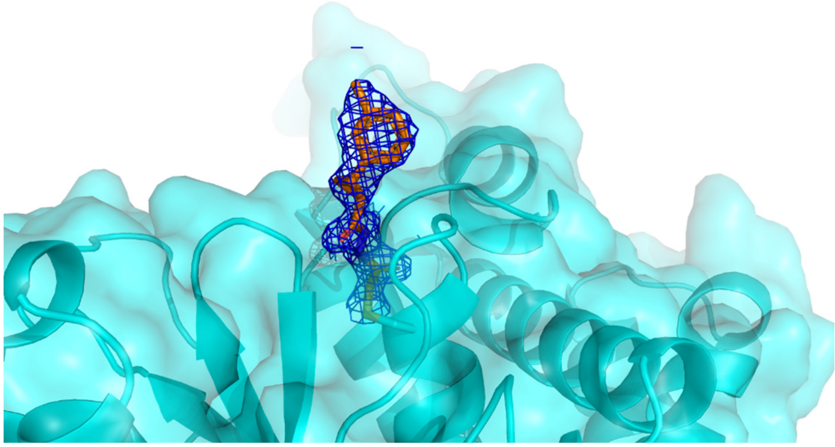

Figure S4. Electron density map of LN5P45.

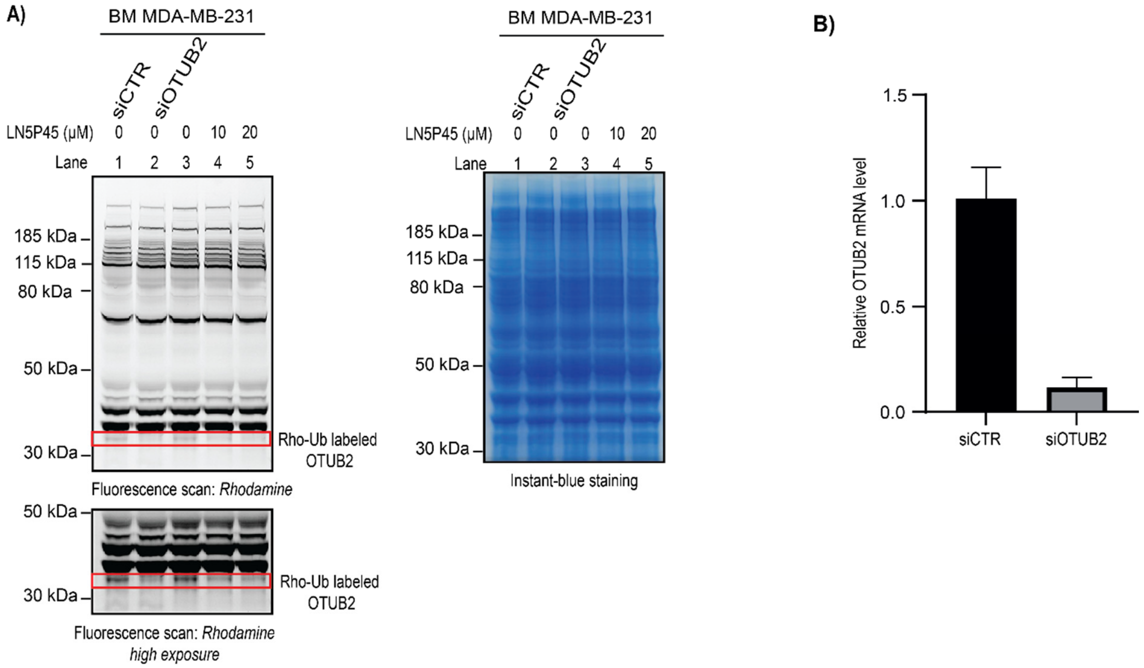

Figure S5. Gel-based competition assay of LN5P45 with Rho-Ub-PA probe. (A) Gel-based Fluorescence labeling of endogenous OTUB2 in bone-metastatic (BM) MDA-MB-231 cells (left panel) and instant-blue stained loading control (right Panel). BM MDA-MB-231 cells were treated with the indicated concentrations of LN5P45 for 4 hours, followed by cell lysis, incubation with Rho-Ub-PA DUB probe, SDS-PAGE, and gel fluorescence scan. The bands corresponding to OTUB2 are highlighted with red rectangles in the fluorescence scans (B) real-time Quantitative PCR (RT-qPCR) analysis for *Otub2* transcripts in BM MDA-MB-231 cells treated with OTUB2 siRNA for 72 hours (Right). Bars represent means ± S.E.M with three samples in each group. Data are representative of four independently performed experiments.

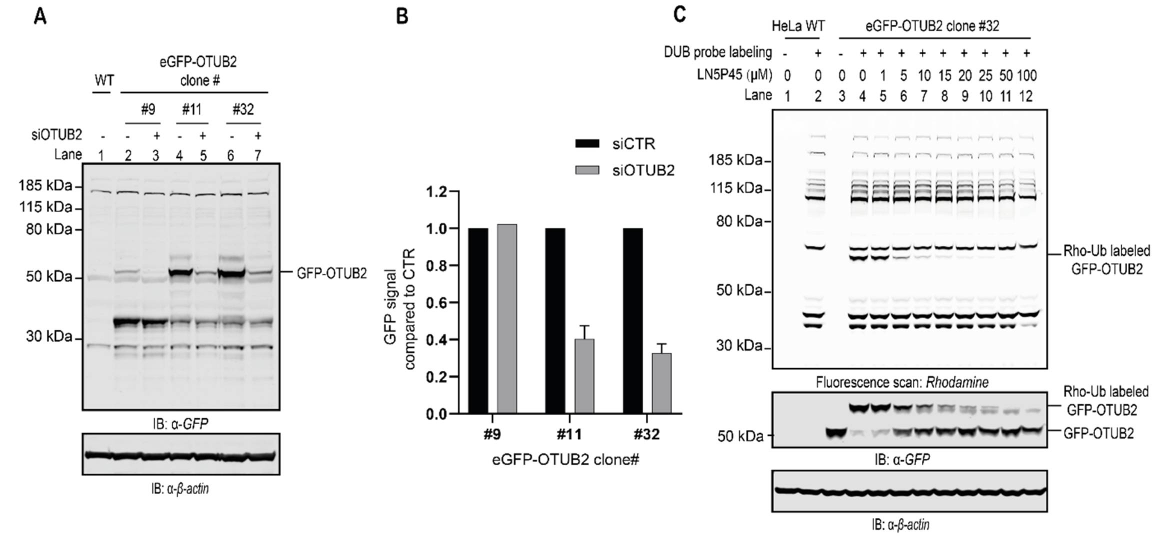

Figure S6. LN5P45 engages endogenous OTUB2 in HeLa cells. (A) Anti-GFP immunoblot validation of HeLa cell clones expressing endogenously tagged GFP-OTUB2 (eGFP-OTUB2 clone #9, #11 and #32). A band corresponding to the molecular weight of GFP-OTUB2 was observed for each clone and the intensity of these bands was reduced by OTUB2 siRNA (siOTUB2). (B) GFP staining of the control siRNA (siCTR) or OTUB2-specific siRNA treated endoGFP-OTUB2 HeLa clones measured by flow cytometry. Bars represent means ± S.E.M with three samples in each group. (C) Clone #32 cells were treated with the indicated concentrations of LN5P45 for 4 hours, followed by cell lysis, incubation with Rho-Ub-PA DUB probe, SDS-PAGE, gel fluorescence scanning, and immunoblotting. Top: fluorescence scan of Rho-Ub-PA probe labelled DUBs. Middle: anti-GFP immunoblot data corresponding to probe labelled and unlabelled GFP-OTUB2. Bottom: anti-β-actin immunoblot data validating equal loading of each sample.

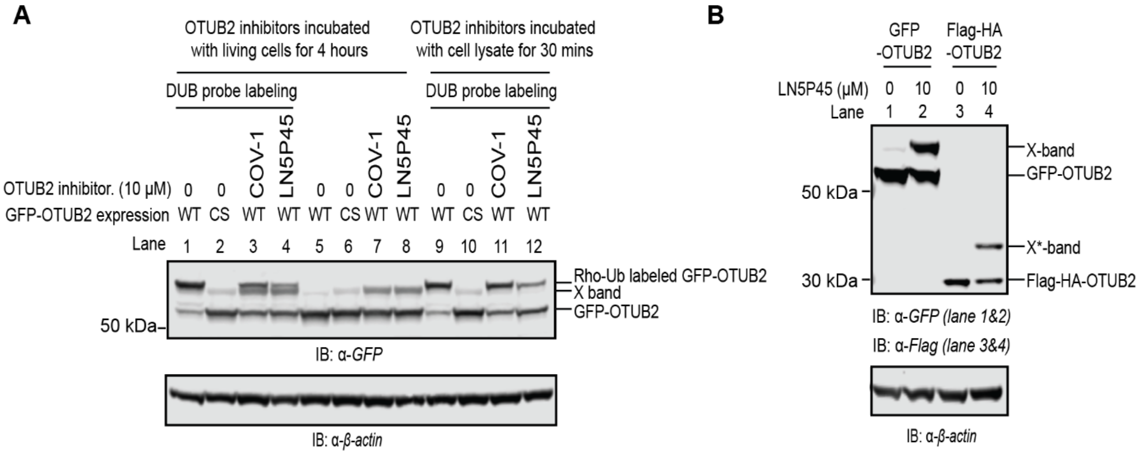

Figure S7. GFP- and Flag-HA-OTUB2 "X-band" mobility shifts upon treatment with LN5P45 inhibitor. (A) HEK293T cells overexpressing GFP-OTUB2 WT or C51S mutant were untreated or treated with 10 μM OTUB2 inhibitors for 4 hours, and after lysis labelled with Rho-Ub-PA probe (lane 1-4), or unlabelled (lane 5-8). Cell lysates from HEK293T cells overexpressing GFP-OTUB2 WT or C51S mutant were incubated with 10 μM OTUB2 inhibitors for 30 min. as indicated, and next labelled with Rho-Ub-PA probe (lane 9-12). Top: anti-GFP immunoblot data corresponding to GFP-OTUB2. Middle: fluorescence scan of Rho-Ub-PA probe labelled DUBs. Bottom: anti-β-actin immunoblot data corresponding to equal loading of each sample. (B) HEK293T cells overexpressing GFP-OTUB2 WT or Flag-HA-OTUB2 WT were treated with 10 μM OTUB2 inhibitors for 4 hours before harvesting, then lysed and subjected to immunoblotting. α-GFP (Rabbit) and α-Flag (Mouse) mixed together for primary antibody incubation. Anti-rabbit 800 and anti-mouse 680 mixed together for secondary antibody incubation.

Table S1. Overview of the purified recombinant human DUBs used in this study.

DUB	Domain	Tag	Uniprot accession number	Source
OTUB2	FL (1-234)	6His tag, cleaved	Q96DC9	*In house*, [Nanao, 2004][1]
OTUD1	CD (270-481)	6His tag	Q5VV17	Ubiquigent (64-0054-050)
OTUB1	FL (1-271)	GST, cleaved	Q96FW1	DUBscan Kit Ubiquigent (67-0006-001)
OTU1 = OTUD2	FL (1-348)	GST, cleaved	Q5VVQ6	
OTUD3	FL (1-398)	GST, cleaved	Q5T2D3	
OTUD6B	FL (1-323)	GST, cleaved	Q8N6M0	
Cezanne	FL (1-843)	GST, cleaved	Q6GQQ9	
UCHL1	FL (1-223)		P09936	*In house*, [Larsen, 1996][2]
USP16	FL (22-823)	N-terminal His-tag	Q9Y5T5	*In house*, [Mons, 2021][3]

Table S2. Data processing and refinement statistics for the OTUB2-**LN5P45** co-crystal structure.

Crystallization conditions	20% (v/v) Isopropanol, 13% (w/v) PEG-4000, 0.1M HEPES pH 8.0
PDB accession code	8CMS
Space group	$P2_1$
Cell dimensions	
a (Å)	47.65
b (Å)	45.25
c (Å)	58.00
α (°)	90
β (°)	96.05
γ (°)	90
Processing statistics	
Resolution (Å)<br>Outer shell (Å)	47.38-1.77<br>1.81-1.77
Beamline	DLS I04-1
Wavelength (Å)	0.920
Observed reflections	76741 (3612)
Unique reflections	24095 (1360)
R_{pim}	0.077 (0.985)
CC(1/2)	0.994 (0.338)
Multiplicity	3.2 (2.7)
Completeness	99.6 (99.6)
Mean (I/σ(I))	7.0 (0.7)
Refinement statistics	
Monomers in ASU	1
No of protein atoms	1944
R_{work}	0.2099
R_{free}	0.2521
RMSD from ideality	
Bond lengths (Å)	0.0097
Bond angles (°)	1.6535
Chiral volume (Å^3)	0.0806
	Values within parentheses are for the outer resolution shell

Table S3. Primers sequences used for site-directed mutagenesis of OTUB2 K12R, K31R, K37R, K44R, K46R and K211R.

Gene	usage	Forward primer (5' → 3')	Reverse (5' → 3')
OTUB2	K12R point mutation	GAAGAATGGATAGAATGTCACATCTTTCTGATATTAGGTTGAAAGATG	CATCTTTCAACCTAATATCAGAAAGATGTGACATTCTATCCATTCTTC
OTUB2	K31R point mutation	TGCTGAGTTCCTCGATTCTCCTCCGGTAAATCCTG	CAGGATTTACCGGAGGAGAATCGAGGAACTCAGCA
OTUB2	K37R point mutation	GATGGCGGTGAACCTTCTGCTGAGTTCCTCGAT	ATCGAGGAACTCAGCAGAAGGTTCACCGCCATC
OTUB2	K44R point mutation	CATCCCCTTTGGTCCTGCGGATGGCGGTG	CACCGCCATCCGCAGGACCAAAGGGGATG
OTUB2	K46R point mutation	CCCATCCCCTCTGGTCTTGCGGATGGCGG	CCGCCATCCGCAAGACCAGAGGGGATGGG
OTUB2	K221R point mutation	GTAGTGGGATGTTCTATAGAGCAGGTAAACGGAAGGGG	CCCCTTCCGTTTACCTGCTCTATAGAACATCCCACTAC

Supporting Materials and Methods

Chemical synthesis of OTUB2 inhibitors

General

General reagents were purchased from Sigma-Aldrich, Biosolve, and Acros, and used as received. Solvents were purchased from Biosolve, Sigma-Aldrich and VWR and used as received. *Trans*-2-phenyl-1-cyclopropanecarboxylic acid (CAS number 939-90-2, article number QB-1747) and (1*R*,2*S*)-Rel-2-phenylcyclopropanecarboxylic acid (CAS number 939-89-9, article number QW-9456) were purchased from Combi-Blocks Inc. (*1S,2R*)-2-phenylcyclopropane-1-carboxylic acid (CAS number 23020-18-0, article number O33621) was purchased from Advanced ChemBlocks Inc.

(*S,S*)-ethyl-2-(3,4-difluorophenyl)cyclopropane-1-carboxylate, (*S,S*)-ethyl-2-(*p*-tolyl)cyclopropane-1-carboxylate, (*S,S*)-ethyl-2-(*m*-tolyl)cyclopropane-1-carboxylate, (*S*,*S*)-ethyl-2-(*o*-tolyl)cyclopropane-1-carboxylate, (*S*,*S*)-ethyl-2-phenylcyclopropane-1-carboxylate, (*R*,*R*)-ethyl-2-phenylcyclopropane-1-carboxylate were synthesized in the Fasan lab using reported procedures.[1-3]

Thin Layer Chromatography (TLC) was performed on Merck aluminium sheets (pre-coated with silica gel 60 F254). Compounds were visualized by UV absorption (254 nm) and by using a solution of $KMnO_4$ (7.5 g/L) and K_2CO_3 (50 g/L) in H_2O or a solution of ninhydrin (15 g/L) in 3% AcOH/EtOH v/v. Compounds (unless stated otherwise) were purified on a Büchi Sepacore automatic flash chromatography system X10/X50. The Büchi Sepacore system was equipped with two Büchi pump modules C-605, a Büchi control unit C-620, a Büchi fraction collector C-660 and a Büchi UV Photometer C-640. The silica columns were purchased at GraceResolv™ and were packed with a grade of Davisil® silica. NMR spectra (^{1}H, ^{13}C) were recorded on a Bruker Ultrashield 300 MHz spectrometer at 298 K. Resonances are indicated with symbols 'd' (doublet), 'ddd' (double double doublet), 'dt' (double triplet), 's' (singlet), 't' (triplet) and 'm' (multiplet). Chemical shifts (δ) are given in ppm relative to $CDCl_3$, DMSO-d6 or CD_3OD as an internal standard and coupling constants (*J*) are given in hertz (Hz).

LC-MS measurements were performed on a LC-MS system equipped with a Waters Acquity H-Class UPLC system with an Extended λ Photodiode Array Detector (210-800 nm), an Acquity BEH C18 Column (130 Å, 1.7 µm, 2.1 mm x 50 mm) and a LCT-Premier ESI-Orthogonal Acceleration Time of Flight Mass Spectrometer. Samples were run using 3 mobile phases: A = Purified deionized Water (Veolia - H_2O), B = Acetonitrile (UPLC grade - CH_3CN) and C = 44% H_2O, 44% CH_3CN, 12% Formic acid (UPLC grade – HCO_2H). Data processing

was performed using Waters MassLynx Mass Spectrometry Software 4.1. UPLC-MS Program: flow rate = 0.5 mL/min, runtime = 3 min, column T = 40 °C, mass detection: 100–1500 Da. Gradient: Line C, provides a constant 4% of the total composition. Initial conditions 94% A, 2% B, 4% C. at 0.2 min. Composition gradually changes over 1.6 min, to 96 %B and 4% C at 1.80 min. This is kept until 2.15 min before changing back to the original composition at 2.20 min and remains so until the 3 min time mark. Electrospray Ionization (ESI) high-resolution mass spectrometry (HR-MS) was carried out on a Waters XEVO-G2 XS Q-TOF mass spectrometer equipped with an electrospray ion source in positive mode (capillary voltage: 3.0 kV, desolvation gas flow: 900 L/h, temperature: 60 °C) with a resolution R = 22,000 using 200 pg μL^{-1} Leu-Enk (*m/z* = 556.2771) as a "lock mass".

General Synthetic Procedures:

A. A solution of 2M TMS-diazomethane in Et_2O (4 eq.) was added dropwise in 15 minutes to a solution of the carboxylic acid in dry MeOH (ca. 0.3M). After stirring at room temperature overnight TLC analysis indicated the reaction to be complete. The mixture was concentrated to dryness *in vacuo* at 40 °C and used as obtained in the next step.

B. The methyl ester (or ethyl ester for the compounds obtained from the Fasan Lab) was dissolved in MeOH (ca. 0.06 – 0.7M). An excess of hydrazine monohydrate (10 – 50 eq.) was added and the mixture was stirred at 60 °C until the starting material had been fully consumed as indicated by TLC and/or LCMS analysis. The mixture was concentrated to dryness *in vacuo* at 40 °C and co-evaporated 3x with toluene. The obtained material was used in the next step without further purification.

C. The crude hydrazide was taken up in DCM (ca. 0.01 – 0.05M). Et_3N (3 eq.) was added, followed by chloroacetylchloride (1.1 eq.) After TLC analysis indicated the reaction to be complete the mixture was diluted ca. 2x with DCM and washed with an equal amount of H_2O. The organic layer was dried ($MgSO_4$), filtered through a paper filter and adsorbed onto Celite. Purification was performed using a silica gel column with an ethyl acetate/heptane gradient as eluent after which the purest fractions were combined and concentrated *in vacuo* at 40 °C.

D. **Enzymatic Cyclopropanation Reactions.** Preparation of the cells expressing engineered myoglobins was done according to literature precedence.[4, 5] Freshly

prepared cell pallets were washed in KPi buffer (50 mM, pH 7, 3x 25 mL) and concentrated to ~OD_{600}=100-120 (cell stock solution). Then, a round-bottom flask was charged with an appropriate amount of KPi buffer which was subsequently degassed with N_2 for 10 minutes. Under positive N_2 pressure, an appropriate amount of cell stock solution was added to the flask to obtain a final cell concentration of OD_{600}=20. The head space of the resulting solution was degassed under N_2 for 10 minutes. A solution with the appropriate olefin in EtOH (1 eq., 10 mM final conc.) was added via syringe through a septum under positive N_2 pressure. The reactions were initiated by the slow addition of an EDA solution in EtOH (3 eq., 30 mM final conc.) using a syringe pump over 3 hours. The reaction was stirred overnight at room temperature under positive N_2 pressure. The crude product was extracted with DCM (3x 50 mL), dried over Na_2SO_4, filtrated, and concentrated under reduced pressure. The crude residue was purified by flash chromatography (hexanes/EtOAc, 95:5) and concentrated *in vacuo*.

Compound Characterization Data

Ethyl (1*R*,2*R*)-2-phenylcyclopropane-1-carboxylate (13a):

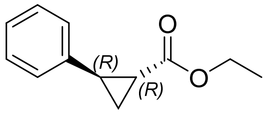

Compound **13a** was synthesized according to general procedure D. Yield: 500 mg, 2.63 mmol, 48% isolated yield. Compound characterization data was consistent with previous reports.[6]

Ethyl (1*S*,2*S*)-2-phenylcyclopropane-1-carboxylate (13b):

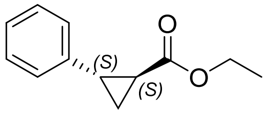

Compound **13b** was synthesized according to general procedure D. Yield: 1.0 g, 5.26 mmol, 95% isolated yield. Compound characterization data was consistent with previous reports.[4]

Ethyl (1*S*,2*S*)-2-(o-tolyl)cyclopropane-1-carboxylate (13c):

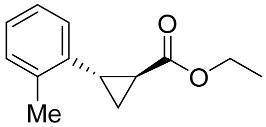

Compound **13c** was synthesized according to general procedure D. Yield: 477 mg, 2.34 mmol, 78% isolated yield. Compound characterization data was consistent with previous reports.[4]

Ethyl (1*S*,2*S*)-2-(m-tolyl)cyclopropane-1-carboxylate (13d):

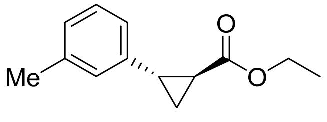

Compound **13d** was synthesized according to general procedure D. Yield: 220 mg, 1.08 mmol, 36% isolated yield. Compound characterization data was consistent with previous reports.[4]

Ethyl (1*S*,2*S*)-2-(p-tolyl)cyclopropane-1-carboxylate (13e):

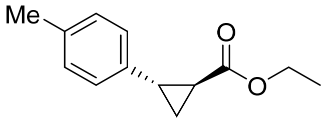

Compound **13e** was synthesized according to general procedure D. Yield: 206 mg, 1.01 mmol, 34% isolated yield. Compound characterization data was consistent with previous reports.[4]

Ethyl (1*S*,2*S*)-2-(2,3-difluorophenyl)cyclopropane-1-carboxylate (13f):

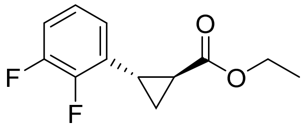

Compound **13f** was synthesized according to general procedure D. Yield: 63 mg, 0.277 mmol, 15% isolated yield. Compound characterization data was consistent with previous reports.[6]

***Trans N'*-(2-chloroacetyl)-2-phenylcyclopropane-1-carbohydrazide (2)**

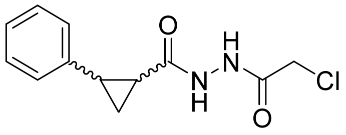

1:1 mix *trans* configuration

The compound was synthesized according to general procedures A, B and C starting from commercially available *trans*-2-phenyl-1-cyclopropanecarboxylic acid and obtained as a white solid. Yield: 3 mg, 0.012 mmol, 11 % over 3 steps. ^{1}H NMR (300 MHz, DMSO-d_6) δ 10.28 (d, J = 14.1 Hz, 2H), 7.37 – 7.10 (m, 5H), 4.14 (s, 2H), 2.31 (ddd, J = 9.0, 6.3, 4.1 Hz, 1H), 1.96 (ddd, J = 8.3, 5.3, 4.1 Hz, 1H), 1.48 – 1.30 (m, 2H). ^{13}C NMR (75 MHz, DMSO-d_6) δ 170.49, 165.22, 141.00, 128.85, 126.62, 126.30, 41.33, 24.83, 24.31, 15.97. HR-MS calculated for $C_{12}H_{13}ClN_2O_2$ [M+H]$^+$ 253.0744, found 253.0740.

Cis N'-(2-chloroacetyl)-2-phenylcyclopropane-1-carbohydrazide (3)

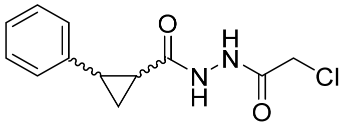

1:1 mix *cis* configuration

The compound was synthesized according to general procedures A, B and C starting from commercially available *cis*-2-phenylcyclopropane-1-carboxylate and obtained as a white solid. Yield: 10 mg, 0.040 mmol, 28 % over 3 steps. ^{1}H NMR (300 MHz, Chloroform-*d*) δ 9.41 (d, *J* = 5.3 Hz, 1H), 8.93 (d, *J* = 5.1 Hz, 1H), 7.25 – 7.05 (m, 5H), 3.79 (s, 2H), 2.48 (q, *J* = 8.5 Hz, 1H), 1.89 (ddd, *J* = 9.0, 7.9, 5.5 Hz, 1H), 1.67 (dt, *J* = 7.6, 5.3 Hz, 1H), 1.30 (ddd, *J* = 8.8, 7.9, 5.0 Hz, 1H). ^{13}C NMR (75 MHz, Chloroform-*d*) δ 167.77, 163.00, 136.16, 129.26, 127.98, 126.74, 40.66, 25.91, 20.89, 10.95. HR-MS calculated for $C_{12}H_{13}ClN_2O_2$ $[M+H]^+$ 253.0744, found 253.0739.

(1*S*,2*R*)-*N'*-(2-chloroacetyl)-2-phenylcyclopropane-1-carbohydrazide (4)

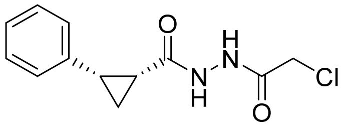

The compound was synthesized according to general procedures A, B and C starting from commercially available (1*S*,2*R*)-2-phenylcyclopropane-1-carboxylate and obtained as a white solid. Yield: 8 mg, 0.031 mmol, 24 % over 3 steps. ^{1}H NMR (300 MHz, Chloroform-*d*) δ 9.26 (d, *J* = 5.1 Hz, 1H), 8.80 (d, *J* = 4.9 Hz, 1H), 7.22 – 7.07 (m, 5H), 3.80 (d, *J* = 1.6 Hz, 2H), 2.49 (q, *J* = 8.5 Hz, 1H), 1.90 (ddd, *J* = 9.0, 7.9, 5.5 Hz, 1H), 1.69 (dt, *J* = 7.6, 5.3 Hz, 1H), 1.31 (ddd, *J* = 8.8, 7.9, 5.0 Hz, 1H). ^{13}C NMR (75 MHz, Chloroform-*d*) δ 167.85, 163.13, 136.10, 129.25, 128.00, 126.75, 40.67, 25.97, 21.04, 10.98. HR-MS calculated for $C_{12}H_{13}ClN_2O_2$ $[M+H]^+$ 253.0744, found 253.0741.

(1*R*,2*R*)-*N'*-(2-chloroacetyl)-2-phenylcyclopropane-1-carbohydrazide (5)

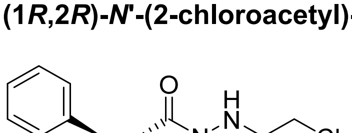

The compound was synthesized according to general procedures B and C starting from (*R*,*R*)-ethyl-2-phenylcyclopropane-1-carboxylate **13a** provided by Fasan Lab and obtained as

a white solid. Yield: 12.3 mg, 0.049 mmol, 9 % over 2 steps. ^{1}H NMR (300 MHz, DMSO-d_6) δ 10.30 (d, *J* = 2.1 Hz, 1H), 10.25 (d, *J* = 2.1 Hz, 1H), 7.33 – 7.13 (m, 5H), 4.13 (s, 2H), 2.29 (ddd, *J* = 9.0, 6.3, 4.1 Hz, 1H), 1.94 (ddd, *J* = 8.3, 5.4, 4.1 Hz, 1H), 1.46 – 1.26 (m, 2H). ^{13}C NMR (75 MHz, DMSO-d_6) δ 170.47, 165.22, 141.01, 128.85, 126.61, 126.30, 41.32, 24.81, 24.32, 15.96. HR-MS calculated for $C_{12}H_{13}ClN_2O_2$ $[M+H]^+$ 253.0744, found 253.0740.

(1*S*,2*S*)-*N'*-(2-chloroacetyl)-2-phenylcyclopropane-1-carbohydrazide (6)

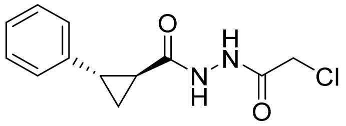

The compound was synthesized according to general procedures B and C starting (*S*,*S*)-ethyl-2-phenylcyclopropane-1-carboxylate **13b** and obtained as a white solid. Yield: 8.3 mg, 0.033 mmol, 6 % over 2 steps. ^{1}H NMR (300 MHz, DMSO-d_6) δ 10.30 (s, 1H), 10.25 (s, 1H), 7.36 – 7.25 (m, 2H), 7.24 – 7.12 (m, 3H), 4.13 (s, 2H), 2.29 (ddd, *J* = 8.9, 6.4, 4.2 Hz, 1H), 1.94 (ddd, *J* = 8.3, 5.4, 4.1 Hz, 1H), 1.39 (ddd, *J* = 9.2, 5.4, 4.1 Hz, 1H), 1.35 – 1.30 (m, 1H). ^{13}C NMR (75 MHz, DMSO-d_6) δ 170.47, 165.22, 141.01, 128.85, 126.61, 126.29, 41.32, 24.81, 24.32, 15.96. HR-MS calculated for $C_{12}H_{13}ClN_2O_2$ $[M+H]^+$ 253.0744, found 253.0738.

(1*S*,2*S*)-*N'*-(2-chloroacetyl)-2-(*o*-tolyl)cyclopropane-1-carbohydrazide (7)

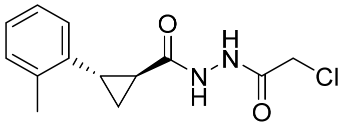

The compound was synthesized according to general procedures B and C starting from (*S*,*S*)-ethyl-2-(*o*-tolyl)cyclopropane-1-carboxylate **13c** and obtained as a white solid. Yield: 8.2 mg, 0.031 mmol, 13 % over 2 steps. ^{1}H NMR (300 MHz, DMSO-d_6) δ 10.33 (d, *J* = 2.1 Hz, 1H), 10.25 (d, *J* = 2.2 Hz, 1H), 7.22 – 7.07 (m, 3H), 7.07 – 6.98 (m, 1H), 4.13 (s, 2H), 2.34 – 2.24 (m, 4H), 1.79 – 1.70 (m, 1H), 1.37 – 1.31 (m, 1H). ^{13}C NMR (75 MHz, DMSO-d_6) δ 171.01, 165.23, 138.60, 137.86, 130.02, 126.85, 126.36, 125.90, 41.34, 23.18, 22.32, 19.69, 13.68. HR-MS calculated for $C_{13}H_{15}ClN_2O_2$ $[M+H]^+$ 267.0900, found 267.0892.

(1*S*,2*S*)-*N'*-(2-chloroacetyl)-2-(*m*-tolyl)cyclopropane-1-carbohydrazide (8, LN5P45)

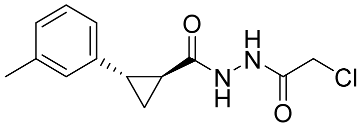

The compound was synthesized according to general procedures B and C starting from (*S*,*S*)-ethyl-2-(*m*-tolyl)cyclopropane-1-carboxylate **13d** and obtained as a white solid. Yield: 6.1 mg, 0.023 mmol, 10 % over 2 steps. ^{1}H NMR (300 MHz, DMSO-d_6) δ 10.24 (s, 2H), 7.17 (t, *J* = 48.7, 7.5 Hz, 1H), 7.06 – 6.88 (m, 3H), 4.13 (s, 2H), 2.30 – 2.20 (m, 4H), 1.96 – 1.88 (m, 1H), 1.42 – 1.33 (m, 1H), 1.33 – 1.29 (m, 1H). ^{13}C NMR (75 MHz, DMSO-d_6) δ 170.52, 165.21, 140.88, 137.95, 128.74, 127.28, 126.96, 123.41, 41.32, 24.80, 24.17, 21.44, 15.81. HR-MS calculated for $C_{12}H_{13}ClN_2O_2$ [M+H]$^+$ 267.0900, found 267.0897.

(1*S*,2*S*)-*N'*-(2-chloroacetyl)-2-(*p*-tolyl)cyclopropane-1-carbohydrazide (9)

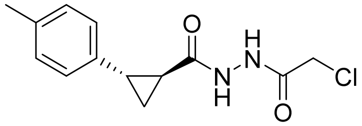

The compound was synthesized according to general procedures B and C starting from (*S*,*S*)-ethyl-2-(*p*-tolyl)cyclopropane-1-carboxylate **13e** and obtained as a white solid. Yield: 2.2 mg, 0.0082 mmol, 3 % over 2 steps. ^{1}H NMR (300 MHz, DMSO-d_6) δ 10.25 (s, 2H), 7.10 (d, *J* = 7.7 Hz, 2H), 7.03 (d, *J* = 8.2 Hz, 2H), 4.13 (s, 2H), 2.30 – 2.21 (m, 4H), 1.89 (ddd, *J* = 8.3, 5.3, 4.1 Hz, 1H), 1.36 (ddd, *J* = 9.2, 5.3, 4.0 Hz, 1H), 1.31 – 1.26 (m, 1H). ^{13}C NMR (75 MHz, DMSO-d_6) δ 170.56, 165.21, 137.88, 135.63, 129.40, 126.22, 41.32, 24.56, 24.08, 21.05, 15.83. HR-MS calculated for $C_{12}H_{13}ClN_2O_2$ [M+H]$^+$ 267.0900, found 267.0895.

(1*S*,2*S*)-*N'*-(2-chloroacetyl)-2-(3,4-difluorophenyl)cyclopropane-1-carbohydrazide (10)

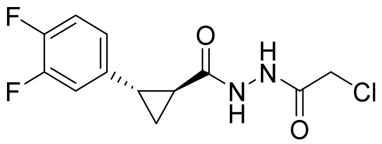

The compound was synthesized according to general procedures B and C starting from (*S*,*S*)-ethyl-2-(3,4-difluorophenyl)cyclopropane-1-carboxylate **13f** and obtained as a white solid. Yield: 6.5 mg, 0.023 mmol, 20 % over 2 steps. ^{1}H NMR (300 MHz, DMSO-d_6) δ 10.30

(d, J = 2.1 Hz, 1H), 10.25 (d, J = 2.2 Hz, 1H), 7.42 – 7.30 (m, 1H), 7.31 – 7.20 (m, 1H), 7.07 – 6.99 (m, 1H), 4.12 (s, 2H), 2.33 (ddd, J = 8.8, 6.5, 4.1 Hz, 1H), 1.98 – 1.89 (m, 1H), 1.43 – 1.33 (m, 2H). ^{13}C NMR (75 MHz, DMSO-d_6) δ 170.10, 165.19, 151.49, 150.02, 148.25, 146.79, 139.12, 123.21, 117.77, 115.29, 41.30, 24.56, 23.94, 16.10. $C_{12}H_{11}ClF_2N_2O_2$ $[M+H]^+$ 289.0555, found 289.0547.

Synthesis and purification of the DBIA probe

The chemical synthesis and purification of the DBIA probe was performed using the procedure published before.[7]

Supplementary References

(1) Nanao, M. H.; Tcherniuk, S. O.; Chroboczek, J.; Dideberg, O.; Dessen, A.; Balakirev, M. Y., Crystal structure of human otubain 2. *EMBO Rep.* **2004,** *5*, 783-788.

(2) Larsen, C. N.; Price, J. S.; Wilkinson, K. D., Substrate binding and catalysis by ubiquitin C-terminal hydrolases: identification of two active site residues. *Biochemistry* **1996,** *35*, 6735-6744.

(3) Mons, E.; Kim, R. Q.; van Doodewaerd, B. R.; van Veelen, P. A.; Mulder, M. P. C.; Ovaa, H., Exploring the Versatility of the Covalent Thiol-Alkyne Reaction with Substituted Propargyl Warheads: A Deciding Role for the Cysteine Protease. *J. Am. Chem. Soc.* **2021,** *143*, 6423-6433.

(4) Bordeaux, M.; Tyagi, V.; Fasan, R., Highly diastereoselective and enantioselective olefin cyclopropanation using engineered myoglobin-based catalysts. *Angew. Chem. Int. Ed.* **2015,** *54*, 1744-1748.

(5) Sreenilayam, G.; Moore, E. J.; Steck, V.; Fasan, R., Stereoselective olefin cyclopropanation under aerobic conditions with an artificial enzyme incorporating an iron-chlorin e6 cofactor. *ACS Catal.* **2017,** *7*, 7629-7633.

(6) Bajaj, P.; Sreenilayam, G.; Tyagi, V.; Fasan, R., Gram-Scale Synthesis of Chiral Cyclopropane-Containing Drugs and Drug Precursors with Engineered Myoglobin Catalysts Featuring Complementary Stereoselectivity. *Angew. Chem. Int. Ed.* **2016,** *55*, 16110-16114.

(7) Kuljanin, M.; Mitchell, D. C.; Schweppe, D. K.; Gikandi, A. S.; Nusinow, D. P.; Bulloch, N. J.; Vinogradova, E. V.; Wilson, D. L.; Kool, E. T.; Mancias, J. D.; Cravatt, B. F.; Gygi, S. P., Reimagining high-throughput profiling of reactive cysteines for cell-based screening of large electrophile libraries. *Nat. Biotechnol.* **2021,** *39*, 630-641.

^{1}H NMR and ^{13}C NMR of compound 2 in DMSO-d6

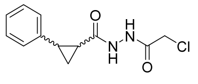

1:1 mix *trans* configuration

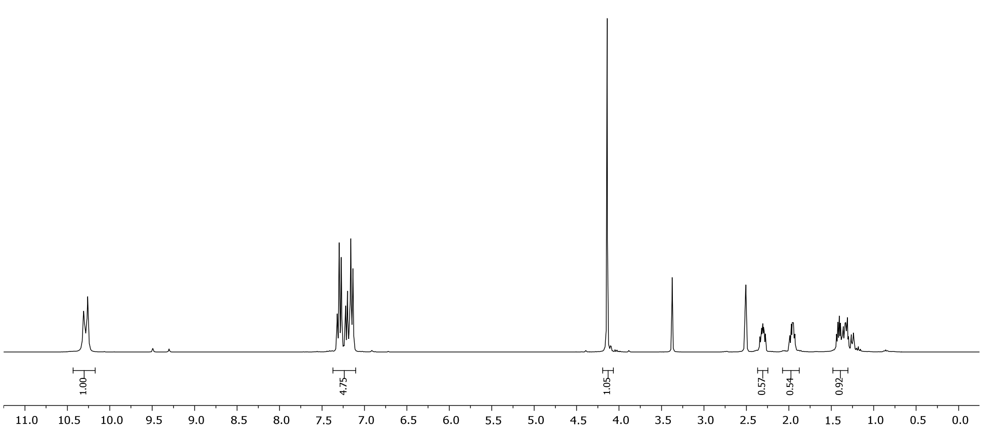

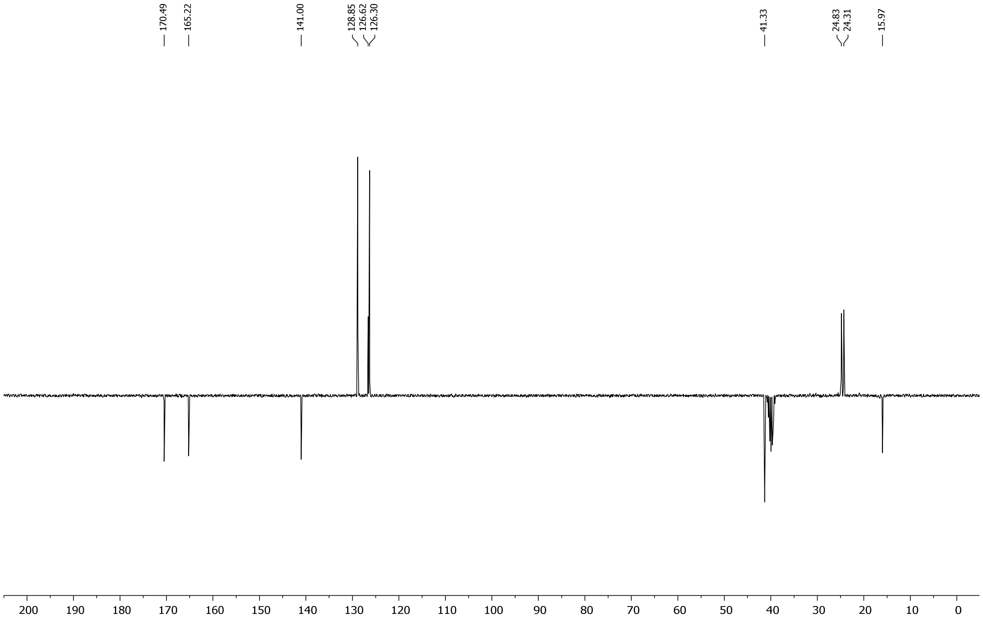

^{1}H NMR and ^{13}C NMR of compound 3 in CDCl

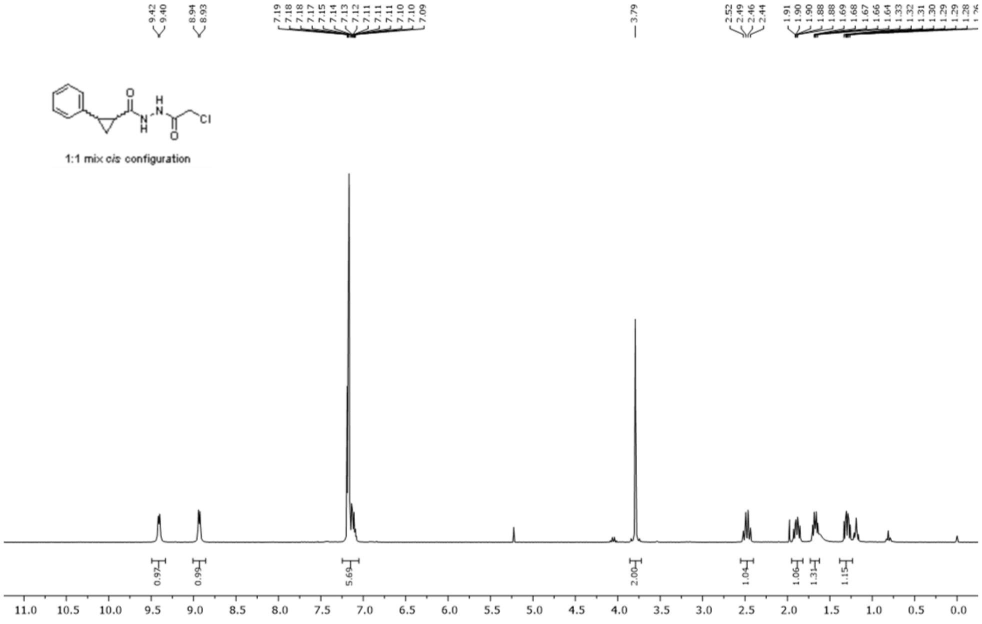

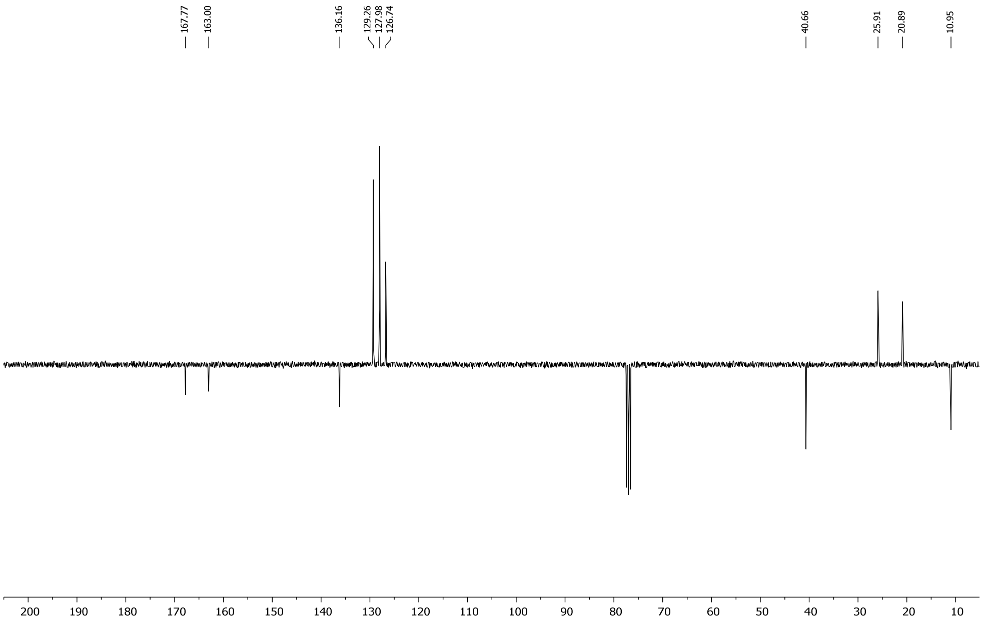

^{1}H NMR and ^{13}C NMR of compound 4 in $CDCl_3$

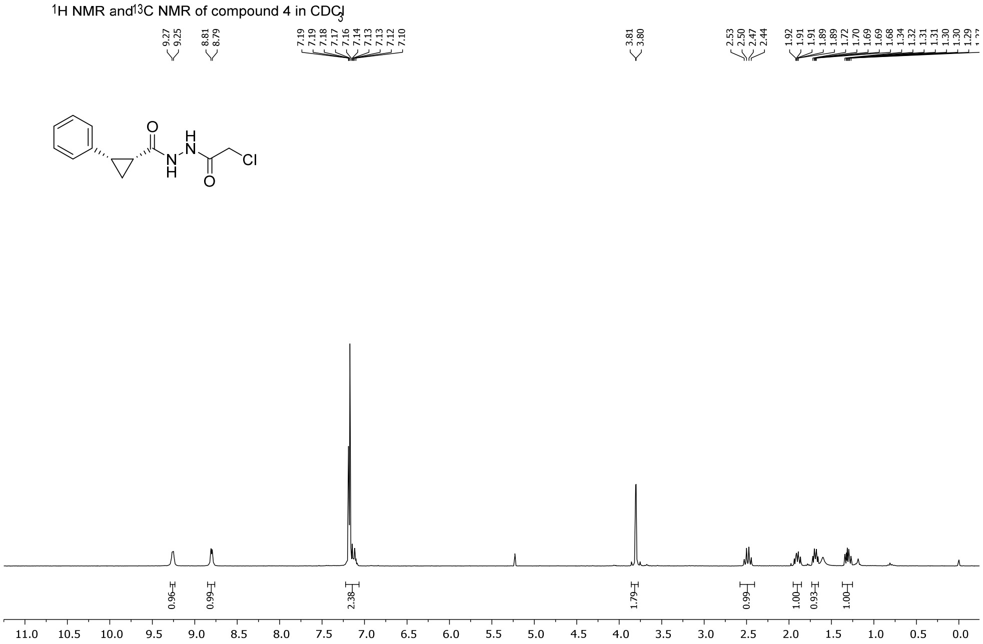

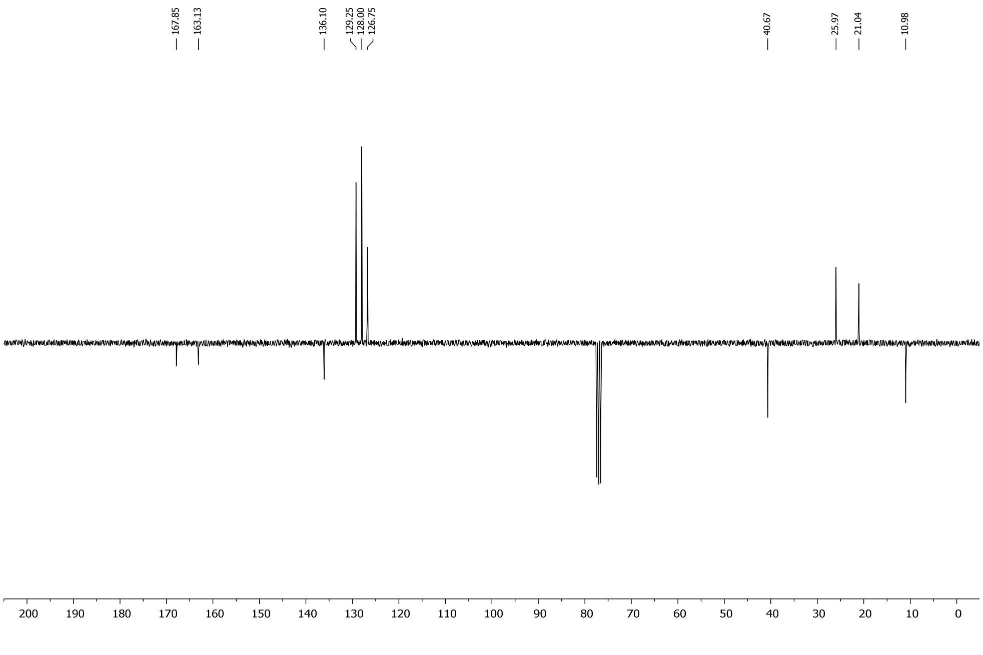

^{1}H NMR and ^{13}C NMR of compound 5 in DMSO-d6

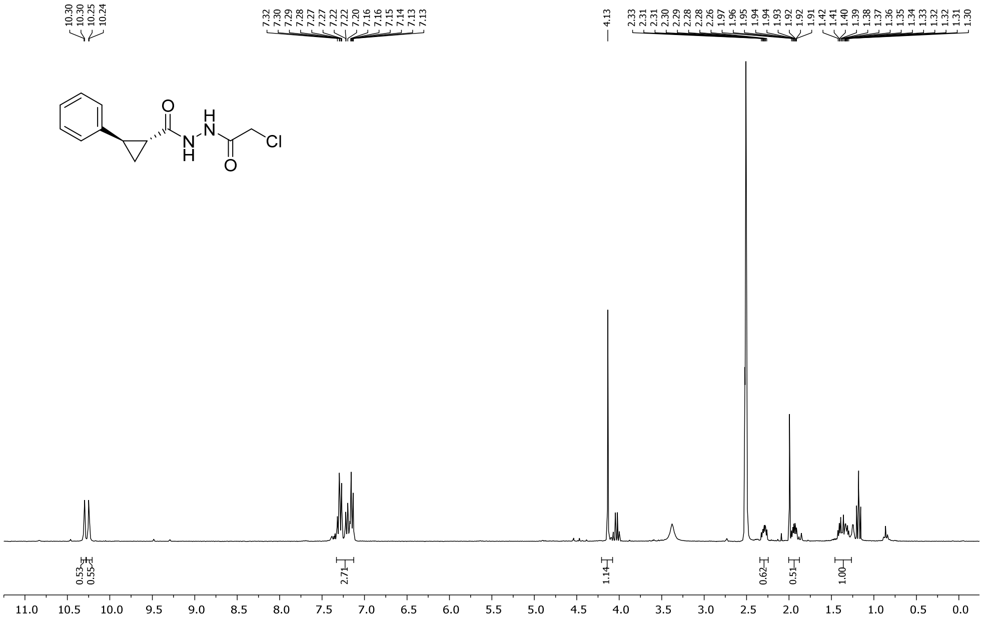

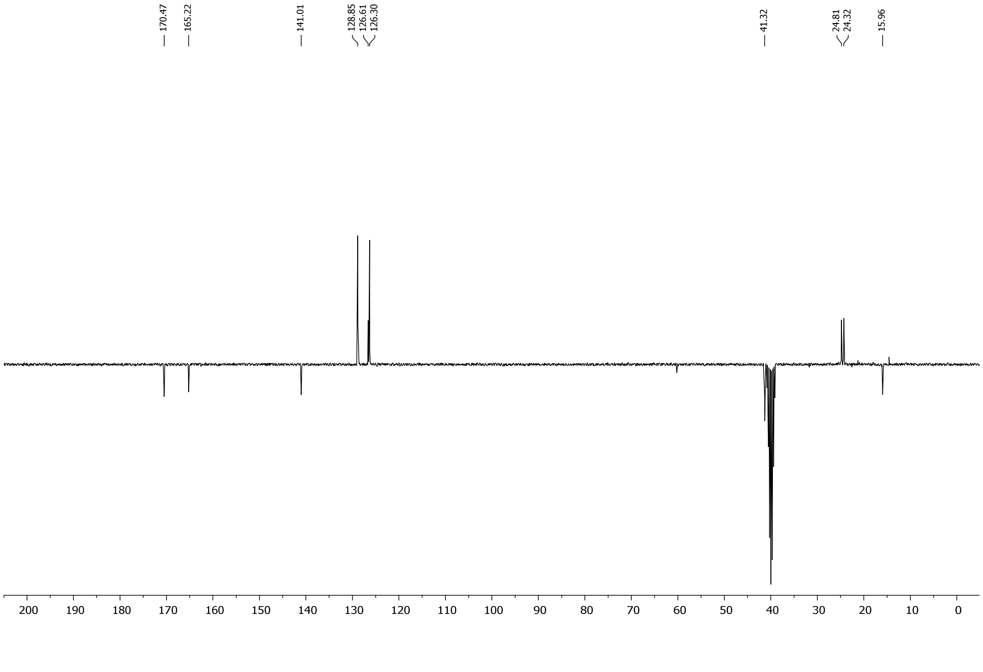

^{1}H NMR and^{13}C NMR of compound 6 in DMSO-d6

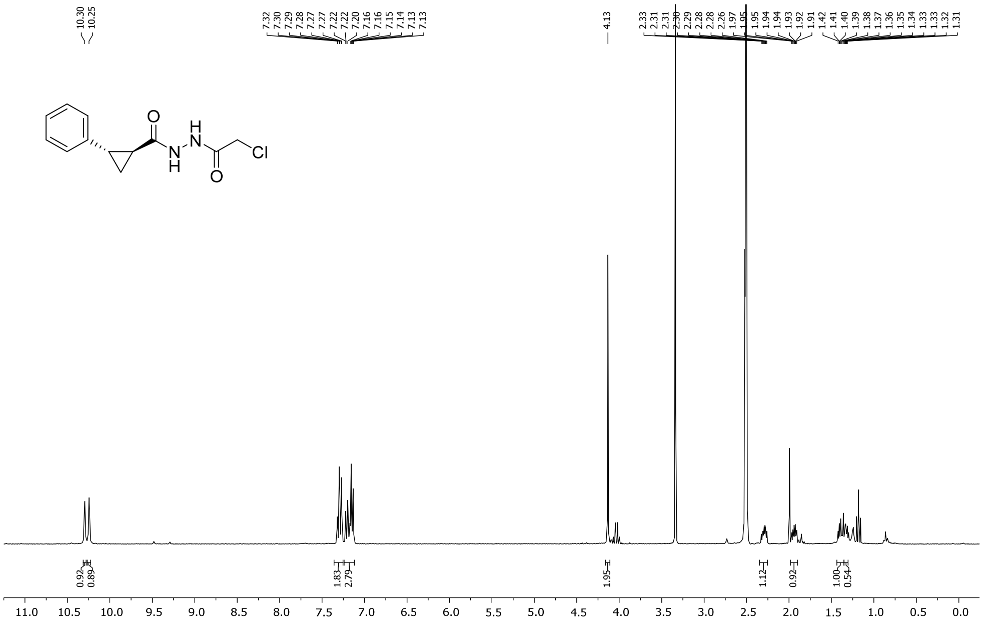

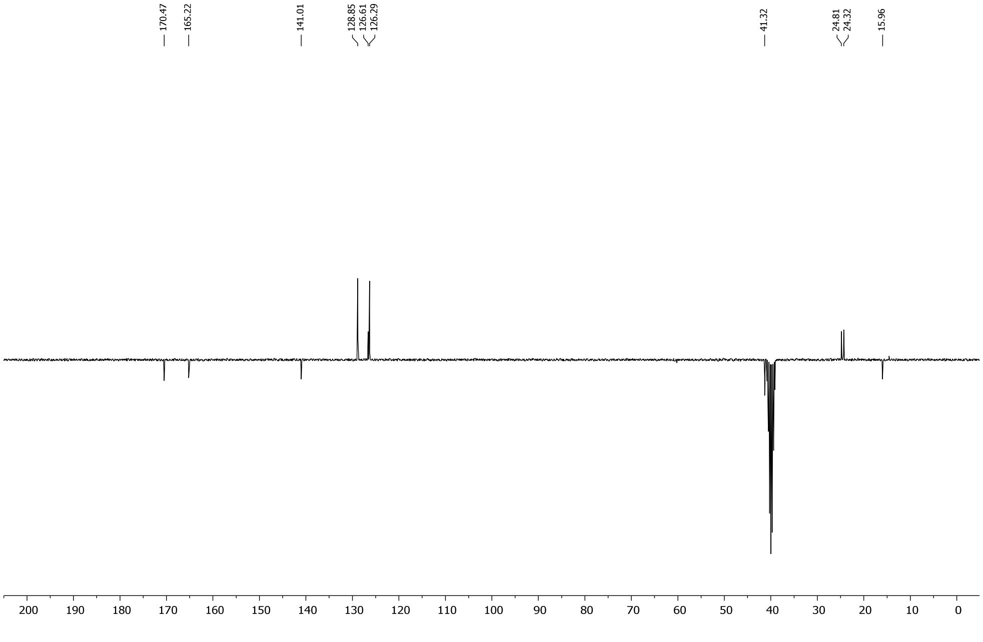

^{1}H NMR and^{13}C NMR of compound 7 in DMSO-d6

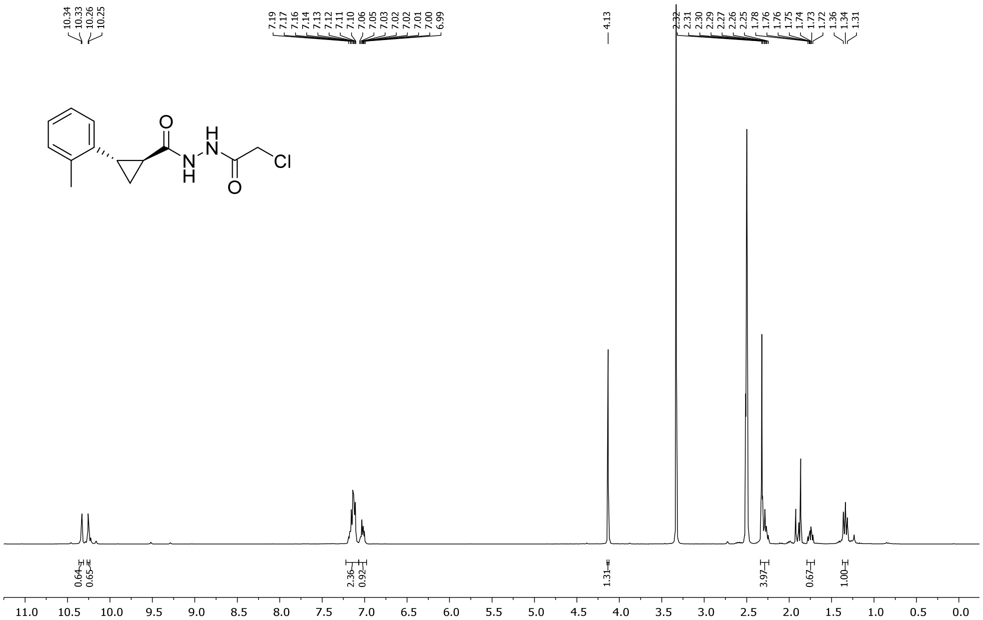

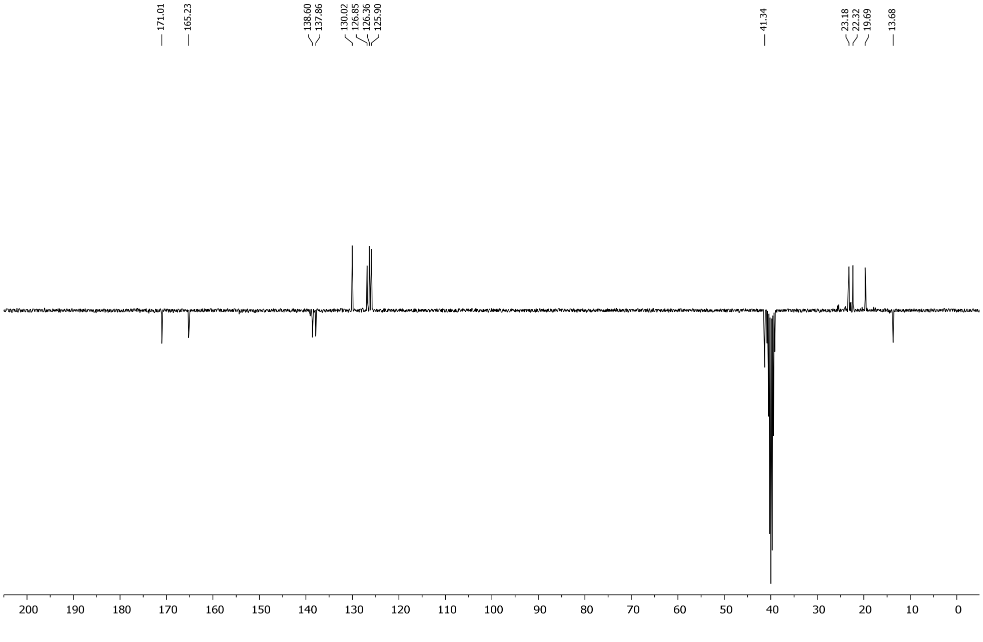

^{1}H NMR and^{13}C NMR of compound 8 in DMSO-d6

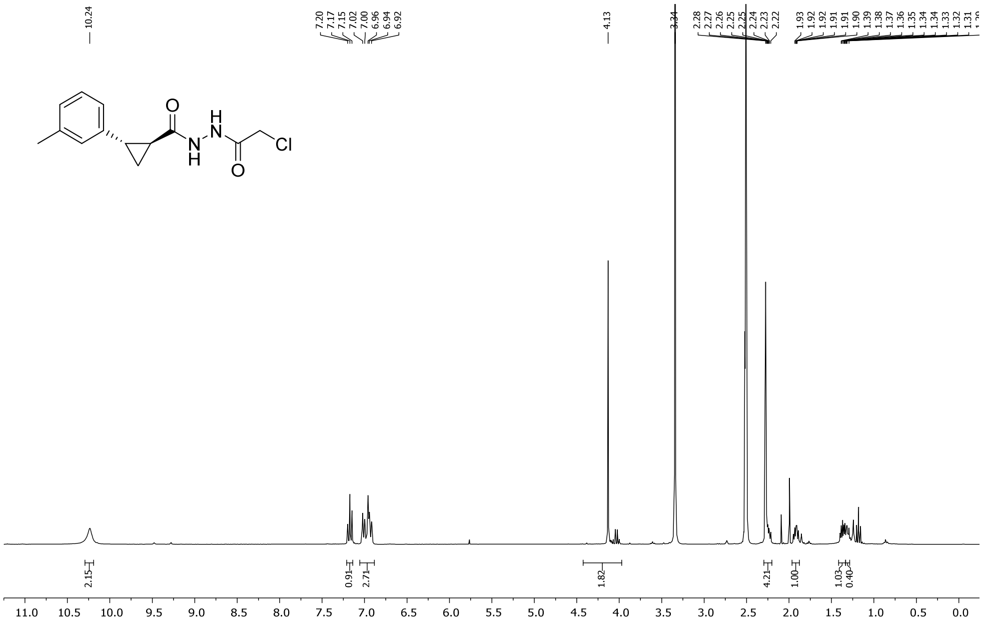

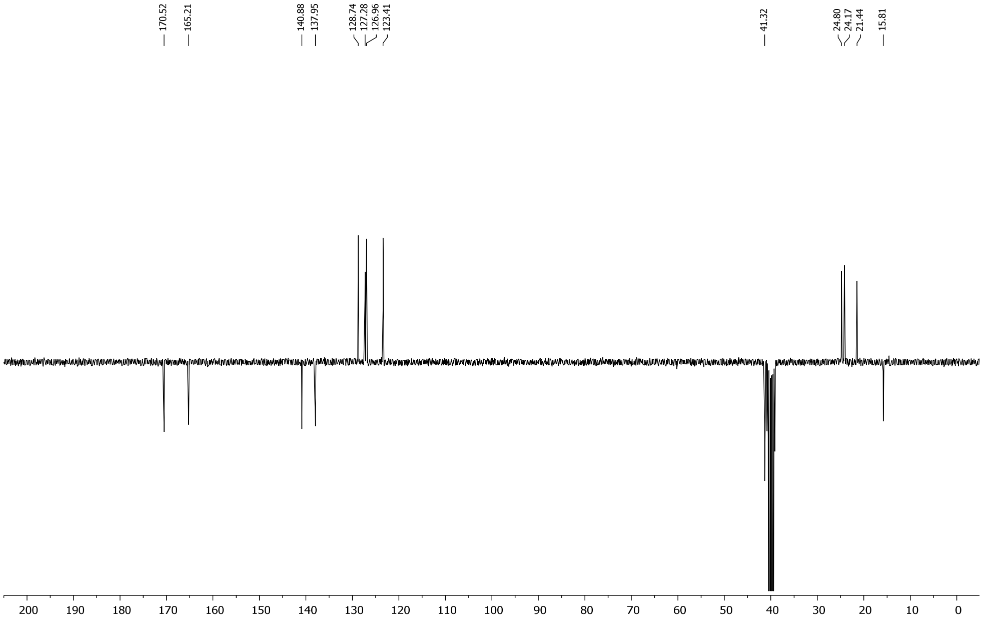

^{1}H NMR and ^{13}C NMR of compound 9 in DMSO-d6

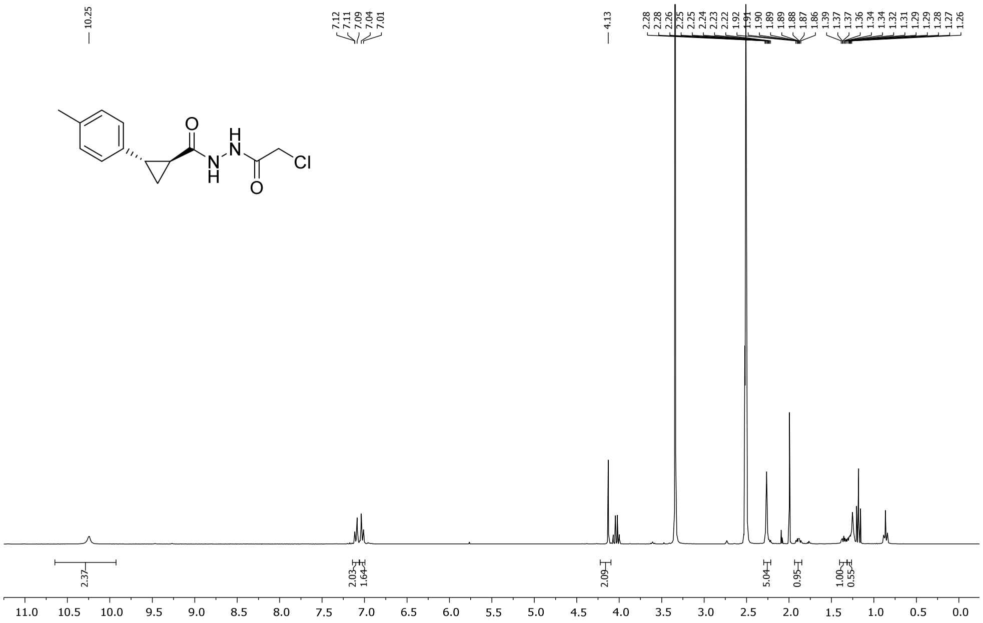

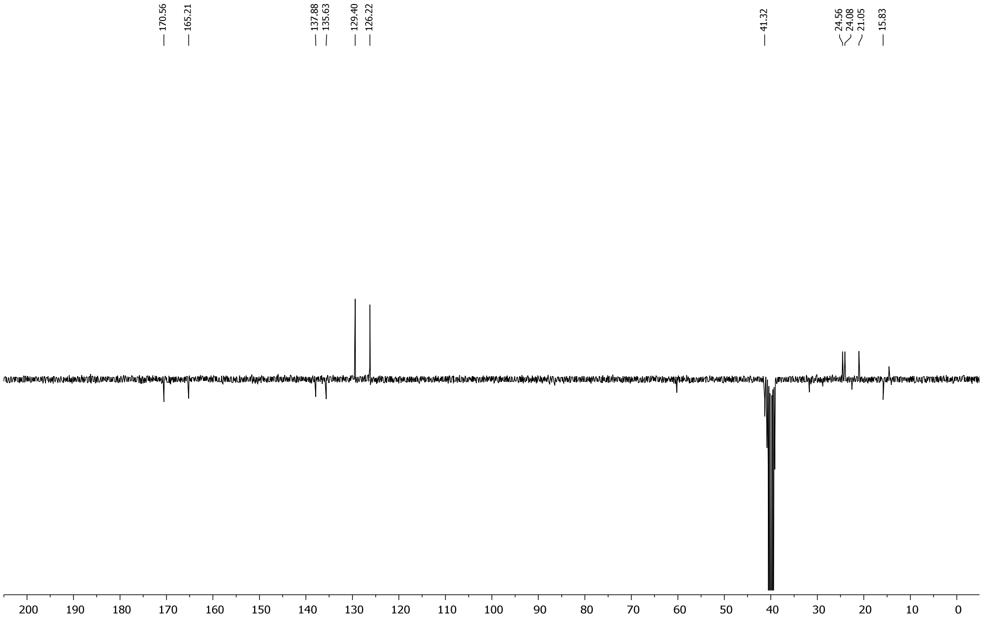

^{1}H NMR and ^{13}C NMR of compound 10 in DMSO-d6

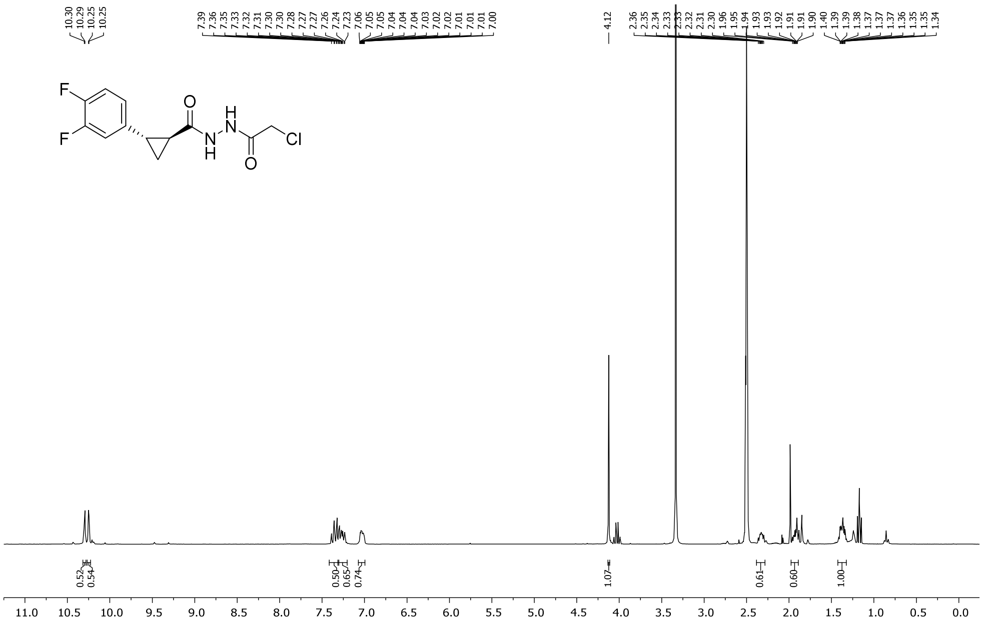

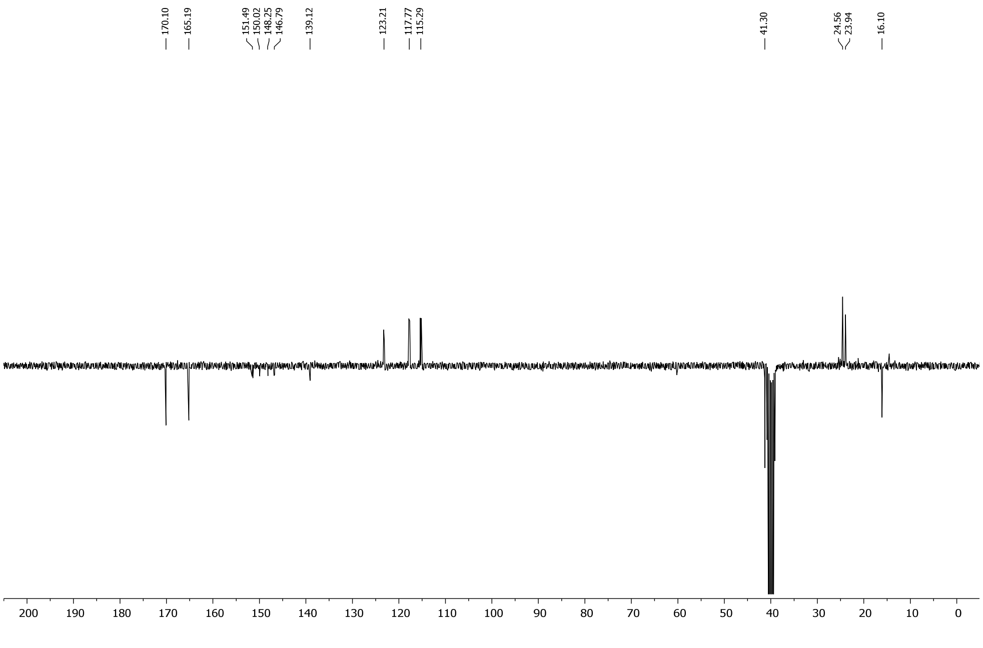

Compound 2

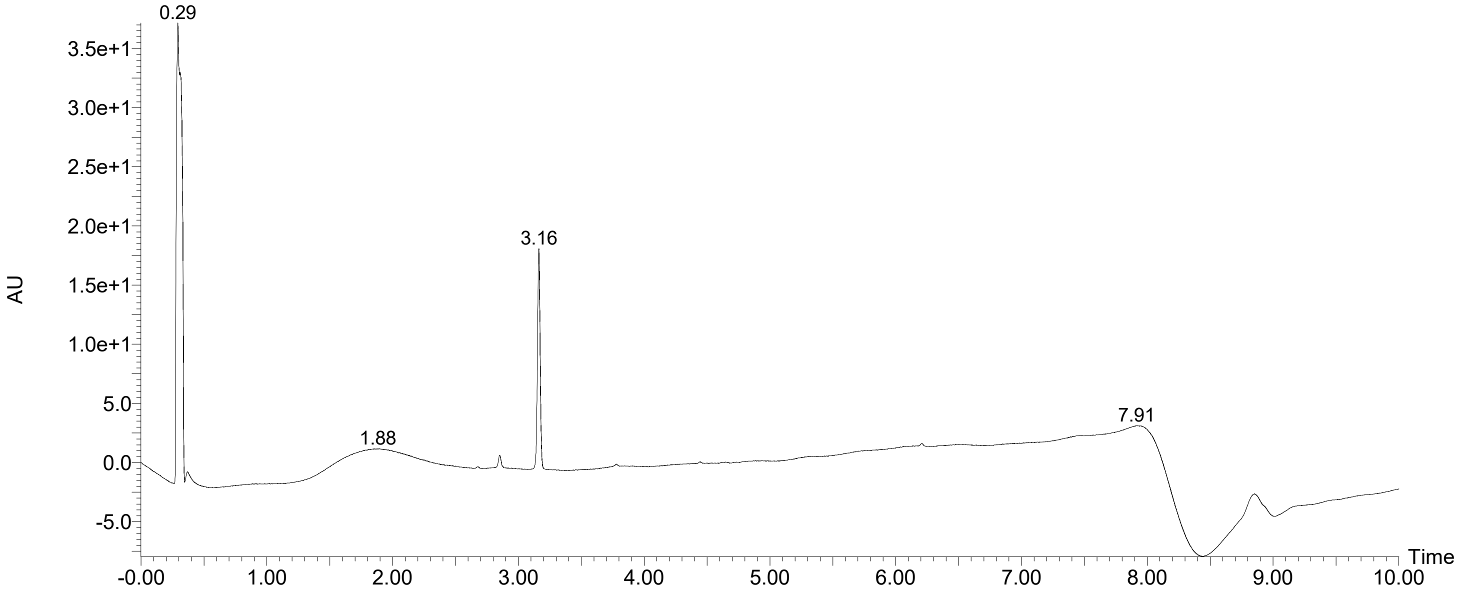

0.29
3.5e+1
3.0e+1
2.5e+1
2.0e+1
1.5e+1
1.0e+1
5.0
0.0
-5.0
AU
3.16
1.88
7.91
Time
-0.00
1.00
2.00
3.00
4.00
5.00
6.00
7.00
8.00
9.00
10.00

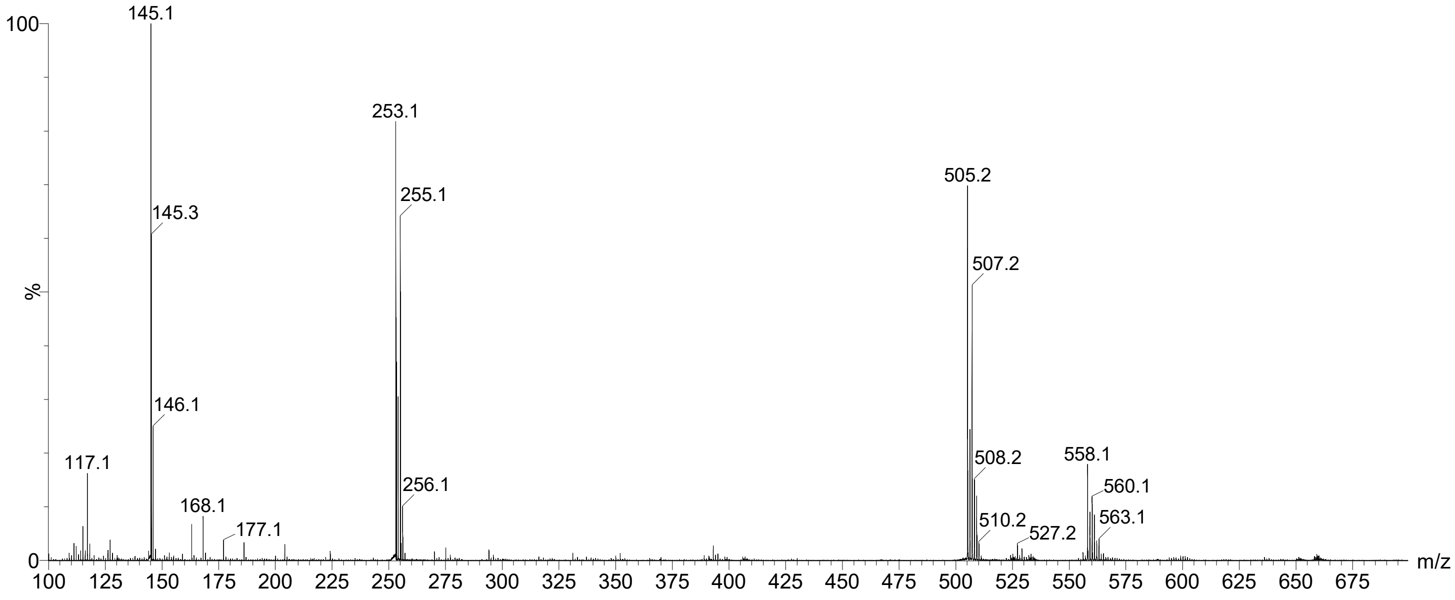

100
%
0
145.1
145.3
146.1
117.1
168.1
177.1
253.1
255.1
256.1
505.2
507.2
508.2
510.2
527.2
558.1
560.1
563.1
m/z
100 125 150 175 200 225 250 275 300 325 350 375 400 425 450 475 500 525 550 575 600 625 650 675

Compound 3

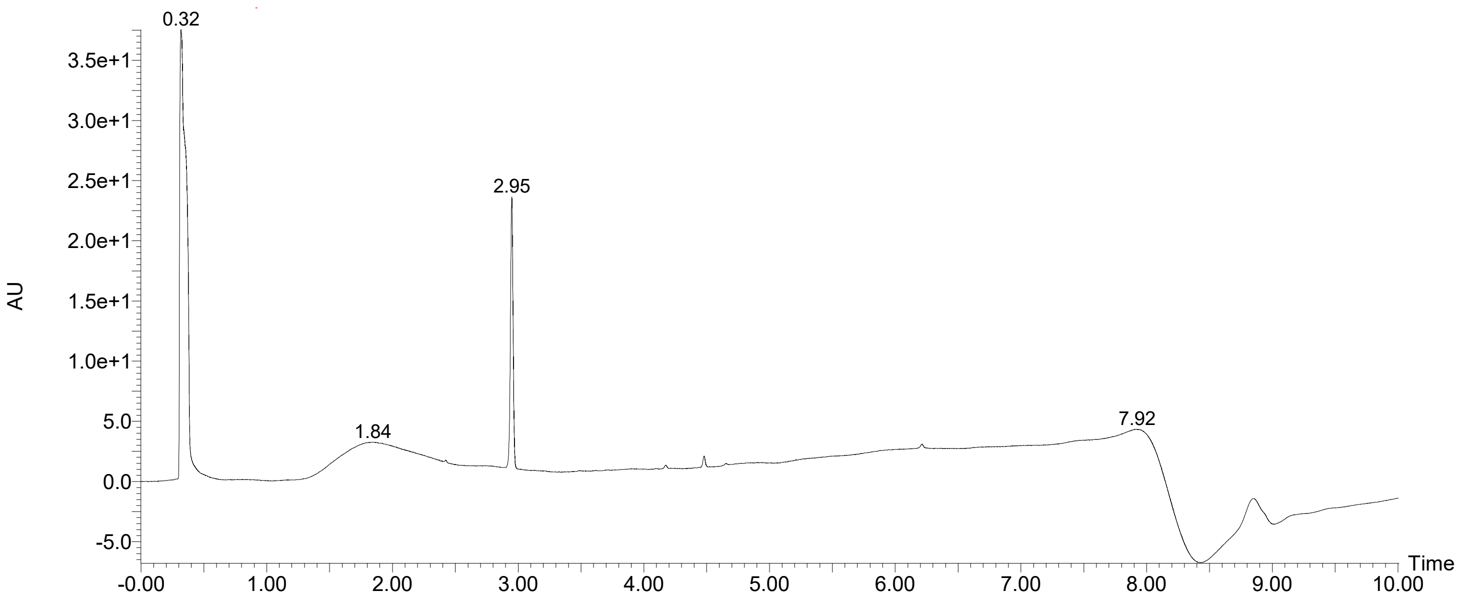
0.32
2.95
1.84
7.92
AU
3.5e+1
3.0e+1
2.5e+1
2.0e+1
1.5e+1
1.0e+1
5.0
0.0
-5.0
Time
-0.00 1.00 2.00 3.00 4.00 5.00 6.00 7.00 8.00 9.00 10.00

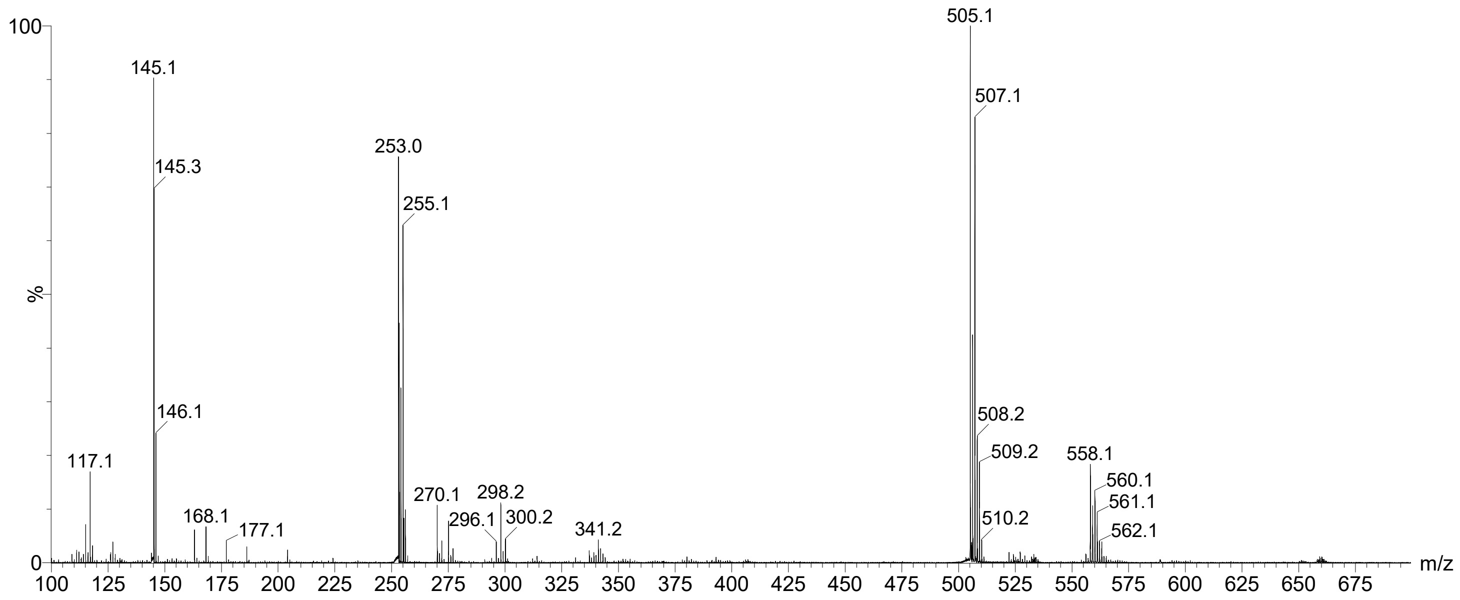
505.1
507.1
145.1
145.3
146.1
117.1
168.1
177.1
253.0
255.1
270.1
296.1
298.2
300.2
341.2
508.2
509.2
510.2
558.1
560.1
561.1
562.1
100
%
0
m/z
100 125 150 175 200 225 250 275 300 325 350 375 400 425 450 475 500 525 550 575 600 625 650 675

Compound 4

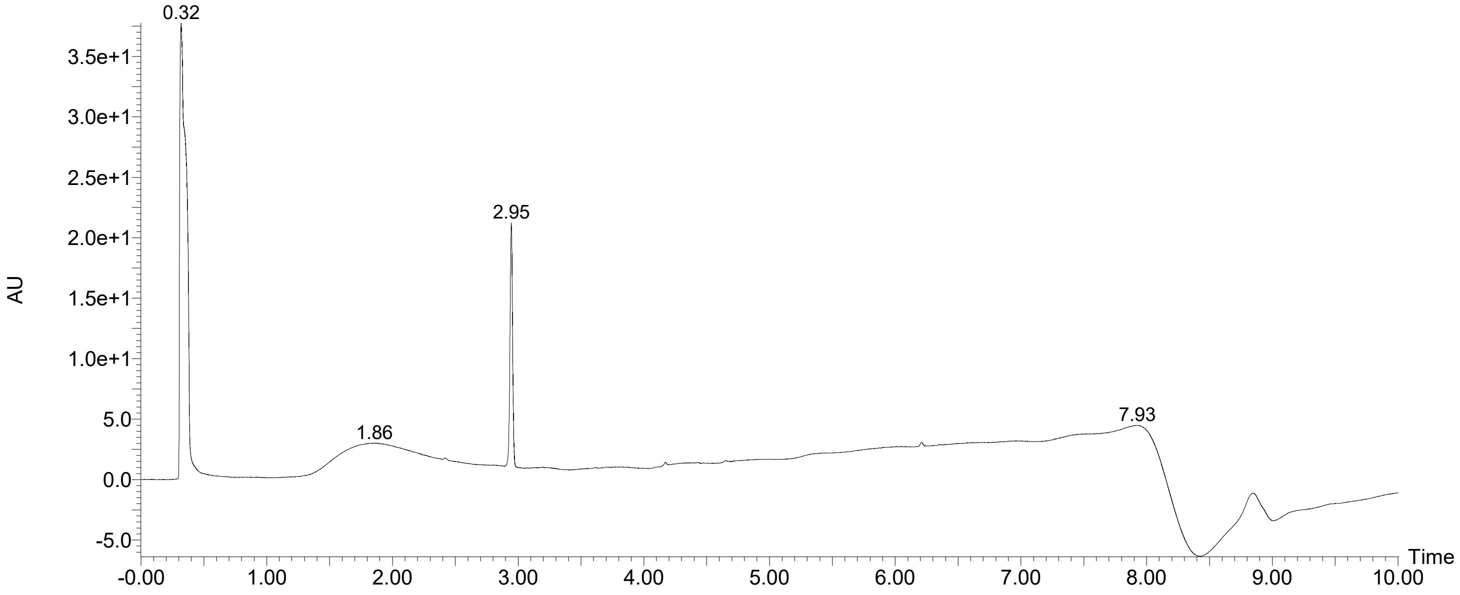
0.32
2.95
1.86
7.93
AU
3.5e+1
3.0e+1
2.5e+1
2.0e+1
1.5e+1
1.0e+1
5.0
0.0
-5.0
-0.00 1.00 2.00 3.00 4.00 5.00 6.00 7.00 8.00 9.00 10.00
Time

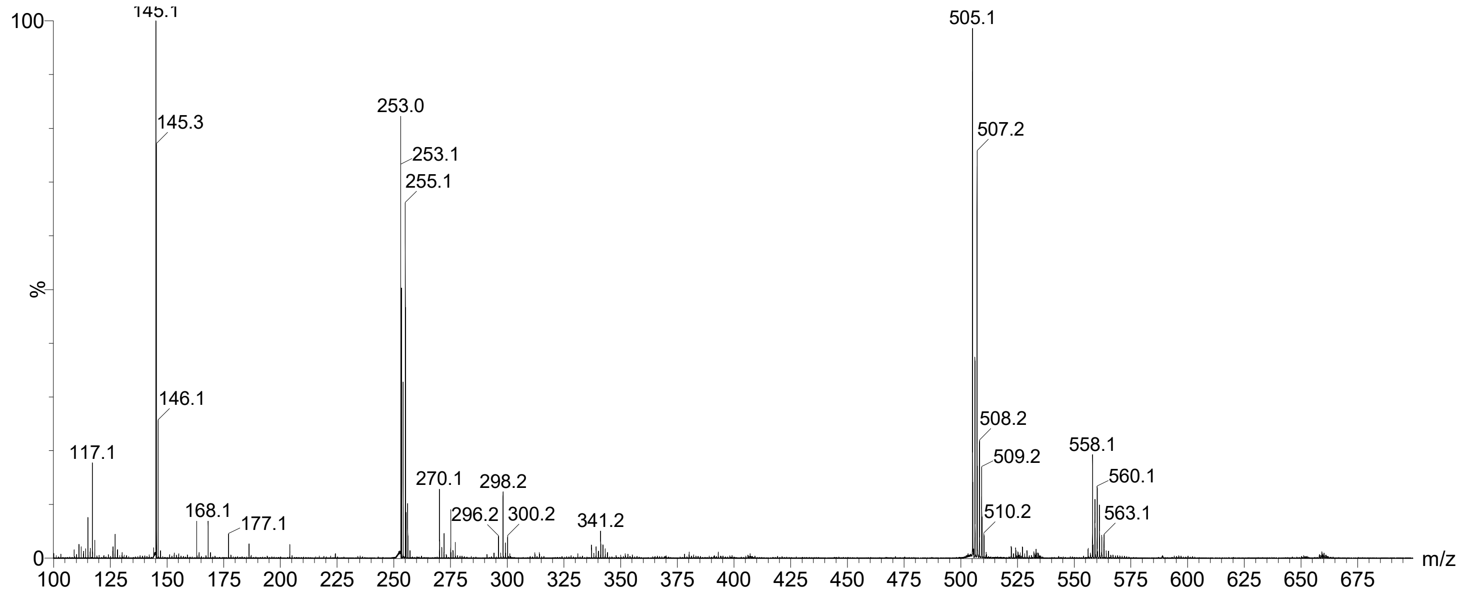
145.1
145.3
146.1
117.1
168.1
177.1
253.0
253.1
255.1
270.1
296.2
298.2
300.2
341.2
505.1
507.2
508.2
509.2
510.2
558.1
560.1
563.1
100
%
0
100 125 150 175 200 225 250 275 300 325 350 375 400 425 450 475 500 525 550 575 600 625 650 675
m/z

Compound 5

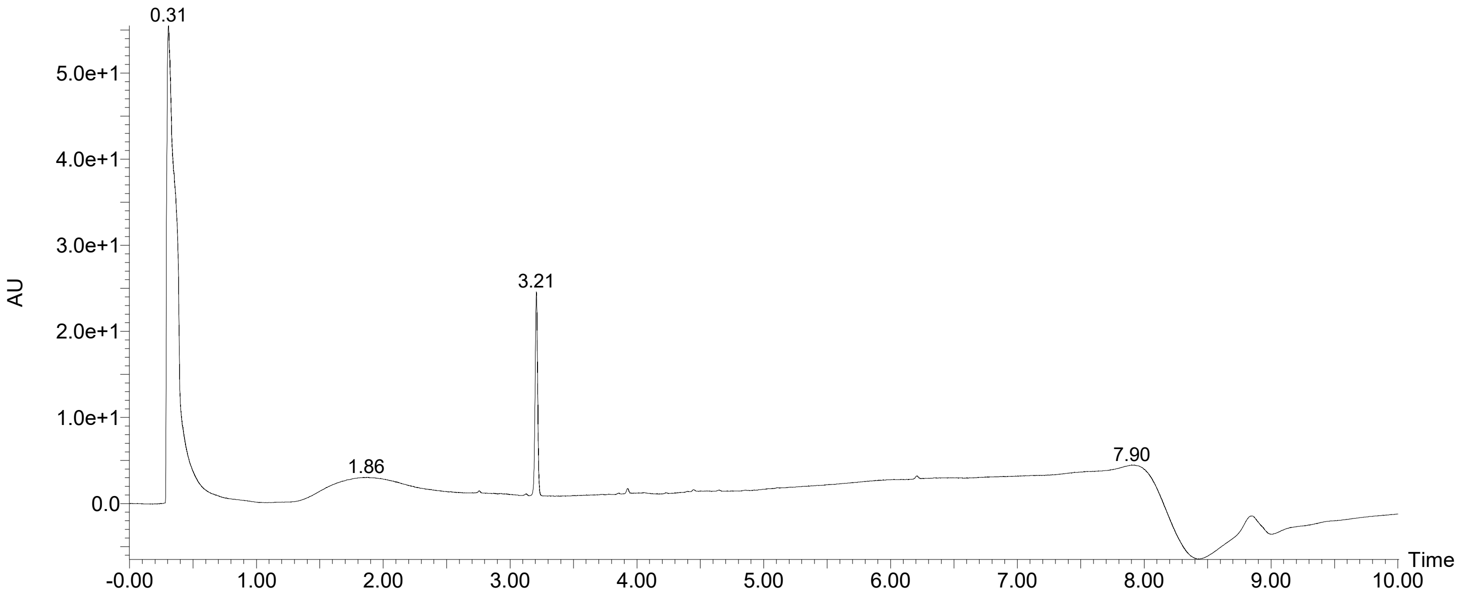

0.31
3.21
1.86
7.90
AU
Time
5.0e+1
4.0e+1
3.0e+1
2.0e+1
1.0e+1
0.0
-0.00
1.00
2.00
3.00
4.00
5.00
6.00
7.00
8.00
9.00
10.00

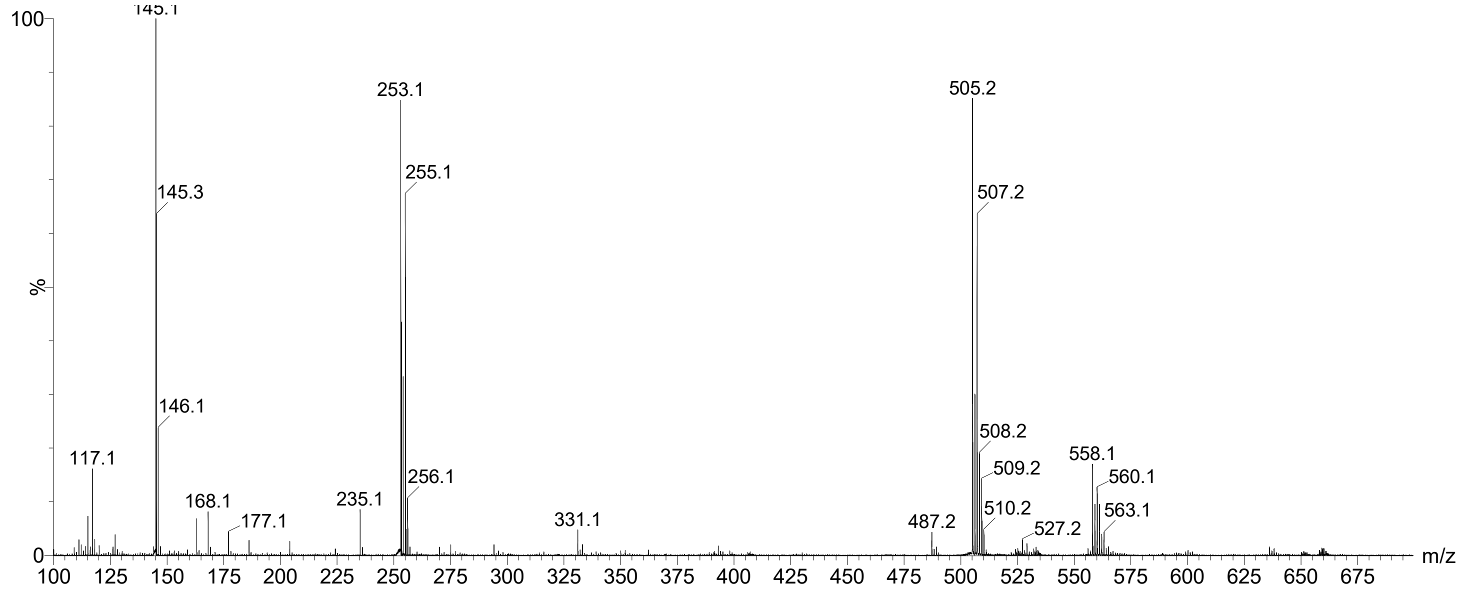

253.1
255.1
145.3
146.1
117.1
168.1
177.1
235.1
256.1
331.1
487.2
505.2
507.2
508.2
509.2
510.2
527.2
558.1
560.1
563.1
%
m/z
100
0

Compound 6

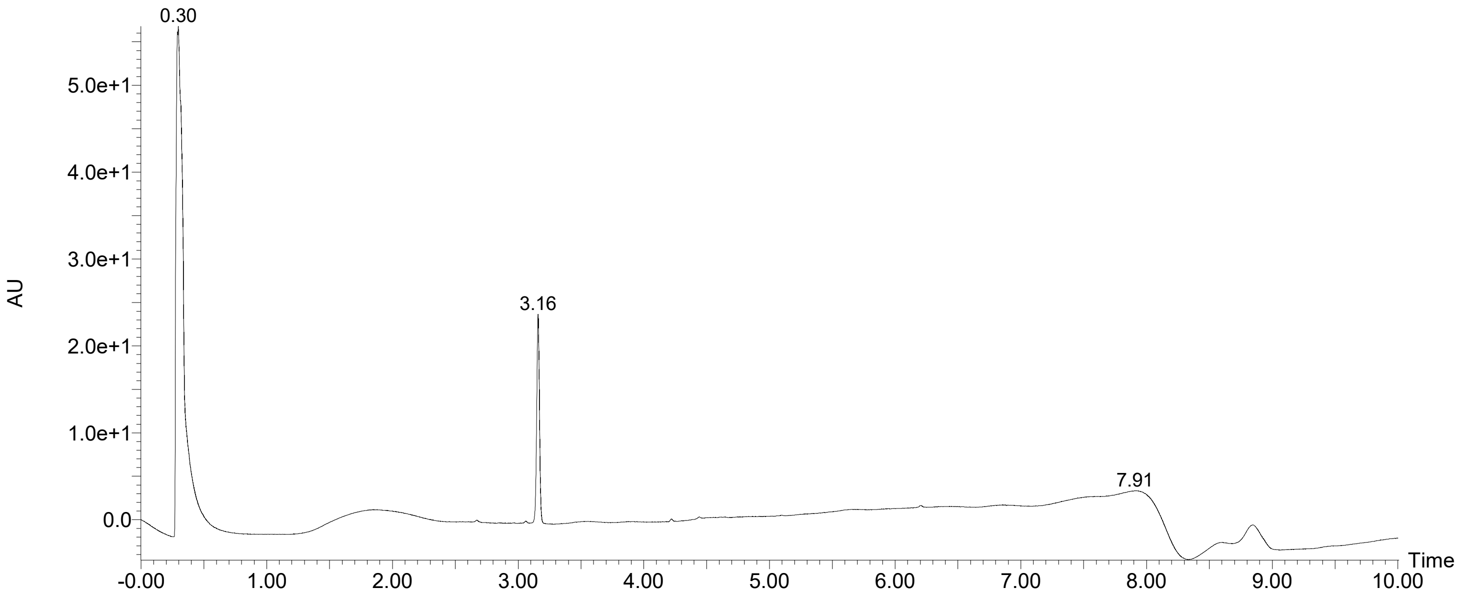
0.30
5.0e+1
4.0e+1
3.0e+1
AU
2.0e+1
3.16
1.0e+1
0.0
7.91
Time
-0.00 1.00 2.00 3.00 4.00 5.00 6.00 7.00 8.00 9.00 10.00

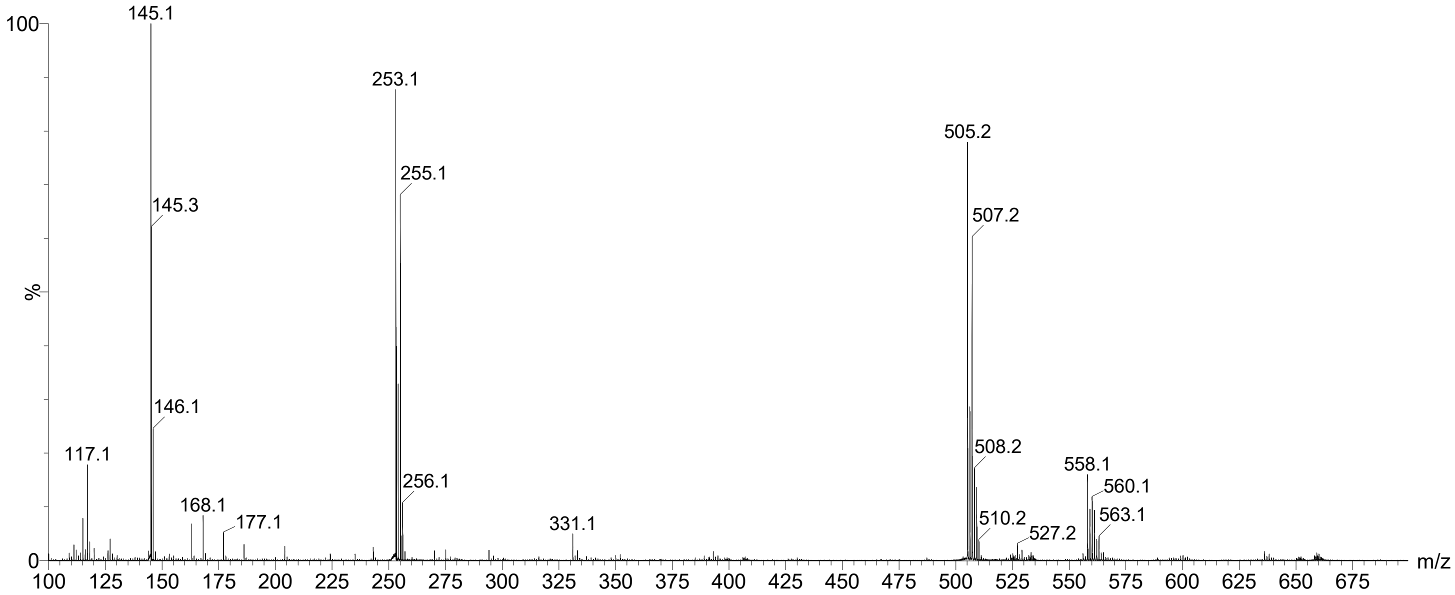
100
145.1
253.1
505.2
255.1
145.3
507.2
%
146.1
117.1
508.2
558.1
256.1
560.1
168.1
177.1
331.1
510.2
563.1
527.2
0
m/z
100 125 150 175 200 225 250 275 300 325 350 375 400 425 450 475 500 525 550 575 600 625 650 675

Compound 7

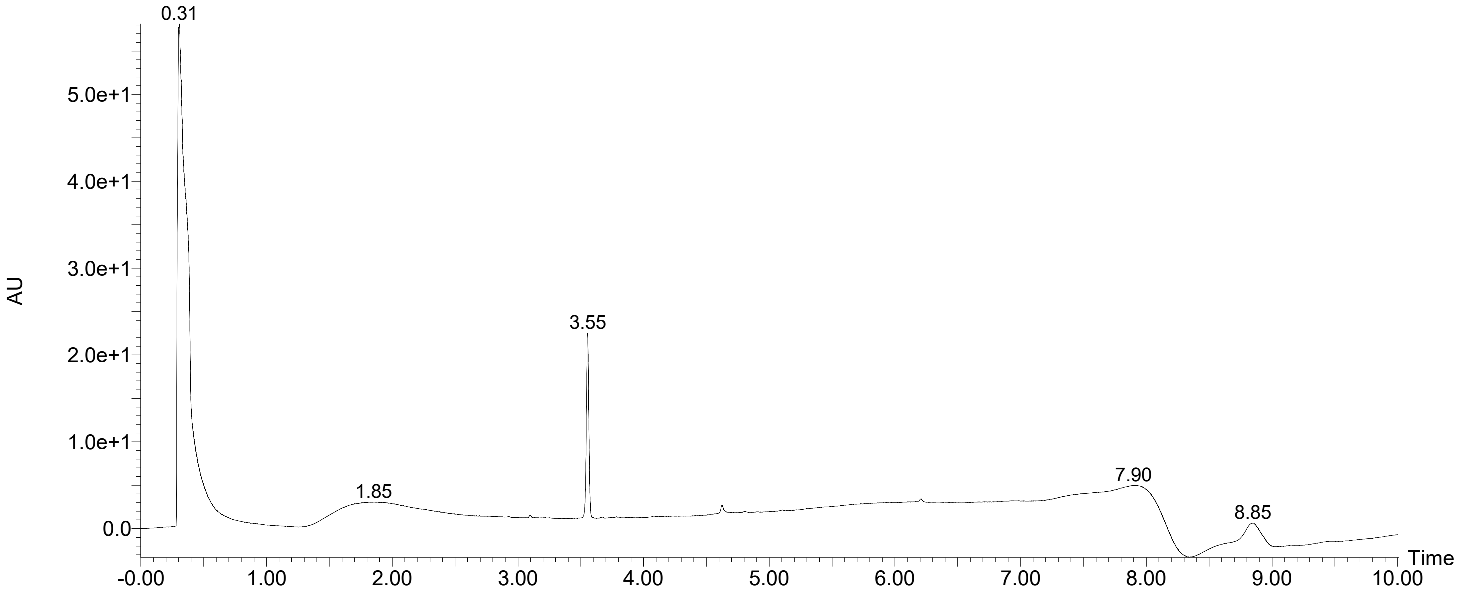

0.31
3.55
1.85
7.90
8.85
AU
5.0e+1
4.0e+1
3.0e+1
2.0e+1
1.0e+1
0.0
Time
-0.00
1.00
2.00
3.00
4.00
5.00
6.00
7.00
8.00
9.00
10.00

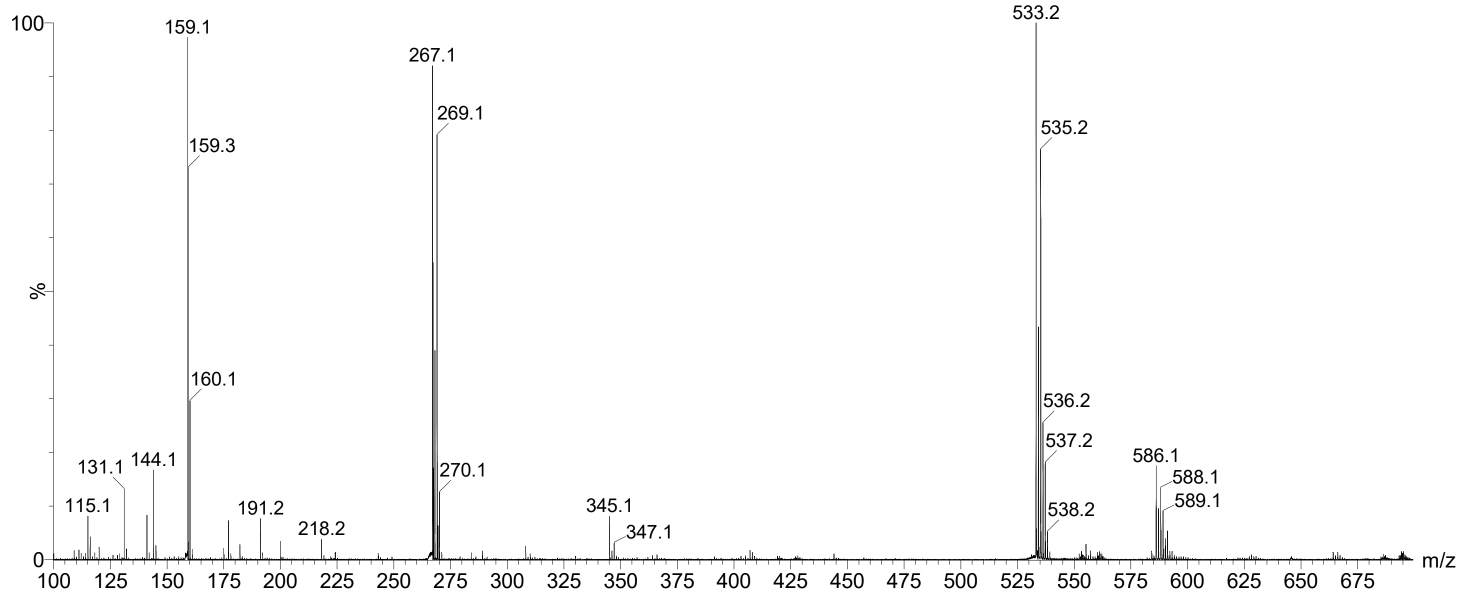

100
%
0
159.1
159.3
160.1
131.1
144.1
115.1
191.2
218.2
267.1
269.1
270.1
345.1
347.1
533.2
535.2
536.2
537.2
538.2
586.1
588.1
589.1
m/z
100
125
150
175
200
225
250
275
300
325
350
375
400
425
450
475
500
525
550
575
600
625
650
675

Compound 8, LN5P45

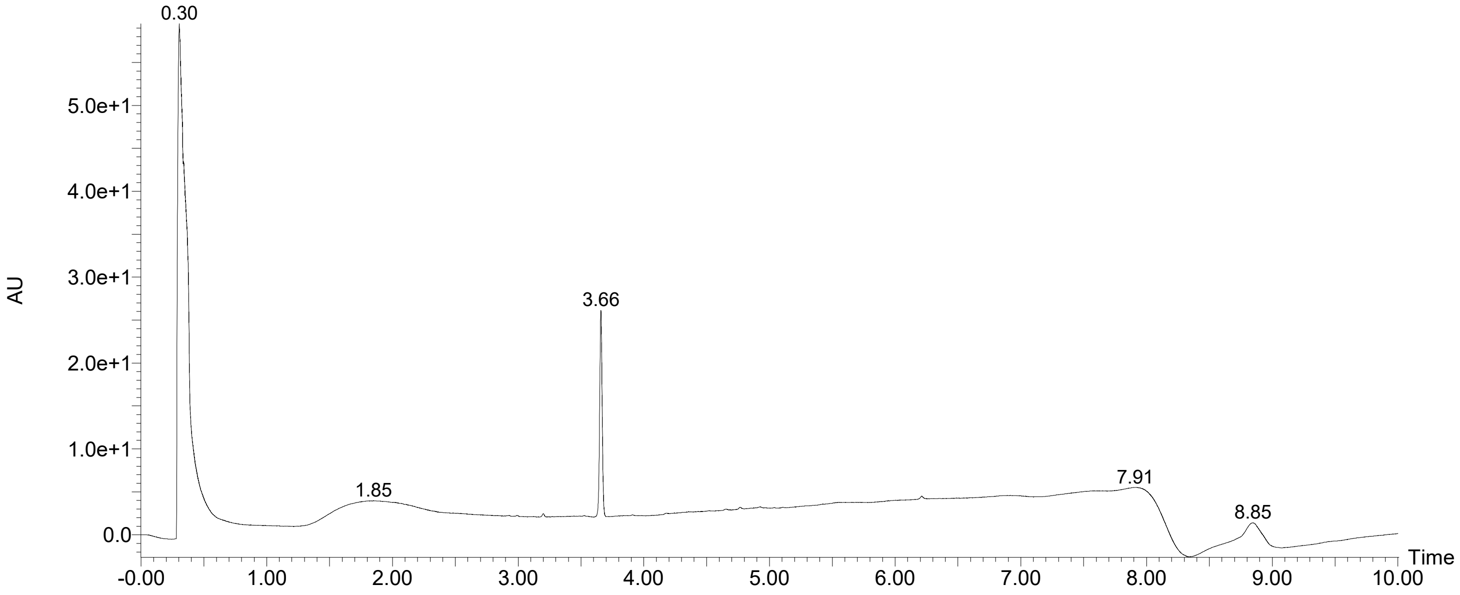

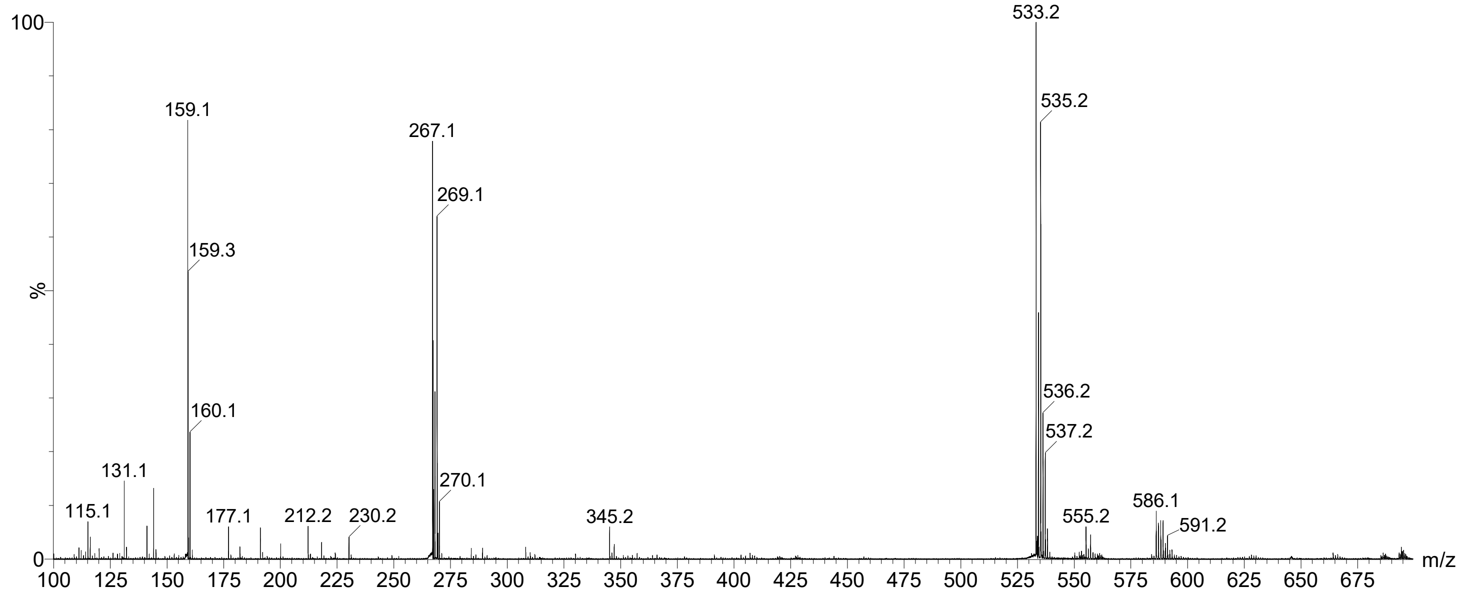

Compound 9

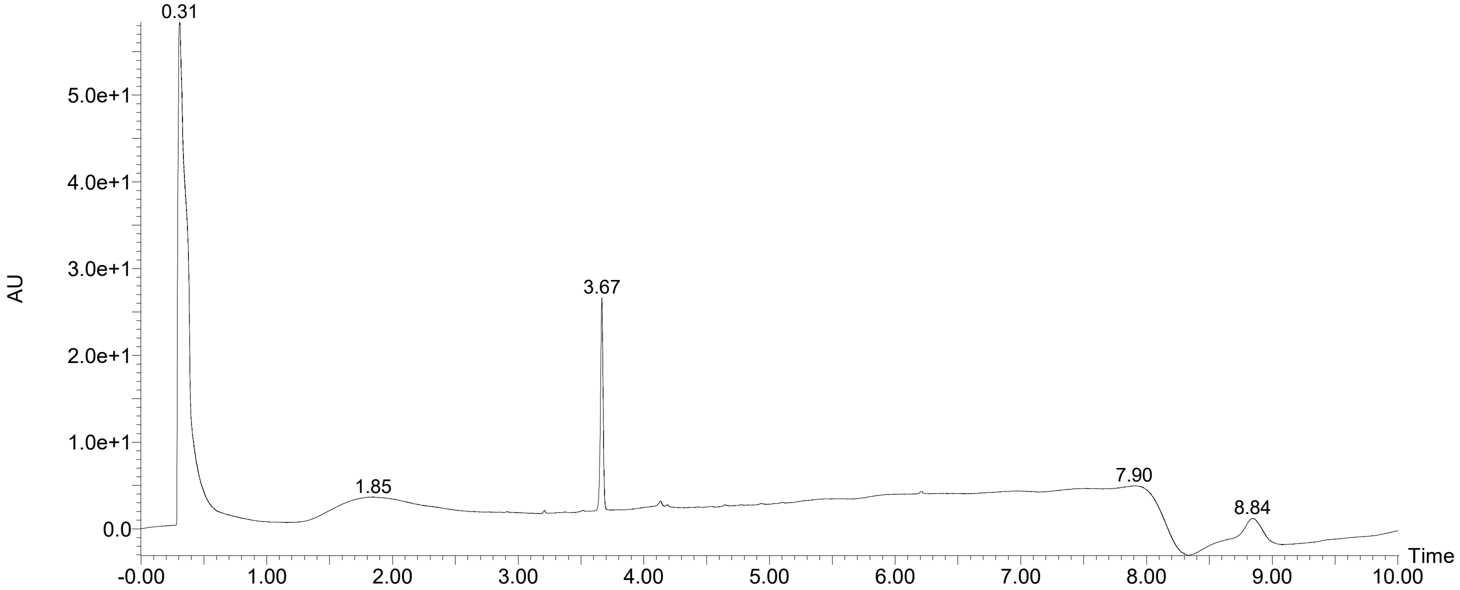
0.31
5.0e+1
4.0e+1
3.0e+1
2.0e+1
1.0e+1
0.0
AU
3.67
1.85
7.90
8.84
Time
-0.00
1.00
2.00
3.00
4.00
5.00
6.00
7.00
8.00
9.00
10.00

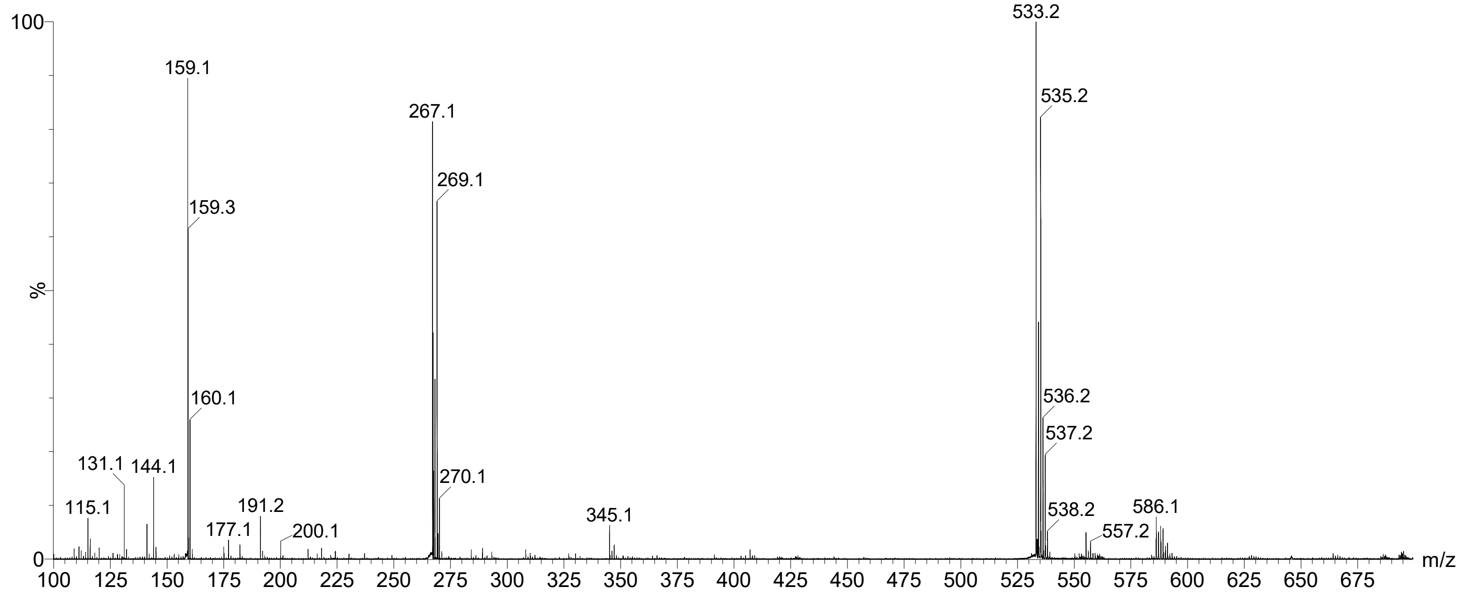
100
%
0
533.2
535.2
159.1
267.1
269.1
159.3
160.1
536.2
537.2
131.1
144.1
270.1
115.1
191.2
177.1
200.1
345.1
538.2
586.1
557.2
m/z
100
125
150
175
200
225
250
275
300
325
350
375
400
425
450
475
500
525
550
575
600
625
650
675

Compound 10

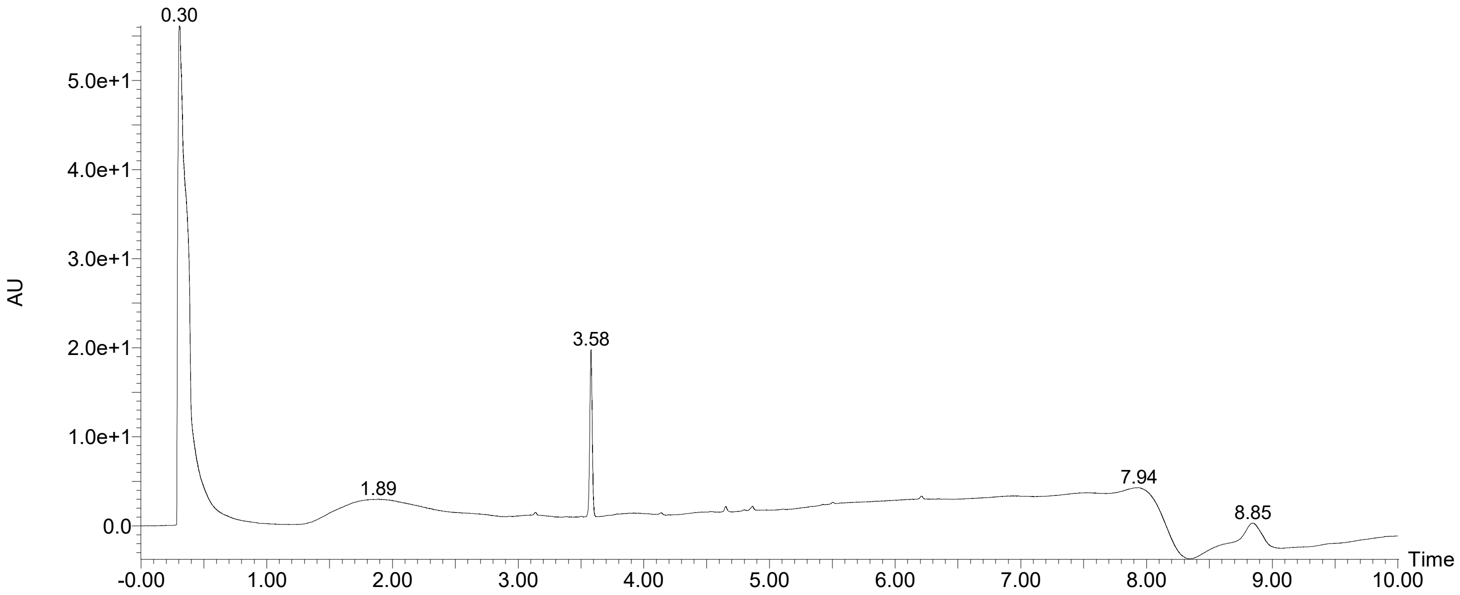

0.30
5.0e+1
4.0e+1
3.0e+1
AU
2.0e+1
1.0e+1
0.0
3.58
1.89
7.94
8.85
Time
-0.00
1.00
2.00
3.00
4.00
5.00
6.00
7.00
8.00
9.00
10.00

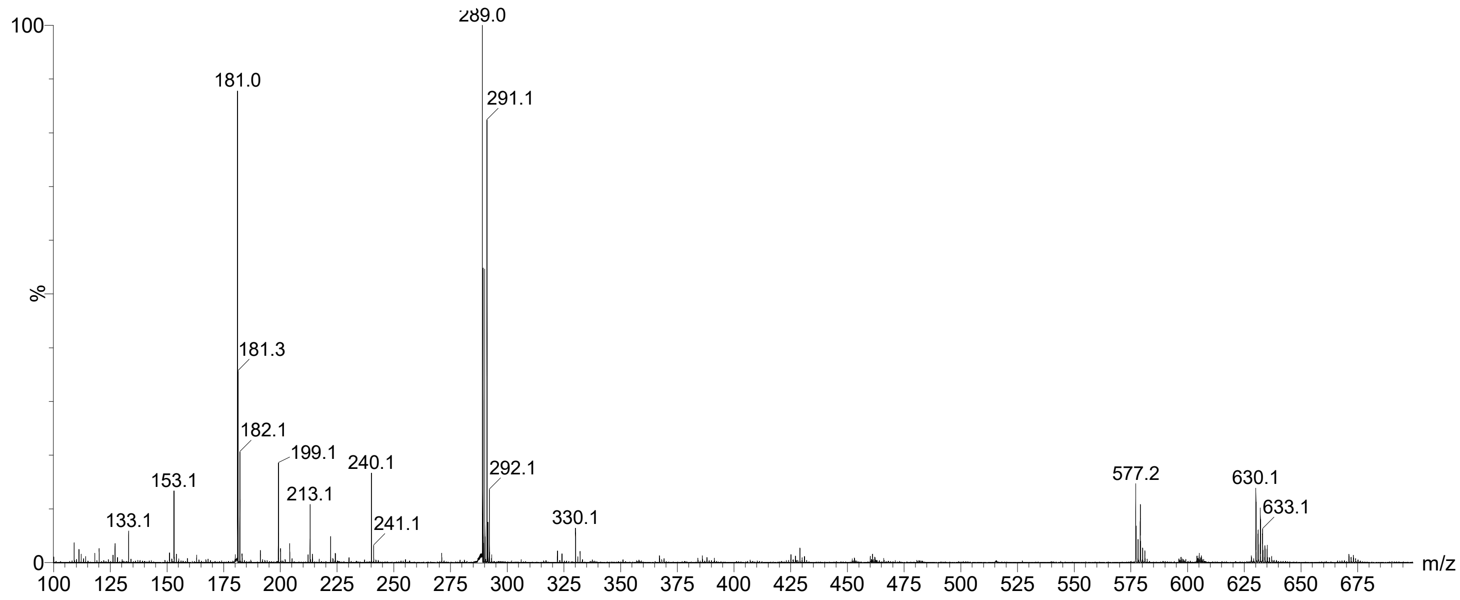

100
%
0
289.0
181.0
291.1
181.3
182.1
199.1
240.1
292.1
153.1
213.1
133.1
241.1
330.1
577.2
630.1
633.1
m/z
100 125 150 175 200 225 250 275 300 325 350 375 400 425 450 475 500 525 550 575 600 625 650 675
